# Chromophore Multiplication To Enable Exciton Delocalization and Triplet Diffusion Following Singlet Fission in Tetrameric Pentacene

**DOI:** 10.1002/anie.201907221

**Published:** 2019-09-18

**Authors:** Constantin Hetzer, Bettina S. Basel, Sebastian M. Kopp, Frank Hampel, Fraser J. White, Timothy Clark, Dirk M. Guldi, Rik R. Tykwinski

**Affiliations:** ^1^ Department of Chemistry and Pharmacy and Interdisciplinary Center for Molecular Materials (ICMM) Friedrich-Alexander-Universität Erlangen-Nürnberg (FAU) Nikolaus-Fiebiger-Strasse 10 91058 Erlangen Germany; ^2^ Department of Chemistry and Pharmacy and Interdisciplinary Center for Molecular Materials (ICMM), FAU Egerlandstrasse 3 91058 Erlangen Germany; ^3^ Department of Chemistry University of Alberta Edmonton Alberta T6G 2G2 Canada; ^4^ Rigaku Europe, Unit B6 Chaucer Business Park Watery Lane, Kemsing Sevenoaks TN15 6QY UK; ^5^ Computer Chemistry Center Department of Chemistry and Pharmacy, FAU Nägelsbachstrasse 25 91052 Erlangen Germany

**Keywords:** charge transfer, pentacene oligomers, singlet fission, time-resolved spectroscopy, triplet decorrelation

## Abstract

A tetrameric pentacene, **PT**, has been used to explore the effects of exciton delocalization on singlet fission (SF). For the first time, triplet decorrelation through intramolecular triplet diffusion was observed following SF. Transient absorption spectroscopy was used to examine different decorrelation mechanisms (triplet diffusion versus structural changes) for **PT** and its dimeric equivalent **PD** on the basis of the rate and activation barrier of the decorrelation step. Charge‐separation experiments using tetracyano‐*p*‐quinodimethane (**TCNQ**) to quench triplet excitons formed through SF demonstrate that enhanced intersystem crossing, that is, spin catalysis, is a widely underestimated obstacle to quantitative harvesting of the SF products. The importance of spatial separation of the decorrelated triplet states is emphasized, and independent proof that the decorrelated triplet pair state consists of two (T_1_) states per molecule is provided.

## Graphical abstract

**Not to be underestimated**: A tetrameric pentacene (see structure) was designed as a model system to explore the influence of exciton delocalization on singlet fission (SF) and subsequent triplet decorrelation by exciton diffusion following intramolecular SF. Experiment and theory showed the delocalization of singlet and triplet excitons among the four pentacenes and highlighted spin catalysis as a substantial obstacle to quantitative harvesting of SF products.

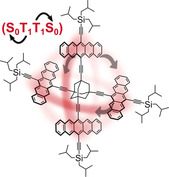

Singlet fission (SF) depends on the interaction of a photoexcited singlet‐state (S_1_) with a neighboring ground‐state (S_0_) chromophore, and results in the spontaneous splitting of (S_1_) into a pair of correlated triplets (T_1_T_1_).^1^ To harvest both triplet excitons, however, (T_1_T_1_) must transition into a pair of uncorrelated triplets (T_1_+T_1_). This decorrelation step is usually mediated by spin mixing with a quintet spin‐correlated triplet pair, ^5^(T_1_T_1_),^2^ as outlined in several excellent reviews.^3^

Successful SF requires a minimum of two chromophores, and dimers such as **PD** (Figure 1) have been studied in dilute solution to elucidate many of the mechanistic aspects of intramolecular SF (iSF). The power of organic synthesis is key and allows the electronic coupling between chromophores to be tuned through control of relative distance and orientation, as well as conjugation.^4^ Despite the success of dimers in dissecting the role of, for example, charge‐transfer, quintet, and excimer states,2b, 5 further breakthroughs in SF are currently hampered by the intrinsic limitations outlined below.

**Figure 1:**
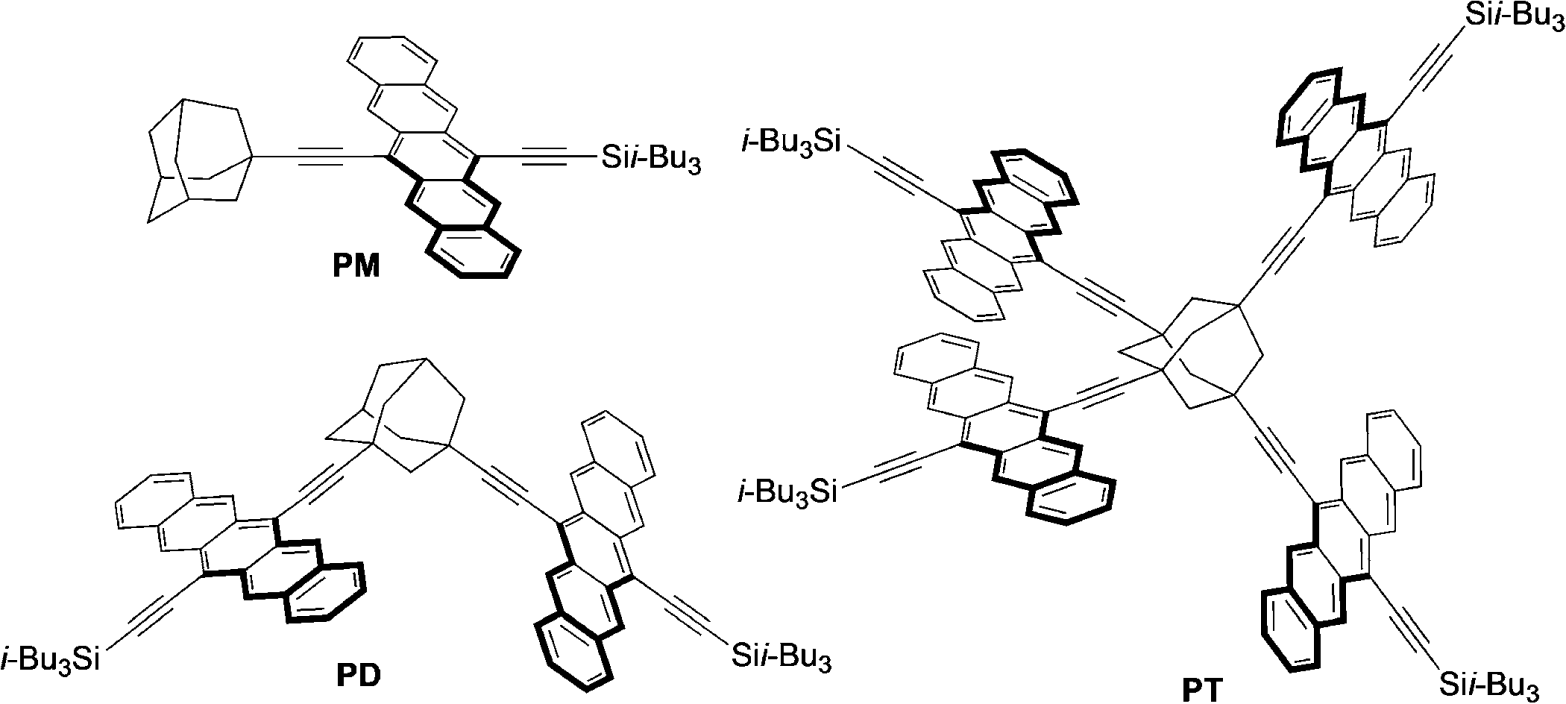
Structures of **PM**, **PT**, and **PD**.

In solids, intermolecular coupling allows diffusion of the initial singlet excitation over several molecules.3c Moreover, triplet decorrelation implies a distinct change in the coupling between triplets,2a, 6 facilitated by triplet diffusion.^6^, ^7^ The analogous diffusion is, however, impossible in molecular dimers, and triplet–triplet annihilation of (T_1_T_1_) usually governs recovery of (S_0_S_0_).^8^ Thus, oligomers and polymers have evolved as model systems for probing triplet diffusion.^9^ Nevertheless, after SF in a polymer, triplet diffusion from correlated triplets is hindered by strong coupling, which ultimately results in rapid recombination, rather than the desired formation of free triplets.

In the present study, we investigated the influence of chromophore multiplication on iSF. Chromophore multiplication differs from oligomerization: All chromophores remain electronically independent because of the nonconjugated spacer in **PT**. The adamantane scaffold is ideal for investigating chromophore multiplication via tetrameric **PT** (Figure 1).^10^ Through photophysical analysis and comparisons to dimeric **PD** and monomeric **PM**, supplemented with theoretical calculations, we identify differences in excitation behavior, SF, and triplet decorrelation that result from chromophore multiplication. Finally, we describe solution‐state “harvesting” of the products of SF through charge‐separation experiments with the electron acceptor tetracyano‐*p*‐quinodimethane (**TCNQ**). Importantly, charge‐separation experiments provide independent evidence that the decorrelated triplet pair state must consist of two non‐interacting triplets on the same molecule, rather than a single triplet per molecule. These studies demonstrate that enhanced intersystem crossing (EISC) hinders quantitative harvesting of the triplet products of SF. Thus, spatial triplet separation is now firmly established as a prerequisite for harvesting the two triplet excitons from SF efficiently.

Tetraethynyladamantane **1** was the key building block for pentacene tetramer **PT** (Scheme 1). A previously reported procedure for the synthesis of **1** 11 has several challenging steps; thus, an alternative synthetic route was developed. Starting from 1‐bromoadamantane, aldehyde **2** was formed in five steps (see the Supporting Information), and dibromoolefination gave intermediate **3**. Exhaustive elimination effected with BuLi, followed by aqueous workup, gave **1** through a Corey–Fuchs‐like reaction. Lithiation of **1** with LiHMDS gave intermediate **4**, to which a solution of ketone **5** was added. The reaction was subsequently quenched at low temperature by the addition of H_2_O. Without further purification, the intermediate was subjected to reductive aromatization.^12^ Purification by column chromatography gave tetramer **PT**. Tetramer **PT** has reasonable solubility (ca. 4 mg mL^−1^) in typical organic solvents (THF, CH_2_Cl_2_, CHCl_3_, toluene), but slowly decomposes over several days in solution upon exposure to air and light. In the absence of air (O_2_), solutions are stable for 3–5 days, and **PT** is stable as a solid under normal laboratory conditions.

**Scheme 1:**
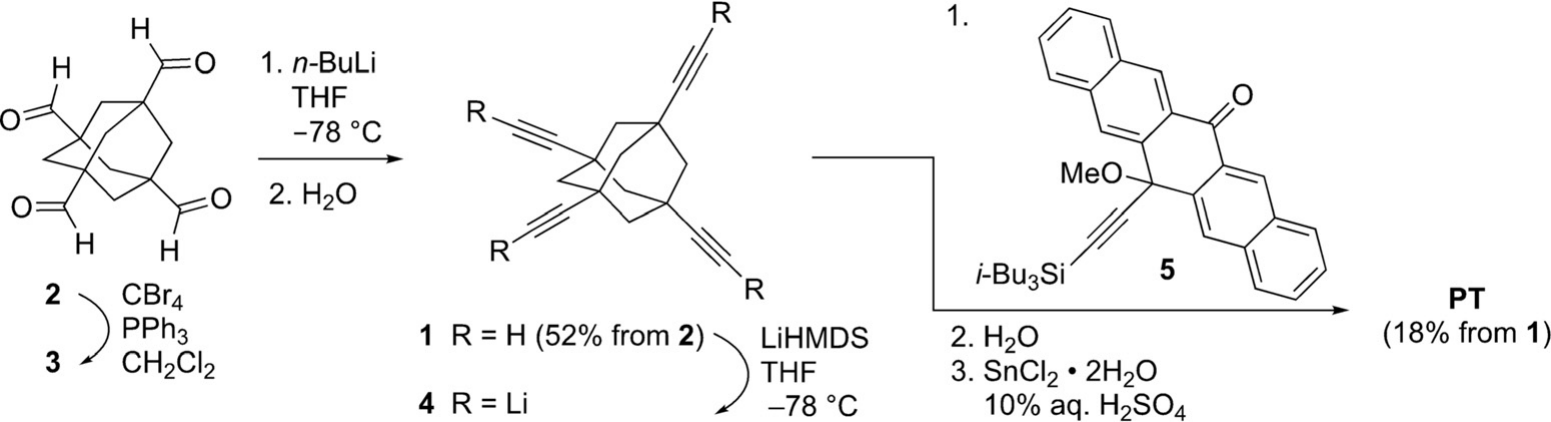
Synthesis of **PT**.

X‐ray crystallographic analysis of **PT** shows the symmetrical structure and demonstrates that there is no appreciable intramolecular π‐overlap between pentacene units in the solid state (Figure 2; see also the Supporting Information). Each pentacene moiety shows π‐stacking with that of a neighboring molecule with an interplanar distance between acene cores of 3.6 Å (see Figure S1 in the Supporting Information). π‐Stacking is limited to two acenes, however, and there is no long‐range π‐overlap.

**Figure 2:**
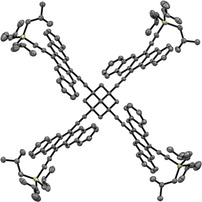
Solid‐state structure of **PT** showing the spatial arrangement of the pentacene units (H atoms omitted; ORTEPs at 30 % probability).

Steady‐state spectroscopy confirms that the pentacene moieties of **PD** and **PT** are electronically independent in the ground state: The intensities of the absorption bands evolve linearly as a function of the number of chromophores (see Figure S6). Closer inspection, however, reveals that the FWHM (full width at half maximum) decreases as a function of the number of pentacenes (see Table S1 in the Supporting Information). Upon normalization, the relative intensities of the fundamental 0–*0 transitions increase relative to those of the 0–*1, 0–*2, and 0–*3 transitions, and the increase depends on the number of pentacene groups (Figures 3 A,C). Thus, the Franck–Condon overlap for the 0–*0 transition increases with the number of chromophores. Fluorescence measurements show that the quantum yield is significantly lower in **PD** and **PT** than in **PM** (see Table S2). The Stokes shifts decrease in the order of **PM** > **PD** > **PT** (Figures 3 B,D), with a concurrent sharpening of the fluorescence spectra regardless of solvent polarity. In other words, the polarity of the excited singlet states is largest for **PM** and smallest for **PT**.

**Figure 3:**
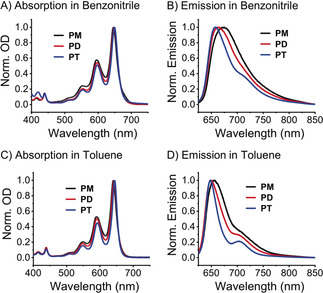
Normalized steady‐state absorption and fluorescence (excitation at 590 nm) spectra of **PM**, **PD**, and **PT**.

Femtosecond (fsTA) and nanosecond (nsTA) transient absorption experiments were used to investigate the excited‐state dynamics. A change in dipole moment upon excitation is associated with solvent relaxation during the first picoseconds after excitation, which is typically manifested as a blue shift of singlet excited‐state features in the near‐infrared (NIR) region (Figure 4 B).5a The energy gain upon relaxation (Δ*E*) decreases from **PM** to **PD** and to **PT** (Figure 4 B), consistent with the emission behavior. We conclude that the polarity of the singlet‐excited state decreases with the number of pentacenes, consistent with delocalization of the singlet‐excited state over all four pentacene moieties. Calculations support this hypothesis, showing only small splitting between the four singlet‐excited states that formally result from four locally excited pentacene singlets (see the Supporting Information). Minimal splitting, coupled with thermal motion, leads to time‐averaged delocalization (by Dexter energy transfer) of the singlet‐excited state. Note that this observation is supported by the fact that no Davidov splitting is observed in the experimental absorption spectra. Excited states S_1_–S_3_, which all have significant intensity, lie within 0.02 eV. Only S4, which is dark, is split significantly (0.14 eV) from the other three. The same is true for the four triplet states that formally result from localized pentacene triplet states. Thus, thermal mixing gives way to time‐averaged delocalization and reduced polarity on the timescale of solvent reorganization.

**Figure 4:**
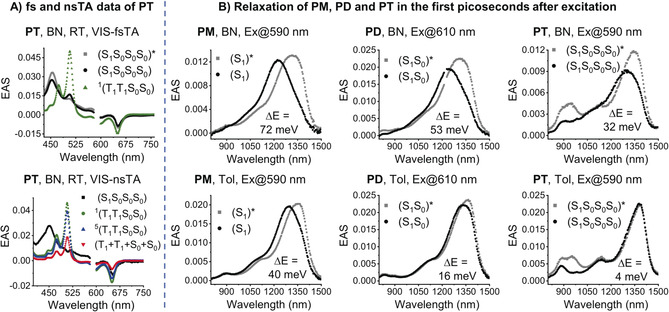
A) Evolution‐associated spectra (EAS) of the TA data of **PT** in argon‐saturated benzonitrile (BN; see Figure S7 for more details). B) EAS highlighting the blue shift of the fsTA NIR singlet feature of **PM**, **PD**, and **PT** in argon‐saturated BN and toluene (Tol). Ex=excitation wavelength.

On a longer timescale, the excited‐state dynamics of **PD** and **PT** are similar (Figure 4 A; see also Figures S7 and S8), and the singlet decay for both is significantly faster than for **PM** (see Table S3). In benzonitrile (BN), the singlet‐excited‐state lifetimes are 6.2 ns (S_1_), 402 ps (S_1_S_0_), and 129 ps (S_1_S_0_S_0_S_0_) in **PM**, **PD**, and **PT**, respectively, and reflect the statistical likelihood of iSF. Delocalized over four chromophores, iSF in **PT** offers six different pairs of pentacenes in a favored orientation: [(**T_1_T_1_**S_0_S_0_), (**T_1_**S_0_ **T_1_**S_0_), (**T_1_**S_0_S_0_ **T_1_**), (S_0_ **T_1_**S_0_ **T_1_**), (S_0_ **T_1_T_1_**S_0_), (S_0_S_0_ **T_1_T_1_**)], thus leading to a higher rate of iSF for **PT** than **PD**, as well as a concurrent increase in the rate constant of SF (*k*_SF_) by a factor of approximately 3, from 2.3×10^9^ (**PD**) to 6.8×10^9^ s^−1^ (**PT**). This finding is in line with results for oligomeric pentacenes.9b Solvent polarity also dictates *k*_SF_, with values in toluene of 1.2×10^9^ and 3.3×10^9^ s^−1^ for **PD** and **PT**, respectively, thus suggesting a superexchange mechanism.5a, 8a Finally, (S_1_S_0_S_0_S_0_) and (S_1_S_0_) decay, and the characteristic pentacene triplet‐excited state features evolve. Concurrent with singlet decay, ground‐state bleaching intensifies by a factor of approximately 2, which suggests nearly quantitative iSF for **PD** and **PT** (Figure 4 A; see also Figures S7 and S8), while **PM** produces <1 % triplet (see Figure S9). The triplet‐excited‐state decay is triexponential for **PD** and **PT** (**PD**/**PT**), including the singlet correlated triplet pairs ^1^(T_1_T_1_)/^1^(T_1_T_1_S_0_S_0_), the quintet correlated triplet pairs ^5^(T_1_T_1_)/^5^(T_1_T_1_S_0_S_0_), and the decorrelated triplet pairs (T_1_+T_1_)/(T_1_+T_1_+S_0_+S_0_).^13^ Triplet decorrelation lifetimes stand out. The ^5^(T_1_T_1_) lifetime of **PD** and ^5^(T_1_T_1_S_0_S_0_) of **PT** are 91 and 70 ns, respectively. If a localized pair of spin‐correlated triplets undergoes decorrelation through structural changes, then rates and yields of decorrelation should be essentially equal for **PT** and **PD** because of the analogous geometrical relationships between the pentacene chromophores. This is not the case, however, as the triplet excitations in ^5^(T_1_T_1_S_0_S_0_) are free to diffuse within the tetramer, which statistically affords more rapid decorrelation for **PT**. This behavior is also supported by the small splitting between the four relevant triplet states of **PT**, as supported by theoretical calculations (see above).

Temperature‐dependent nsTA measurements for **PD** and **PT** in BN have been performed (see Figures S13–S19) to provide the activation energy (*E*_A_) for triplet decorrelation (Figure 5). Triplet decorrelation arises through a change in coupling between the two triplet states. In the solid state, it occurs as two triplets diffuse away from each other. In a dimer, changes in coupling would be caused by, for example, bond rotations. Triplet decorrelation in **PD** faces a barrier of approximately 13 meV, consistent with the geometric rearrangement. Decorrelation in **PT**, however, lacks any appreciable temperature dependence (*E*_A_=0), and an alternative mechanism must thus be operative: Decorrelation occurs through diffusion of a triplet excitation to any of the pentacenes in a ground‐state configuration with a slightly different conformation than that of the initially excited triplet.

**Figure 5:**
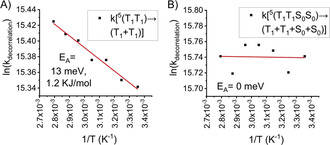
Arrhenius plots of the triplet decorrelation rate *k*_decorrelation_ for A) **PD** and B) **PT** (see Table S5 for details).

For SF to be implemented in solar energy conversion schemes, it is imperative that both triplets can be harvested. Toward this goal, charge‐separation experiments with the electron acceptor tetracyano‐*p*‐quinodimethane (**TCNQ**) were attempted; an excess of **TCNQ** (6.3–200 equiv) was used relative to **PD** or **PT** to guarantee pseudo‐first‐order reaction conditions. In the presence of **TCNQ**, both **PT** and **PD** show accelerated decay of the triplet features at 508 nm (see Figure S24) and concomitant growth of new features at 700–900 nm, which are spectroscopic fingerprints of **TCNQ^.−^** (see Figures S20–S24).^14^

Sequential analyses of the nsTA data yielded good fits of the transient kinetics using five transient species for **PD**/**PT**: (S_1_S_0_)/(S_1_S_0_S_0_S_0_), ^1^(T_1_T_1_)/^1^(T_1_T_1_S_0_S_0_), ^5^(T_1_T_1_)/^5^(T_1_T_1_S_0_S_0_), (T_1_+T_1_)/(T_1_+T_1_+S_0_+S_0_), and the charge‐separated state (P^.+^ + **TCNQ^.−^**), in which (P^.+^) is the one‐electron‐oxidized form of pentacene (see Figures S26–S31). A sequential fit demonstrates that the charge‐separated state (P^.+^ + **TCNQ^.−^**) is predominantly populated by a diffusion‐controlled reaction of **TCNQ** with (T_1_+T_1_)/(T_1_+T_1_+S_0_+S_0_). Nevertheless, a minor contribution stems from interactions of **TCNQ** with the quintet state ^5^(T_1_T_1_)/^5^(T_1_T_1_S_0_S_0_).

Target analyses of the corresponding data for **PT** and **PD** were then performed assuming that two charge‐separated states (P^.+^ + **TCNQ^.−^**) evolved from ^5^(T_1_T_1_)/^5^(T_1_T_1_S_0_S_0_) or (T_1_+T_1_)/(T_1_+T_1_+S_0_+S_0_) to yield [(P^.+^+P^.+^)/(P^.+^+P^.+^+S_0_+S_0_) + 2 **TCNQ^.−^**] (see Figure S33). Under this assumption, the quantum yield for electron transfer (ET) to **TCNQ** was approximately 50 % relative to (T_1_+T_1_)/(T_1_+T_1_+S_0_+S_0_). In other words, one charge‐separated state, rather than two, was generated per (T_1_+T_1_)/(T_1_+T_1_+S_0_+S_0_). The fact that (P^.+^+T_1_)/(P^.+^+T_1_+S_0_+S_0_) is harder to oxidize than (T_1_+T_1_)/(T_1_+T_1_+S_0_+S_0_) is not fully compatible with our observations. Considering that only a single triplet in (T_1_+T_1_)/(T_1_+T_1_+S_0_+S_0_) is oxidized, the spectroscopic signatures of the charge‐separated state (P^.+^ + **TCNQ^.−^**) should be discernable in concert with those of the second triplet, which is not the case. Rather, the triplet decay goes hand‐in‐hand with the formation of the (P^.+^ + **TCNQ^.−^**) features, that is, both triplets in (T_1_+T_1_)/(T_1_+T_1_+S_0_+S_0_) deactivate simultaneously. Nevertheless, charge separation is only 50 %. We conclude that (P^.+^) accelerates deactivation of the triplet‐excited state of the neighboring pentacene; thus, a rate increase of ISC is induced by a neighboring unpaired electron, through enhanced intersystem crossing (EISC) or spin catalysis.^15^ Finally, a revised target analysis considered the formation of only one charge‐separated state per ^5^(T_1_T_1_)/ ^5^(T_1_T_1_S_0_S_0_) or (T_1_+T_1_)/(T_1_+T_1_+S_0_+S_0_) to afford [(P^.+^+S_0_)/(P^.+^+S_0_+S_0_+S_0_) + **TCNQ^.−^**] (Figure 6; see also Figures S35–S39), which gives a quantum yield for ET of essentially 100 % relative to (T_1_+T_1_)/(T_1_+T_1_+S_0_+S_0_) for all data sets. Thus, only one of the two triplets is quenched, and the other is deactivated to the ground state due to EISC.

**Figure 6:**
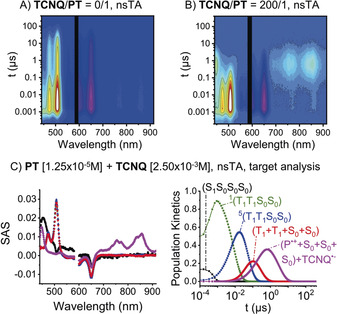
A) nsTA raw data for a)**PT** in argon‐saturated BN. B) nsTA raw data for **PT** and **TCNQ** in argon‐saturated BN. C) Species‐associated spectra (SAS) and corresponding population kinetics of the nsTA data shown in (B) as obtained by target analysis (see Figure S34 for the kinetic model and Table S14 for rate constants).

In conclusion, we show that the tetrameric structure of **PT** is an excellent model system for SF in solids beyond the two‐chromophore approximation of dimers. Solvent‐dependent experiments establish that the singlet‐excited state of **PT** is delocalized (from a time‐averaged perspective) over four pentacenes by thermal mixing, as observed in crystalline chromophores.3c Similar to (T_1_T_1_) found in **PD**, coupling in (T_1_T_1_S_0_S_0_) for **PT** is sufficiently weak to facilitate triplet decorrelation. The mechanisms of triplet decorrelation in **PD** and **PT** are, however, different. In **PD**, temperature‐dependent TA measurements demonstrate that decorrelation is triggered by geometric rearrangements, whereas in **PT** triplet diffusion occurs most likely by Dexter‐type energy transfer resulting in time‐averaged delocalization. These conclusions are supported by both experiment and theory. Experiments with **TCNQ** demonstrate that, following formation of the decorrelated triplet pair, trapping of one triplet by charge separation leads to accelerated decay of the second triplet to the ground state by EISC. Our results establish that efficient spatial diffusion of the triplet states is a crucial parameter toward harvesting both triplet excitons.

## Conflict of interest

The authors declare no conflict of interest.

## Supporting information

As a service to our authors and readers, this journal provides supporting information supplied by the authors. Such materials are peer reviewed and may be re‐organized for online delivery, but are not copy‐edited or typeset. Technical support issues arising from supporting information (other than missing files) should be addressed to the authors.

Supplementary
