# Supplementary material for: Chromophore Multiplication To Enable Exciton Delocalization and Triplet Diffusion Following Singlet Fission in Tetrameric Pentacene
Source: Angew Chem Int Ed Engl. 2019 Sep 18;58(43):15263–7. doi: 10.1002/anie.201907221 (PMC7497398; doi:10.1002/anie.201907221)
Supplement: Supplementary file 1 — Supplementary [file ANIE-58-15263-s001.pdf]

Angewandte Chemie
Eine Zeitschrift der Gesellschaft Deutscher Chemiker

Supporting Information

Chromophore Multiplication To Enable Exciton Delocalization and Triplet Diffusion Following Singlet Fission in Tetrameric Pentacene

Constantin Hetzer[+], Bettina S. Basel[+], Sebastian M. Kopp, Frank Hampel, Fraser J. White, Timothy Clark, Dirk M. Guldi,* and Rik R. Tykwinski**

anie_201907221_sm_miscellaneous_information.pdf

Table of Contents

Table of Contents **1**

Experimental Procedures **2**

General description of chemicals, materials and instrumentation for synthesis 2

Crystallography 2

Photophysical Analysis 2

Spectroscopy procedures 2

Determination of extinction coefficients *2*

Spectroelectrochemistry *3*

Transient absorption setup *3*

Analysis of the TA data 3

Global Analysis using a sequential model *3*

Calculation of the charge-transfer quantum efficiency from global analyses *4*

Determination of the second order charge-transfer rate constant from global analysis *5*

Target Analysis of the electron transfer experiments under the assumption that PD / PT can be oxidized twice by reacting with two TCNQ molecules *5*

Target Analysis of the electron transfer experiments under the assumption that PD / PT can be oxidized only once by reacting with TCNQ. *6*

Electrochemistry 7

Computational methods 7

Supplemental synthesis procedures 8

Supplemental Results **11**

NMR Spectra 12

Supplemental photophysical data 16

Steady-state characterization 16

Room temperature (RT) transient absorption (TA) spectroscopy 17

Temperature dependent TA spectroscopy 24

Electron transfer studies 33

Sequential analysis of the charge-transfer reaction of PD and PT with TCNQ *38*

Target analysis of the charge-transfer reaction of ***PD*** *and* ***PT*** *with* ***TCNQ*** *under the assumption that* ***PD / PT*** *can be oxidized twice by reacting with two* ***TCNQ*** *molecules* *46*

Target analysis of the charge-transfer reaction of ***PD*** *and* ***PT*** *with* ***TCNQ*** *under the assumption that* ***PD*** */* ***PT*** *can be oxidized only once by reacting with* ***TCNQ****.* *49*

Electrochemistry 62

Supplemental computational results 63

Excited states 69

References **74**

Author Contributions **74**

Experimental Procedures

General description of chemicals, materials and instrumentation for synthesis

Reagents were purchased reagent grade from commercial suppliers and used without further purification. Compounds **5**[1] and 6,13-bis(tri-*iso*butylsilylethynyl)pentacene (**TIBS**)[2] were synthesized as reported. THF was distilled from Na/benzophenone ketyl. All reactions were performed in standard dry glassware. $MgSO_4$ was used as the drying agent after aqueous workup. Evaporation and concentration *in vacuo* was done at water-aspirator pressure. Brine refers to a saturated aqueous solution of NaCl. ^1H and ^{13}C spectra were recorded on a Bruker Avance instrument at 300 MHz (^1H) and 100 MHz (^{13}C) and an Agilent/Varian u500 at 498 MHz (^1H) and 126 MHz (^{13}C). NMR spectra were referenced to the residual solvent signal (1H: $CDCl_3$, 7.24 ppm; CD_2Cl_2, 5.32 ppm; ^{13}C: $CDCl_3$, 77.0 ppm) and recorded at ambient temperature. $CDCl_3$ and CD_2Cl_2 were stored over 4 Å molecular sieves. Coupling constants are reported as observed (±0.5 Hz). UV-vis measurements were carried out on a Varian Cary 5000 UV-vis-NIR spectrophotometer at rt. Mass spectra were obtained from a Bruker maxis 4G (APPI, ESI) or a Bruker 9.4T Apex-Qe FTICR (MALDI) instruments. IR spectra were recorded as solids on Varian 660-IR spectrometers in ATR-mode or as CH_2Cl_2 cast film on a Thermo Nicolet 8700 FTIR spectrometer with continuum FTIR microscope. Melting points were measured with an Electrothermal 9100 instrument or a 6406-K Thomas-Hoover melting point apparatus. TLC analyses were carried out on TLC plates from Marchery-Nagel (Alugram® SIL G/UV_{254}) and visualized via UV-light (264/364 nm) or standard coloring reagents. Column chromatography was performed using Silica Gel 60M (Merck). Differential scanning calorimetry (DSC) measurements were made on a Mettler Toledo TGA/ STDA 851e/1100/SF. DSC measurements were carried out under a flow of nitrogen with a heating rate of 10 °C/min. Melting points from DSC are reported as the peak maxima; decomposition points were reported with their onset temperature and the exothermic maxima corresponding to the decomposition.

Crystallography

X-ray data for **PT** (CCDC 1915771) has been deposited at the Cambridge Crystallographic Data Centre (CCDC), 12 Union Road, Cambridge CB21EZ, UK; fax: (+44)122-333-6033. These data can be obtained free of charge from The Cambridge Crystallographic Data Centre via the Internet at www.ccdc.cam.ac.uk/data_request/cif using the CCDC number given above.

Photophysical Analysis

Spectroscopy procedures

All solvents were purchased from commercial suppliers and used without further purification. Steady-state UV-vis absorption spectra in toluene and benzonitrile were acquired at room temperature (RT) using a Perkin Elmer Lambda 2 spectrometer. Steady-state fluorescence spectra were carried out at a FluoroMax3 spectrometer from Horiba in the visible detection range (RT). Fluorescence quantum yields were measured relative to a zinc phthalocyanine used as a reference compound.[3]

Determination of extinction coefficients

About 1.5 mg of the solid sample were weighed accurately to 0.1 mg and dissolved in reagent grade CH_2Cl_2 (250 mL) in a volumetric flask. Solution state UV-measurements were performed at room

temperature on a Varian Cary 400 UV-vis double beam spectrophotometer (**PM**) or a Varian Carry 5000 UV-vis-NIR spectrophotometer (**PD** and **PT**). Measurements were carried out with quartz glass sample and reference cuvettes (10.0 mm diameter). The recorded data were blanc value corrected. The reported molar extinction coefficients ε were calculated as average molar extinction coefficients from three independently recorded absorption spectra with a deviation of less than 10%.

Spectroelectrochemistry

Solutions of analytes were prepared in argon-saturated solvents containing 0.1 M TBA[PF]$_6$ as electrolyte. Spectra were recorded with a Varian/Agilent Cary 5000 (**TCNQ**) or using the AvaLight-DH-S-BAL Balanced Power Light Source from Avantes in combination with the Avantes NIRLine detector AvaSpec-NIR-1.7 and the Avantes StarLine detector AvaSpec-2048 (**PM**). A three-electrode setup with platinum gauze as working electrode, Pt wire as counter electrode and Ag wire as pseudoreference electrode was used. Potentials were provided by aMetrohm Autolab PGSTAT101, controlled via Metrohm AutolabNova 1.10 software.

Transient absorption setup

The EOS and HELIOS spectrometers (Ultrafast Systems) were used for broadband pump-probe nanosecond and femtosecond transient absorption (TA) experiments. Shot-by-shot acquisition and balanced detection using the probe-reference method were applied. The output of an amplified Ti:Sapphire CPA-2110 fs laser system (Clark MXR: output 775 nm, 1 kHz, 150 fs pulse width, standard deviation <0.5%) is used to generate excitation wavelengths (590 and 610 nm) with a noncolinear optical parametric amplifier (NOPA, Clark MXR, standard deviation <2%). In HELIOS experiments (fsTA), the probe was generated by focusing the fundamental of the CPA-2110 fs laser system on a sapphire crystal (standard deviation < 2%). For EOS measurements (nsTA), the probe pulse was generated by a pulsed supercontinuum laser (fundamental: 1064 nm, output 350 - 2200 nm, 2 kHz, 700 ps–1 ns pulse width, standard deviation < 1%) that is part of the EOS spectrometer.

Analysis of the TA data

All raw fsTA data were baseline, chirp- and zero-point-corrected for presentation in the manuscript and SI. All presented nsTA data were corrected for scattered light. All corrections for presentation were done with Ultrafast`s Surface Xplorer.

Global and target analysis of the TA data was performed with the open-source software package Glotaran.[4] Prior to global and target analysis, a baseline correction (fsTA data), respectively, a correction for scattered light (nsTA data) was performed with Ultrafast`s Surface Xplorer. The wavelength dependent character (dispersion) of the instrument response function (IRF) was modeled in global and target analysis and taken into account.

Global Analysis using a sequential model

A sequential model implies that, for the global fit, the conversion rate from one species to the next species is set to 100%. For the TA data analyzed by global analysis with a sequential model the quantum yields were calculated as described elsewhere.[5]

The SF rate of **PD** and **PT** was calculated under the assumption that all processes other than SF that lead to a decay of the singlet excited state (fluorescence, internal conversion, intersystem crossing, etc.) possess the same rate in the reference compound **PM** as in the dimer **PD** and tetramer **PT**. Thus, k_{SF} was calculated using Fermi`s golden rule as:

$$k_{SF}(\boldsymbol{PD}) = \frac{1}{\tau[(S_1S_0)]} - \frac{1}{\tau[(S_1)]} \qquad \text{(S1)}$$

$$k_{SF}(\boldsymbol{PT}) = \frac{1}{\tau[(S_1S_0S_0S_0)]} - \frac{1}{\tau[(S_1)]} \quad \text{(S2)}$$

Whereby $\tau[(S_1S_0)]$ is the lifetime of singlet excited state (S_1S_0) of **PD**, $\tau[(S_1S_0S_0S_0)]$ is the lifetime of singlet excited state ($S_1S_0S_0S_0$) of **PT** and $\tau(S_1)$ is the lifetime of the singlet excited state (S_1) of **PM** in the same solvent, at the same temperature and in dilute solution.
The triplet-decorrelation rate $k_{decorrelation}$ is calculated as:

$$k_{decorrelation}(\boldsymbol{PD}) = \frac{1}{\tau[^5(T_1T_1)]} \times \frac{c[(T_1+T_1)]}{c[^5(T_1T_1)]} \quad \text{(S3)}$$

$$k_{decorrelation}(\boldsymbol{PT}) = \frac{1}{\tau[^5(T_1T_1S_0S_0)]} - \frac{c[(T_1+T_1+S_0+S_0)]}{c[^5(T_1T_1S_0S_0)]} \quad \text{(S4)}$$

Where $\tau[^5(T_1T_1)]$ / $\tau[^5(T_1T_1S_0S_0)]$ is the lifetime of correlated triplet pair state with quintet spin $^5(T_1T_1)$ / $^5(T_1T_1S_0S_0)$ of **PD** / **PT** and $\frac{c[(T_1+T_1)]}{c[^5(T_1T_1)]}$ / $\frac{c[(T_1+T_1+S_0+S_0)]}{c[^5(T_1T_1S_0S_0)]}$ is the quantum yield of the triplet decorrelation [$^5(T_1T_1)$ / $^5(T_1T_1S_0S_0)$ → (T_1+T_1) / (T_1+T_1+S_0+S_0)] of **PD** / **PT**.

Calculation of the charge-transfer quantum efficiency from global analyses
There are two different possibilities to define the observed charge-transfer reaction. If we assume that the intrinsic ISC rate of a remaining (T_1) is unaffected by a neighboring ($P^{•+}$) in a (T_1+$P^{•+}$) / (T_1+$P^{•+}$+S_0+S_0) state, both (T_1) states can undergo charge transfer independently with two different TCNQ molecules to yield two pentacene cations, ($P^{•+}$), and two TCNQ anions, (TCN TCNQ$^{•-}$) per decorrelated triplet state (T_1+T_1) / (T_1+T_1+S_0+S_0). The charge-transfer reaction would then be defined as:

$$(T_1+T_1) + 2\times TCNQ \rightarrow (P^{\bullet+}+P^{\bullet+}) + 2\times TCNQ^{\bullet-} \quad \text{(S5)}$$

$$(T_1+T_1+S_0+S_0) + 2\times TCNQ \rightarrow (P^{\bullet+}+P^{\bullet+}+S_0+S_0) + 2\times TCNQ^{\bullet-} \quad \text{(S6)}$$

A more correct view of the CT reaction would, however, be:

$$(T_1+T_1) + TCNQ \rightarrow (P^{\bullet+}+S_0) + TCNQ^{\bullet-} \quad \text{(S7)}$$

$$(T_1+T_1+S_0+S_0) + TCNQ \rightarrow (P^{\bullet+}+S_0+S_0+S_0) + TCNQ^{\bullet-} \quad \text{(S8)}$$

Because intrinsic ISC rate of a remaining (T_1) was found to be strongly accelerated by a neighboring ($P^{•+}$) in a (T_1+$P^{•+}$) / (T_1+$P^{•+}$+S_0+S_0) state. Consequently, the formation of a (T_1+$P^{•+}$) / (T_1+$P^{•+}$+S_0+S_0) state is faster than the deactivation of the remaining (T_1) to (T_1+S_0) / (T_1+S_0+S_0+S_0). Therefore, the CT rate k_{CT} is equal for both discussed cases.
Applying pseudo first order kinetics, the charge-transfer rate k_{CT} of the reaction of the decorrelated triplet (T_1+T_1) / (T_1+T_1+S_0+S_0) with TCNQ can be calculated using Fermi`s golden rule as:

$$k_{CT}(\boldsymbol{PD}) = \frac{1}{\tau[(T_1+T_1)+TCNQ]} - \frac{1}{\tau[(T_1+T_1)]} \quad \text{(S9)}$$

$$k_{CT}(\boldsymbol{PT}) = \frac{1}{\tau[(T_1+T_1+S_0+S_0)+TCNQ]} - \frac{1}{\tau[(T_1+T_1+S_0+S_0)]} \quad \text{(S10)}$$

Whereby $\tau[(T_1+T_1)+TCNQ]$ is the lifetime of decorrelated triplet state (T_1+T_1) of **PD** upon addition of TCNQ, $\tau[(T_1+T_1+S_0+S_0)+TCNQ]$ is the lifetime of decorrelated triplet state (T_1+T_1+S_0+S_0) of **PT**

upon addition of TCNQ, and $\tau[(T_1 + T_1)]$ is the lifetime of decorrelated triplet state (T_1+T_1) of **PD** without TCNQ, $\tau[(T_1 + T_1 + S_0 + S_0)]$ is the lifetime of decorrelated triplet state (T_1+T_1+S_0+S_0) of **PT** without TCNQ, all in the same solvent, at the same temperature, in dilute solution and with a large excess of TCNQ.

With k_{CT} in hands, the charge-transfer quantum efficiency QE_{CT} can be calculated:

$$QE_{CT}(\boldsymbol{PD}) = k_{CT}(\boldsymbol{PD}) \div \frac{1}{\tau[(T_1 + T_1) + TCNQ]} \quad \text{(S11)}$$

$$QE_{CT}(\boldsymbol{PT}) = k_{CT}(\boldsymbol{PT}) \div \frac{1}{\tau[(T_1 + T_1 + S_0 + S_0) + TCNQ]} \quad \text{(S12)}$$

Please note: In the context of this work, the quantum efficiency of a process is calculated based on Fermi`s golden rule using rate constants. The quantum yield of a process is always calculated using ΔOD values, as described elsewhere.[5]

Determination of the second order charge-transfer rate constant from global analysis

This section is based on the remarks in the textbook "Photochemistry and Photophysics: Concepts, Research, Applications" by V. Balzani et al.[6]

The second order charge-transfer rate constant k_2 may not be mixed up with the pseudo first order charge-transfer rate k_{CT}. Considering the quenching reaction of TCNQ with a decorrelated triplet state of **PD** as seen in equations (S7), the rate of consumption of (T_1+T_1), v, will be:

$$v = k_2 \times c(TCNQ) \times c(T_1 + T_1) + k_{ISC} \times c(T_1 + T_1) = (k_{CT} + k_{ISC}) \times c(T_1 + T_1) \quad \text{(S13)}$$

Whereby k_{ISC} is the intrinsic deactivation rate of a decorrelated triplet state (T_1). If the TCNQ concentration is much larger than the (T_1+T_1) concentration (pseudo first order conditions), the relative changes in the TCNQ concentration during the reaction can be neglected. Then, $k_2 \times c(TCNQ)$ is summarized as the pseudo first order charge-transfer rate k_{CT}.

The second order charge-transfer rate can be determined by plotting the inverse lifetime of (T_1+T_1) upon addition of TCNQ, k_{obs}, - measured in different pseudo first order experiments - over the TCNQ concentration in these experiments:

$$k_{obs} = k_2 \times c(TCNQ) + k_{ISC} \quad \text{(S14)}$$

k_2 can then be calculated as the slope of a linear fit of these data points.

These considerations can be transferred to the decorrelated triplet pair state (T_1+T_1+S_0+S_0) of **PT** and the quintet correlated triplet pair state $^5(T_1T_1)$ / $^5(T_1T_1S_0S_0)$ of **PD** or **PT**. In the case of $^5(T_1T_1)$ / $^5(T_1T_1S_0S_0)$ k_{ISC} needs to be complemented by the decorrelation rate.

Target Analysis of the electron transfer experiments under the assumption that PD / PT can be oxidized twice by reacting with two TCNQ molecules

We established a kinetic model for global analysis of the nsTA data of the electron transfer reaction under the assumption that a $^5(T_1T_1)$ / $^5(T_1T_1S_0S_0)$ or (T_1+T_1) / (T_1+T_1+S_0+S_0) state can be oxidized twice by reacting with two TCNQ molecules. For this the lifetime of each state was first extracted from global analysis with 5 sequential transients: the singlet excited state (S_1S_0) / ($S_1S_0S_0S_0$) state after solvent relaxation, the correlated triplet pair state with singlet spin $^1(T_1T_1)$ / $^1(T_1T_1S_0S_0)$, the correlated triplet pair state with quintet spin $^5(T_1T_1)$ / $^5(T_1T_1S_0S_0)$, the decorrelated triplet state (T_1+T_1) / (T_1+T_1+S_0+S_0) and the charge separated state [($P^{\bullet+}$+$P^{\bullet+}$) / ($P^{\bullet+}$+$P^{\bullet+}$+S_0+S_0) + 2 x $TCNQ^{\bullet-}$], whereby $P^{\bullet+}$ is the pentacene cation. The optimized lifetimes were then used in global analyses, while the individual rates for specific deactivation channels were allowed to vary. The fit was deemed satisfactory when (i) the species-

associated spectra of $^{1}(T_1T_1)$ / $^{1}(T_1T_1S_0S_0)$, $^{5}(T_1T_1)$ / $^{5}(T_1T_1S_0S_0)$ and (T_1+T_1) / $(T_1+T_1+S_0+S_0)$ were of similar intensity at all wavelengths, and (ii) the species-associated spectrum of (S_1S_0) / $(S_1S_0S_0S_0)$ possessed about half the negative ΔOD value at the ground-state bleaching of the species-associated spectra of $^{1}(T_1T_1)$ / $^{1}(T_1T_1S_0S_0)$, $^{5}(T_1T_1)$ / $^{5}(T_1T_1S_0S_0)$, (T_1+T_1) / $(T_1+T_1+S_0+S_0)$ and $[(P^{\bullet+}+P^{\bullet+})$ / $(P^{\bullet+}+P^{\bullet+}+S_0+S_0) + 2$ x $TCNQ^{\bullet-}]$.

For simplification reasons, we did not include delayed fluorescence in this model. Please note, that the decay of the charge separated state $[(P^{\bullet+}+P^{\bullet+})$ / $(P^{\bullet+}+P^{\bullet+}+S_0+S_0) + 2$ x $TCNQ^{\bullet-}]$ is not a pseudo first order reaction. The extracted lifetime of this state is, therefore, meaningless.

Target Analysis of the electron transfer experiments under the assumption that PD / PT can be oxidized only once by reacting with TCNQ.

We established a kinetic model for global analysis of the nsTA data of the electron transfer reaction under the assumption that a $^{5}(T_1T_1)$ / $^{5}(T_1T_1S_0S_0)$ or (T_1+T_1) / $(T_1+T_1+S_0+S_0)$ state can be oxidized only once by reacting with TCNQ, as a neighboring cation $(P^{\bullet+})$ increases the ISC rate of the remaining triplet in a $(P^{\bullet+}+T_1)$ / $(P^{\bullet+}+T_1+S_0+S_0)$ state. For this the lifetime of each state was first extracted from global analysis with 5 sequential transients: the singlet excited state (S_1S_0) / $(S_1S_0S_0S_0)$ state after solvent relaxation, the correlated triplet pair state with singlet spin $^{1}(T_1T_1)$ / $^{1}(T_1T_1S_0S_0)$, the correlated triplet pair state with quintet spin $^{5}(T_1T_1)$ / $^{5}(T_1T_1S_0S_0)$, the decorrelated triplet state (T_1+T_1) / $(T_1+T_1+S_0+S_0)$ and the charge separated state $[(P^{\bullet+}+S_0)$ / $(P^{\bullet+}+S_0+S_0+S_0) + TCNQ^{\bullet-}]$. The optimized lifetimes were then used in global analyses, while the individual rates for specific deactivation channels were allowed to vary. The fit was deemed satisfactory when (i) the species-associated spectra of $^{1}(T_1T_1)$ / $^{1}(T_1T_1S_0S_0)$, $^{5}(T_1T_1)$ / $^{5}(T_1T_1S_0S_0)$ and (T_1+T_1) / $(T_1+T_1+S_0+S_0)$ were of similar intensity at all wavelengths, and (ii) the species-associated spectrum of (S_1S_0) / $(S_1S_0S_0S_0)$ possessed about half the negative ΔOD value at the ground-state bleaching of the species-associated spectra of $^{1}(T_1T_1)$ / $^{1}(T_1T_1S_0S_0)$, $^{5}(T_1T_1)$ / $^{5}(T_1T_1S_0S_0)$, and (T_1+T_1) / $(T_1+T_1+S_0+S_0)$ and (iii) the species-associated spectrum of (S_1S_0) / $(S_1S_0S_0S_0)$ possessed a similar negative ΔOD value at the ground-state bleaching as the species-associated spectrum of $[(P^{\bullet+}+S_0)$ / $(P^{\bullet+}+S_0+S_0+S_0) + TCNQ^{\bullet-}]$.

For simplification reasons, we did not include delayed fluorescence in this model. Please note, that the decay of the charge separated state $[(P^{\bullet+}+P^{\bullet+})$ / $(P^{\bullet+}+P^{\bullet+}+S_0+S_0) + 2$ x $TCNQ^{\bullet-}]$ is not a pseudo first order reaction. The extracted lifetime of this state is, therefore, meaningless.

Electrochemistry

Electrochemical measurements were performed with an EC Epsilon^{TM} potentiostat/ galvanostat (**PM**), a BAS CV-50W Version 2 (**PD** and **PT**), or a Metrohm FRA 2 µAutolab Type III potentiostat equipped with an internal impedance unit (**TCNQ**). A single-compartment, three-electrode cell configuration was used for cyclic voltammetry (CV) measurements, using for **PM**, **PD** and **PT** a glassy carbon electrode (3.0 mm diameter) as working electrode, a platinum wire as counter and an Ag/$AgNO_3$ electrode as pseudo reference electrode. For **TCNQ** a platinum electrode (1mm diameter) was used as a working electrode, a platinum wire as a counter and a silver wire as pseudo reference electrode. CV was performed in HPLC-grade CH_2Cl_2 (**PM**, **PD** and **PT**) or in spectroscopic grade benzonitrile (**TCNQ**) containing 0.1 M TBA$[PF]_6$ as electrolyte at a scan rate of 100 (**PM**, **TCNQ**) or 150 mV/s (**PD** and **PT**). The potential of the redox couple ferrocene/ferrocenium (Fc/Fc^+) was measured right before the samples and used as reference. The potential values E for reversible and quasi-reversible processes were calculated by the following equation $E = (E_{pc} + E_{pa}) / 2$, where E_{pc}, and E_{pa} correspond to the cathodic or anodic peak potentials, respectively. Potentials for irreversible processes were estimated by reporting E_{pc} or E_{pa}.

Computational methods

For simplicity, $^iPr_3Si$ groups were replaced by Me_3Si to avoid additional conformational complexity. B3LYP[7]/6-31G(d)[8] density-functional theory (DFT) with Grimme's D3 dispersion correction[9] was used to investigate the energetics of the conformational rearrangements of the pentacene moieties in the tetra-substituted derivative (**PT**). Geometries were fully optimized and stationary points characterized by calculating their normal vibrations within the harmonic approximation. Energy profiles for rearrangement of the relative orientations of the pentacenes were carried out by driving dihedral angles between one of the C-C bond to the relevant bridgehead in the adamantane and a ring bond to the *ipso*-carbon in the pentacene. Excited-state calculations were performed with semiempirical configuration interaction using both single (CIS) and single and double excitations (CISD) and the AM1 Hamiltonian.[10] All DFT calculations used Gaussian 16[11] and semiempirical calculations were performed with the in-house version of EMPIRE.[12]

Supplemental synthesis procedures

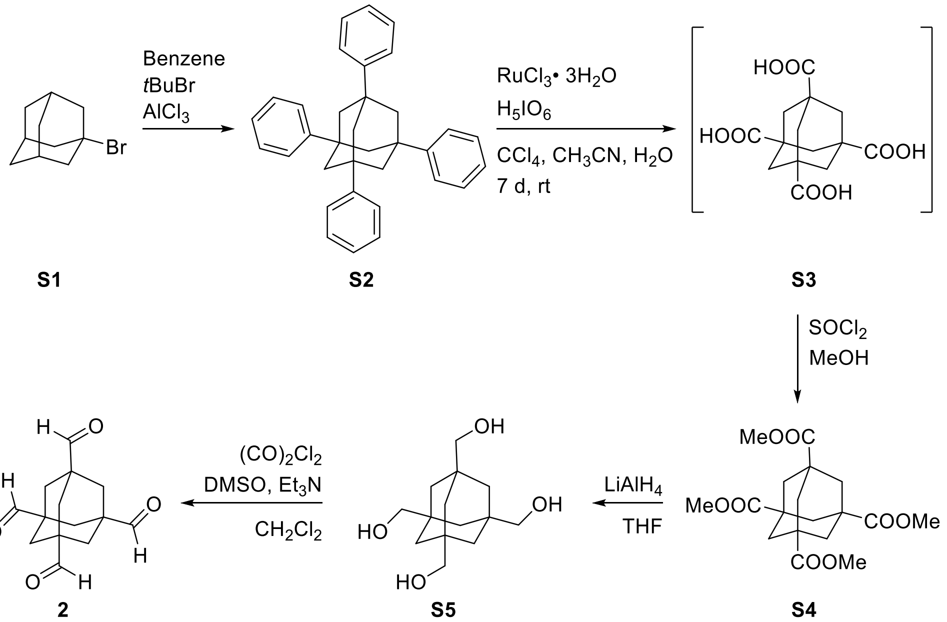

Scheme 1. Syntheses toward building block **2**. Compound **S2**,[13] and **S3–S4**[14] were synthesized as reported. The transformations from **S4** to **S5**[15] and from **S5** to **2**[15] were accomplished as reported.

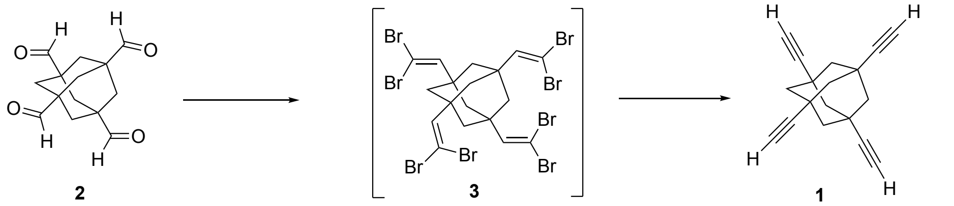

Compound 1: At 0 °C, CBr_4 (3.69 g, 11.1 mmol) was dissolved in dry, deoxygenated CH_2Cl_2 (100 mL), and PPh_3 (6.09 g, 23.2 mmol) was added within 15 min as a solid. The mixture was allowed to warm to rt and stirred for 30 min. A solution of **2** (0.300 g, 1.21 mmol) in dry, deoxygenated CH_2Cl_2 (20 mL) was added dropwise *via* cannula over 15 min and the mixture was allowed to stir for 18 h. Hexanes (200 mL) was added and the resulting suspension was filtered through a pad of silica gel (hexanes), washed with hexanes (3 × 50 mL), and the solvent removed *in vacuo*. The resulting solid, **3**, was directly subjected to elimination without further purification.

At –78 °C, the crude dibromoolefin **3** (1.04 g, 1.20 mmol) was dissolved in dry, deoxygenated THF (40 mL) and *n*-BuLi (4.10 mL, 10.3 mmol, 2.5 M in hexanes) was added dropwise *via* cannula over 10 min. The mixture was stirred at –78 °C for 1 h and then for 1.5 h at rt. Satd. aq. NH_4Cl (100 mL) was added, and the aqueous phase was extracted with CH_2Cl_2 (3 × 50 mL). The combined organic phases were washed with water (100 mL), brine (100 mL), dried ($MgSO_4$), and the solvent removed *in vacuo*. Column chromatography (silica, CH_2Cl_2/hexanes 1:1) afforded **1** (0.146 g, 52% based on **2**) as a colorless liquid, which solidified upon standing. Physical and spectroscopic data were consistent with those reported.[16]

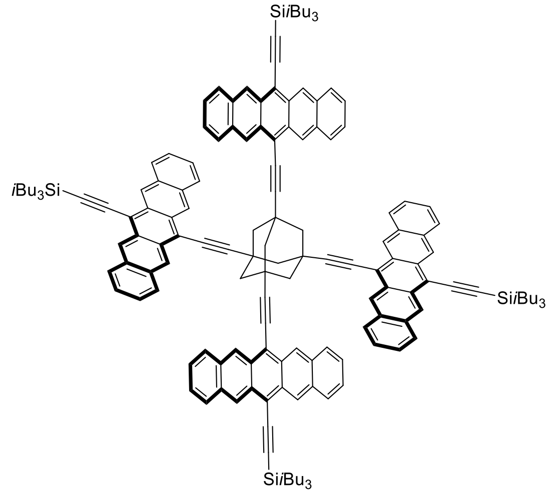

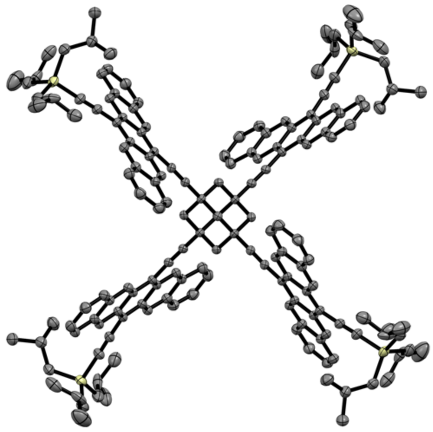

Compound PT: To a solution of **1** (0.050 g, 0.22 mmol) in dry, deoxygenated THF (20 mL) at –78 °C was added LiHMDS (1.1 mL, 1.1 mmol, 1 M in THF/ethylbenzene) and the mixture was stirred for 45 min at –78 °C, giving a solution of **4**.

A solution of **5** (0.720 g, 1.32 mmol) in dry, deoxygenated THF (15 mL) was added *via* cannula at –78 °C to the solution of **4** (as prepared above), and the resulting mixture was allowed to warm to rt and stirred for 18 h. Satd. aq. NH_4Cl was added (100 mL), and the aqueous phase extracted with CH_2Cl_2 (3 × 50 mL). The combined organic phases were washed with water (100 mL), brine (100 mL), dried ($MgSO_4$), filtered, and the solvent removed *in vacuo*. The band that corresponded at R_f = 0.75 was collected *via* column chromatography (silica, CH_2Cl_2) and subjected to reductive aromatization without further purification. To a solution of the crude intermediate (0.53 g, 0.22 mmol) in dry, deoxygenated THF (25 mL) was added $SnCl_2$•$2H_2O$ (0.586 g, 2.64 mmol) and 10% H_2SO_4 (0.5 mL) at rt. The flask was wrapped in aluminum foil in order to limit light exposure. The mixture was stirred for 6 h and then poured into MeOH (70 mL) and cooled to –78 °C. The suspension was filtered, and the residue was washed with MeOH (3 x 20 mL). The solid was dissolved in CH_2Cl_2 (30 mL), washed with water (100 mL), brine (100 mL), dried ($MgSO_4$), and the solvent removed by passing N_2 over the solution. Column chromatography (silica, CH_2Cl_2/hexanes 1:3) afforded **PT** (0.088 g, 18% based on **1**) as a deep blue solid. Mp 300–302 °C. R_f = 0.90 (CH_2Cl_2/hexanes 1:3). UV-vis (CH_2Cl_2) λ_{max} (ε): 271 (92500), 306 (731000), 326 (138000), 351 (31100), 439 (11800), 550 (16800), 592 (47700), 644 (101000). IR (ATR, solid state) 3046 (w), 2948 (m), 2864 (m), 2130 (m), 1461 (m) cm^{-1}. ^{1}H NMR (300 MHz, $CDCl_3$) δ 9.34 (s, 8H), 9.28 (s, 8H), 8.11 (d, *J* = 8.5 Hz, 8H), 7.95 (d, *J* = 8.6 Hz, 8H), 7.38 (t, *J* = 6.3 Hz, 8H), 7.29 (t, *J* = 8.0 Hz, 8H), 3.24 (s, 12H), 2.21 (nonet, *J* = 6.7 Hz, 12H), 1.20 (d, *J* = 6.6 Hz, 72H), 0.97 (d, *J* = 6.9 Hz, 24H). ^{13}C NMR (100 MHz, $CDCl_3$) δ 132.32, 132.27, 130.7, 130.4, 128.8, 128.5, 126.4, 126.10, 126.06, 125.9, 118.2, 118.0, 109.7, 109.4, 104.7, 80.1, 46.7, 33.3, 26.6, 25.47, 25.45. APPI HRMS calcd for $C_{162}H_{168}Si_4$ (M^+) 2225.2218, found 2225.2219. DSC: decomposition, 282 °C (onset), 386 °C (peak).

A crystal **PT** suitable for X-ray crystallography was grown by slow diffusion of MeOH into a solution of **PT** dissolved in THF at 5 °C for several days. X-ray crystallographic data for **PT** ($C_{162}H_{168}Si_4$), F_w = 2223.28; tetragonal crystal system, space group $I4_1/a$; crystal size = 0.24 × 0.16 × 0.12 mm^3; *a* = 32.6822(9) Å, *b* = 32.6822(9) Å, *c* = 12.5225(17) Å; *V* = 13376(2) $Å^3$; *Z* = 4; $\rho_{(calc)}$ = 1.104 g/cm^3; $2\theta_{max}$ = 134.73°; μ = 0.794 mm^{-1}; *T* = 120.00(10) K; total data collected = 7555; R_1 = 0.0981; ωR_2 = 0.2974 for 7555 data, 508 variables, and 140 restraints, largest difference, peak and hole = 0.69 and –0.23 $eÅ^{-3}$. Disorder in atoms C42/C43:C42a/C43a were refined with fixed 66:34% occupation, and C51/52/53:C51a/52a/53a with 62:38 occupation.

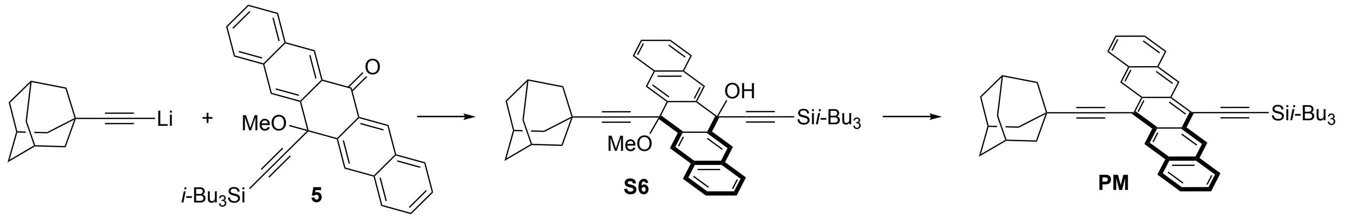

Compound **PM**: To 1-ethynyladamantane[17] (150 mg, 975 mmol) in dry THF (15 mL) at –78 °C was added LiHMDS (1.1 mL, 1.1 mmol, 1 M in THF/ethylbenzene), the mixture was stirred for 1 h at –78 °C and then used directly in the next step. A solution of **5** (641 mg, 1.17 mmol) in dry, deoxygenated THF (8 mL) was added *via* cannula at –78 °C to the lithium acetylide (as prepared above). The resulting mixture was allowed to warm to rt and stirred for 18 h. Satd. aq. NH_4Cl was added (100 mL), and the aqueous phase extracted with CH_2Cl_2 (3 × 75 mL). The combined organic phases were washed with water (150 mL), brine (150 mL), dried ($MgSO_4$), filtered, and the solvent removed *in vacuo*. The crude product was passed through a column (silica gel, hexane/EtOAc 3:1) to afford intermediate **S6** (193 mg, 28%) in sufficient purity to be carried on to the next step. To a solution of **S6** (100 mg, 0.141 mmol) in dry, deoxygenated THF (3 mL) was added $SnCl_2$•$2H_2O$ (66.9 mg, 0.296 mmol) and 10% H_2SO_4 (0.1 mL) at rt. The flask was wrapped in aluminum foil in order to limit light exposure. The mixture was stirred for 8 h and then poured into MeOH (70 mL). The suspension was filtered through a fritted funnel and the collected solid washed with MeOH (75 mL) to afford the product **PM** (49.0 mg, 53%) as a deep blue solid. Mp 182 °C. R_f = 0.80 (hexane/CH_2Cl_2 7:3). UV-vis (CH_2Cl_2) λ_{max} (ε): 271 (21200), 309 (245000), 325 (41100), 352 (8590), 438 (2740), 550 (4520), 590 (11900), 641 (22200) nm. IR (cast film CH_2Cl_2): 3049 (w), 2951 (s), 2905 (s), 2866 (m), 2851 (m), 2199 (w), 2122 (w) cm^{-1}. ^1H NMR (498 MHz, CD_2Cl_2): δ 9.27 (s, 2H), 9.21 (s, 2H), 8.08–8.04 (m, 2H), 8.01–7.97 (m, 2H), 7.45–7.42 (m, 4H), 2.37 (d, *J* = 2.8 Hz, 6H), 2.27–2.16 (m, 6H), 1.95–1.85 (m, 6H), 1.21 (d, *J* = 6.6 Hz, 18H), 1.00 (d, *J* = 7.0 Hz, 6H). ^{13}C NMR (126 MHz, $CDCl_3$): δ 132.4, 132.1, 130.9, 130.3, 128.9, 128.7, 126.4, 126.3, 126.1, 125.8, 119.6, 117.2, 114.4, 109.2, 105.0, 43.4, 36.7, 31.7, 28.4, 26.7, 25.63, 25.61 (one signal coincident or not observed). MALDI HRMS (DCTB) calcd for $C_{48}H_{54}Si$ (M^+) 658.3989, found 658.3988. DSC: Mp = 190 °C, decomposition, 191 °C (onset), 192 °C (peak).

Supplemental Results

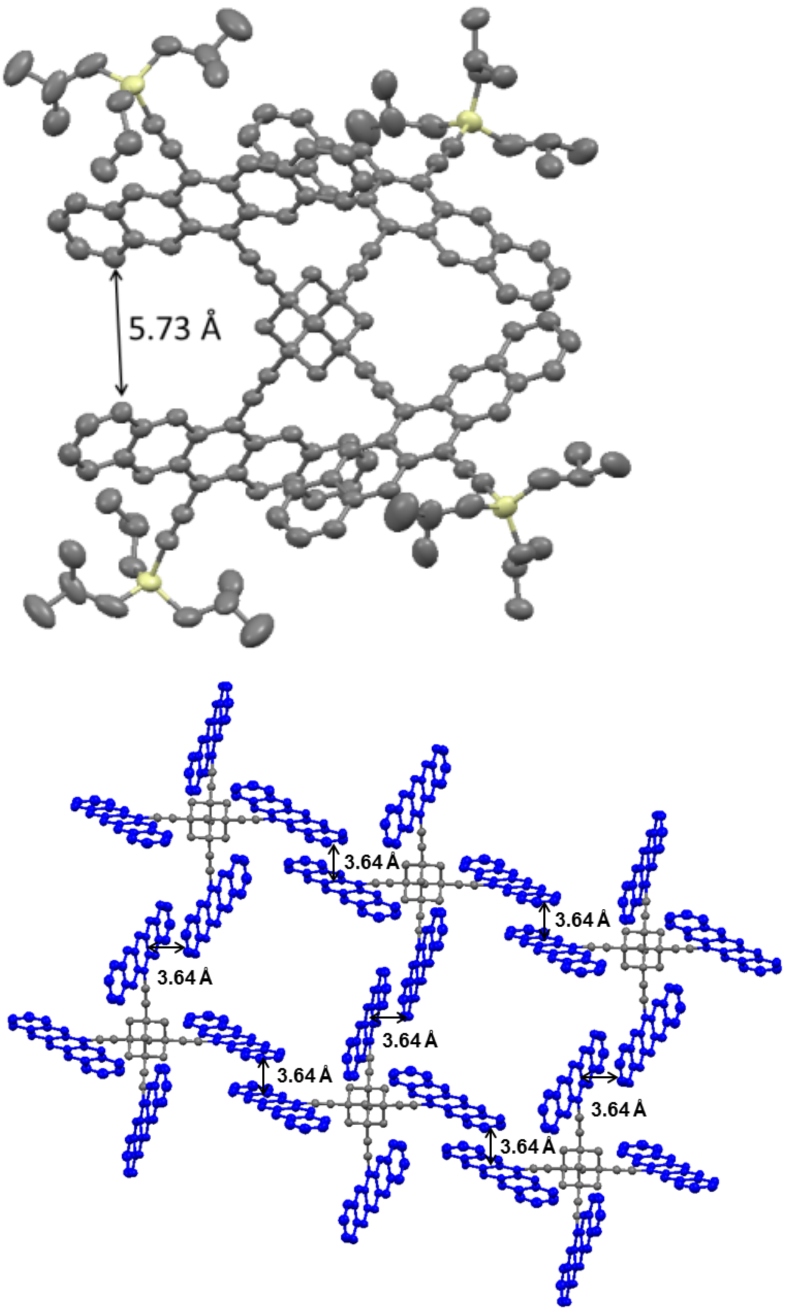

Figure S1. Top. Solid-state structure of pentacene **PT** showing the spatial arrangement of the four pentacene units in the tetrameric molecule and intramolecular distance of 5.73 Å between chromophores (hydrogen atoms have been omitted for clarity and ORTEPS drawn at 50% probability level). **Bottom**. Solid-state structure of **PT** showing intermolecular packing and interplanar distances (carbons of the acene planes are colored in blue, silylethynyl groups have been omitted for clarity); hydrogen atoms omitted for clarity and ORTEPs drawn at 50% probability level.

NMR Spectra

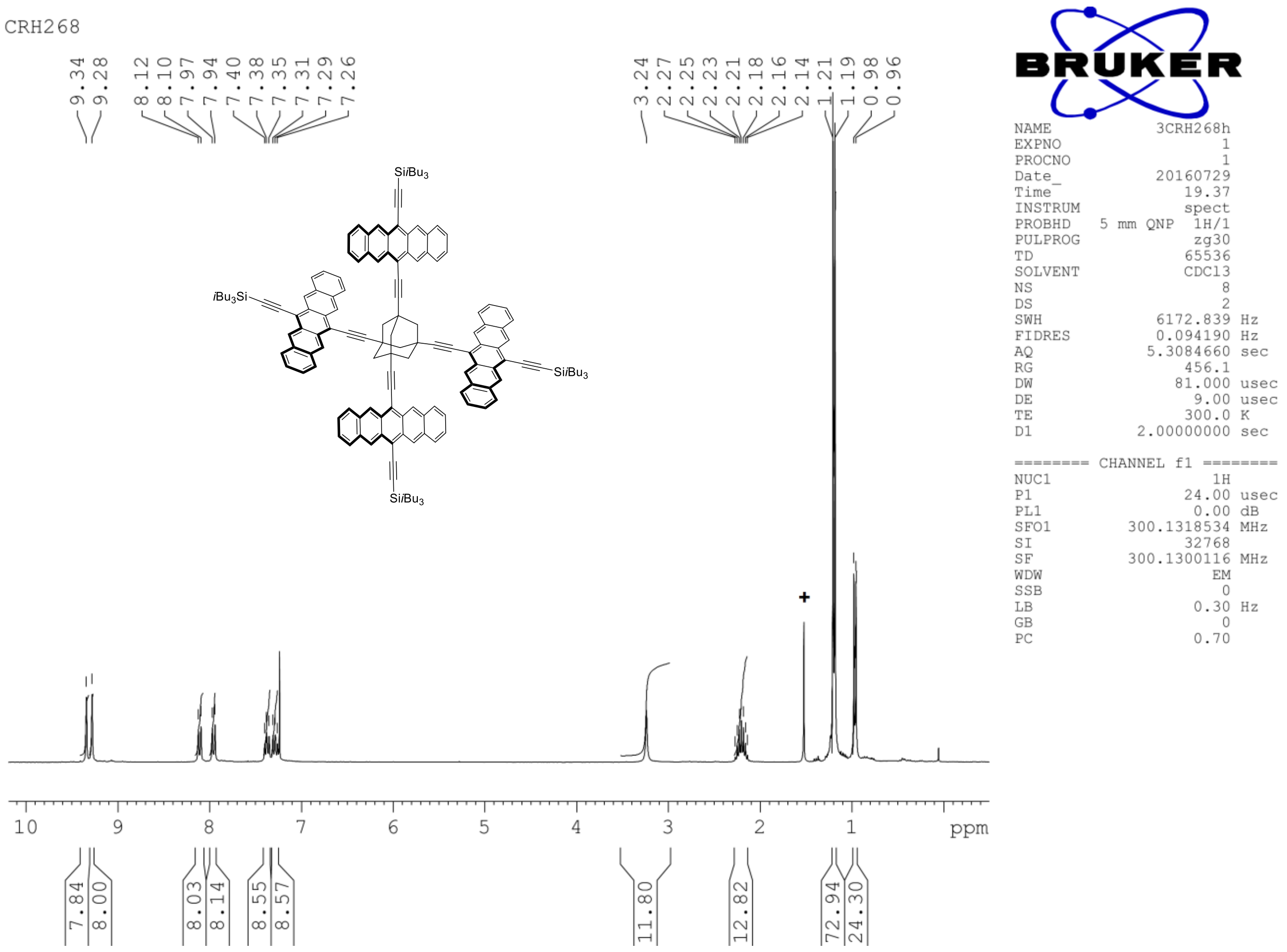

Figure S2. ^{1}H NMR spectrum of compound **PT** [300 MHz, $CDCl_3$, room temperature (rt), + = H_2O].

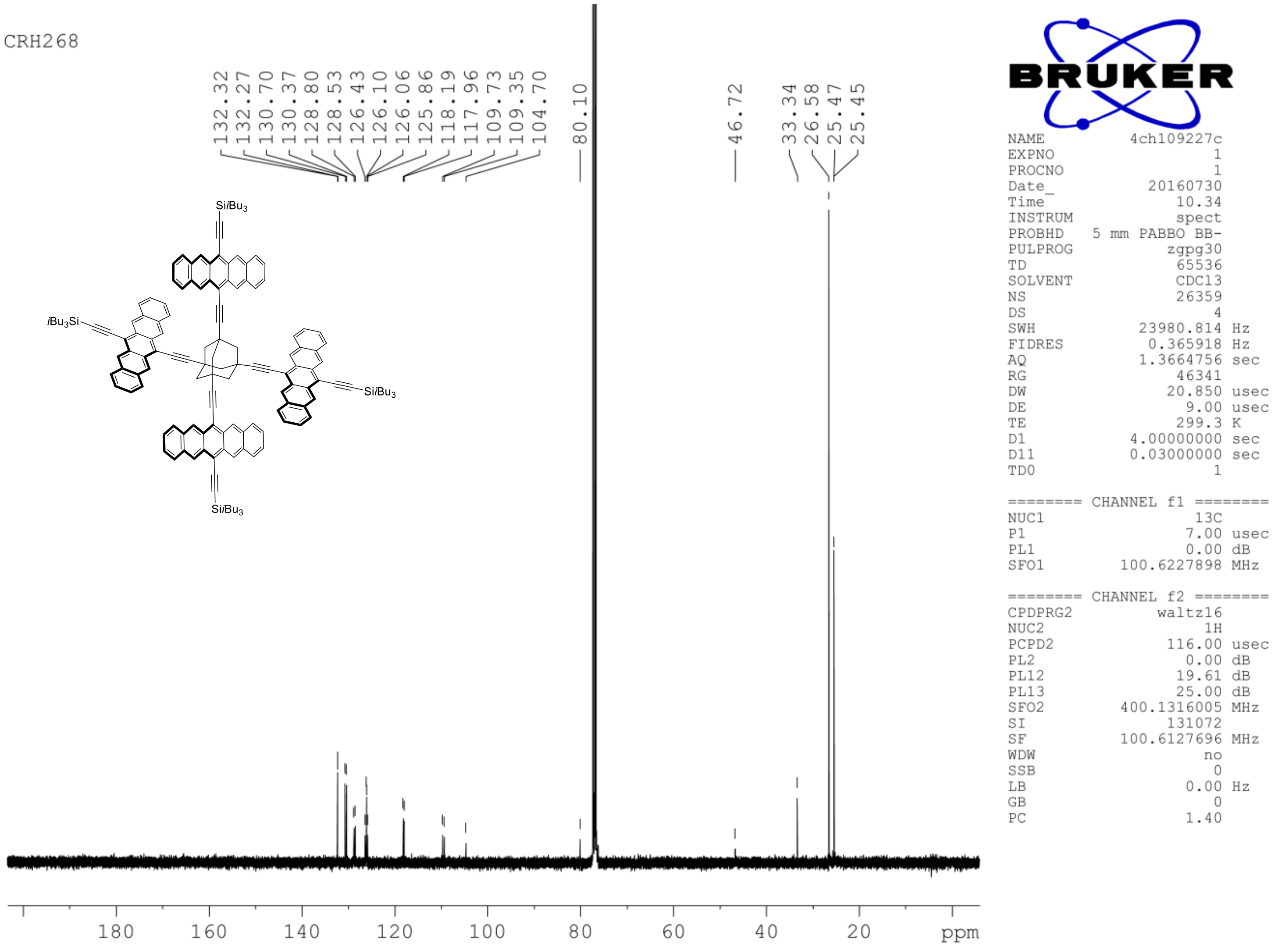

Figure S3. ^{13}C NMR spectrum of compound **PT** (100 MHz, $CDCl_3$, rt).

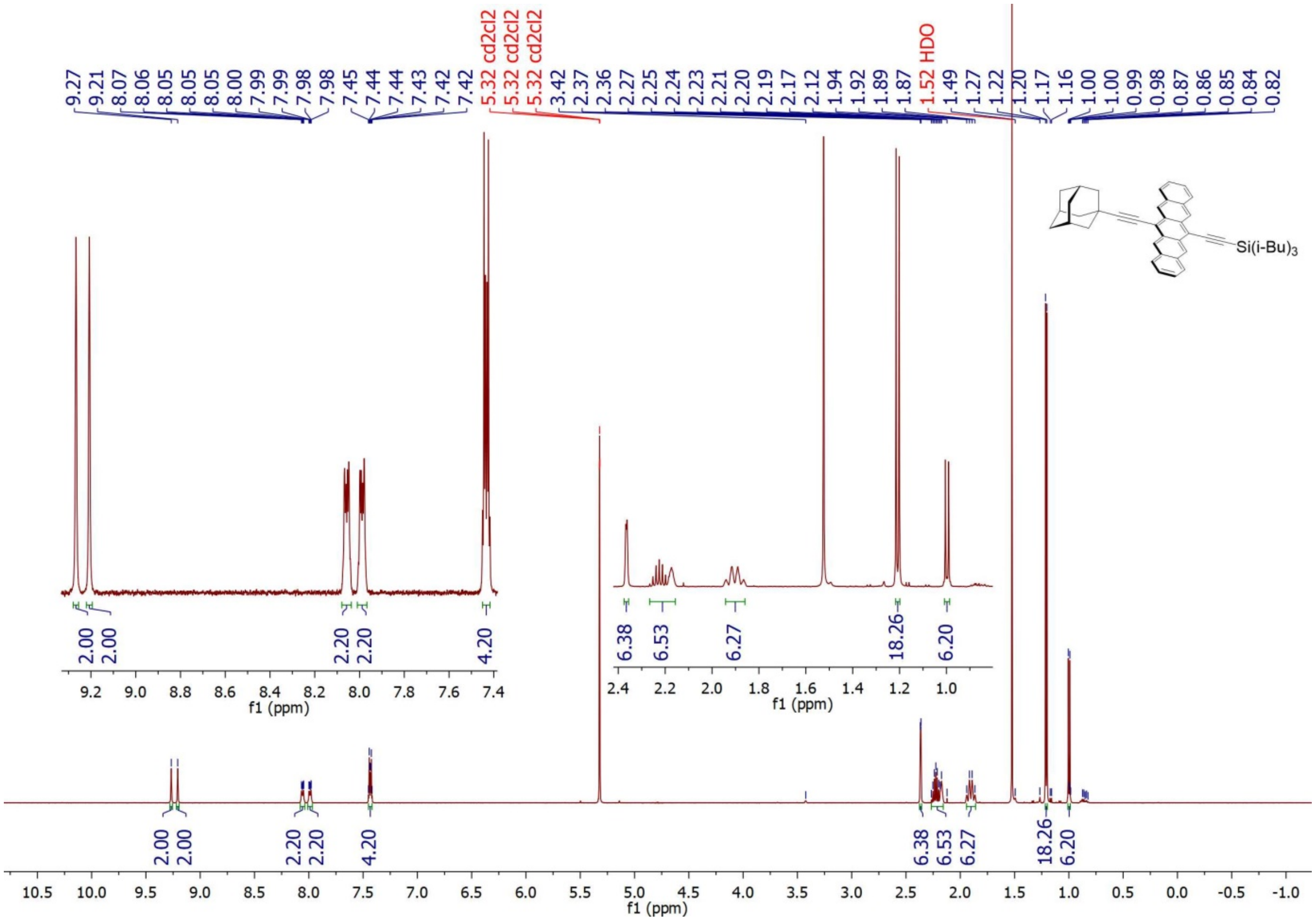

Figure S4. ^{1}H NMR spectrum of **PM** (498 MHz, CD_2Cl_2, rt).

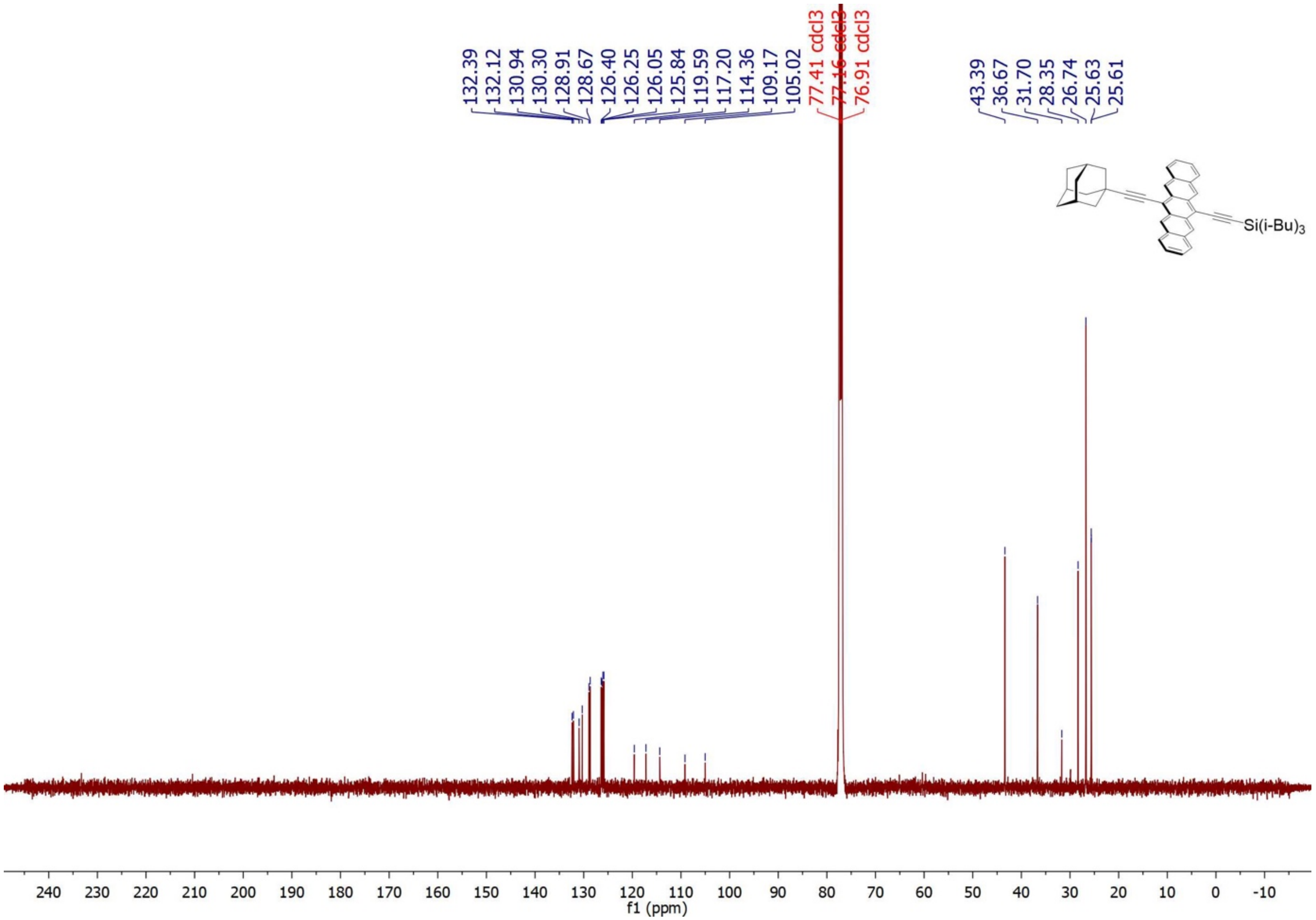

Figure S5. ^{13}C NMR spectrum of **PM** (126 MHz, CD_2Cl_2, rt).

Supplemental photophysical data

Steady-state characterization

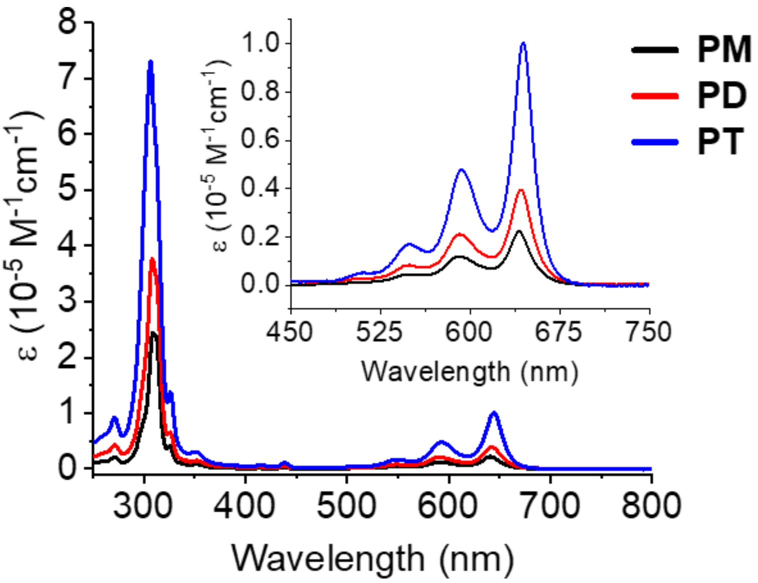

Figure S6. Steady-state absorption spectra of **PM**, **PD** and **PT**. Measured in CH_2Cl_2 at room temperature. The Inset show a zoom in on the $S_1 \leftarrow S_0$ absorptions.

Table S1. Extinction coefficients and full width at half maximum (FWHM) for the $S_1 \leftarrow S_0$ transition maximum of **PM**, **PD** and **PT** in CH_2Cl_2.

Molecule	λ_{Max}	ε_{Max}	FWHM
**PM**	641 nm	0.2219 x 10^5 $M^{-1}cm^{-1}$	23 nm / 70 meV
**PD**	642 nm	0.3955 x 10^5 $M^{-1}cm^{-1}$	23 nm / 69 meV
**PT**	644 nm	1.0051 x 10^5 $M^{-1}cm^{-1}$	20 nm / 60 meV

λ_{Max} is the wavelength of the maximum of the $S_1 \leftarrow S_0$ transition.

ε_{Max} is the extinction coefficient at the maximum of the $S_1 \leftarrow S_0$ transition.

The FWHM is given for the 0-*0 transition.

Table S2. Fluorescence quantum yields of **PM**, **PD** and **PT** in different solvents.[a]

Molecule	Fluorescence Quantum Yield…	
	… in benzonitrile	… in toluene
**PM**	9%	26%
**PD**	3%	2%
**PT**	1%	1%

[a] Measured relative to a zinc phthalocyanine used as a reference compound.[3]

Room temperature (RT) transient absorption (TA) spectroscopy

Table S3. Lifetimes of the transient states of **PM**, **PD** and **PT** in different solvents.

**Molecule**	**Solvent**	**$\tau(S_1)^b$**	---	---	**$\tau(T_1)^b$**
**PM**	benzonitrile	6.2 ns	---	---	50 µs
	toluene	13 ns	---	---	33 µs
**Molecule**	**Solvent**	**$\tau[(S_1S_0)]^a$**	**$\tau[^1(T_1T_1)]^b$**	**$\tau[^5(T_1T_1)]^b$**	**$\tau[^5(T_1+T_1)]^b$**
**PD**	benzonitrile	401 ps	8.3 ns	91 ns	32 µs
	toluene	805 ps	14 ns	84 ns	31 µs
**Molecule**	**Solvent**	**$\tau[(S_1S_0\ S_0S_0)]^a$**	**$\tau[^1(T_1T_1\ S_0S_0)]^b$**	**$\tau[^5(T_1T_1\ S_0S_0)]^b$**	**$\tau[^5(T_1+T_1+\ S_0+S_0)]^b$**
**PT**	benzonitrile	144 ps	12 ns	70 ns	32 µs
	toluene	292 ps	11 ns	71 ns	30 µs

[a] Lifetimes were extracted from fsTA measurements.

[b] Lifetimes were extracted from nsTA measurements.

See **Figures S7-S12** for the raw data and the global analyses.

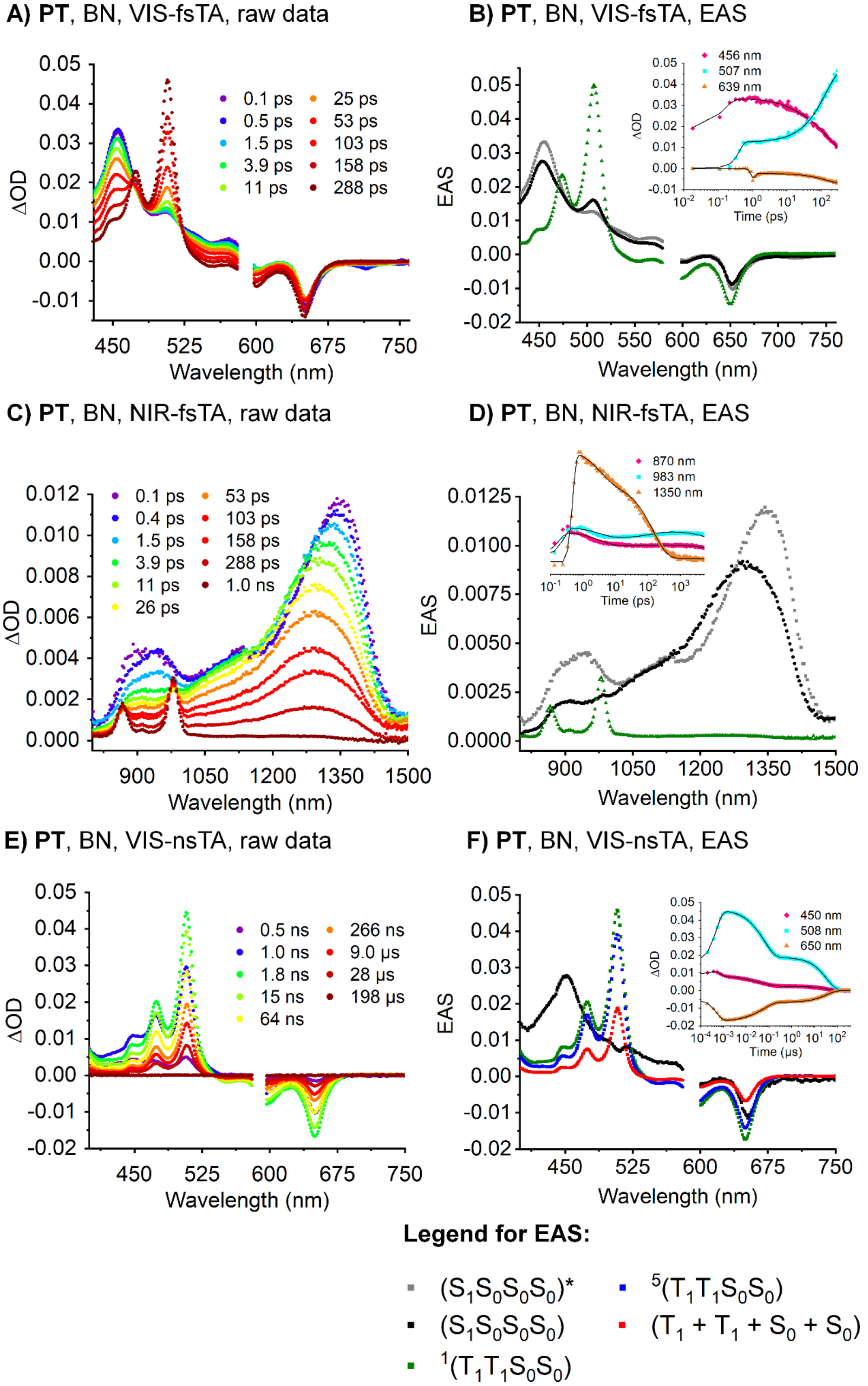

Figure S7. RT TA data of **PT** in argon-saturated benzonitrile (BN) and the corresponding global analysis data. Left) TA data obtained upon femto- and nanosecond pump-probe experiments (590 nm) of **PT** with several time delays (see legends). See figure captions for further details. Right) Deconvoluted EAS of the singlet state $(S_1S_0S_0S_0)^*$ state before solvent relaxation (grey), the $(S_1S_0S_0S_0)$ state after solvent relaxation (black), the correlated triplet pair state with singlet spin $^1(T_1T_1S_0S_0)$ (green), the correlated triplet pair state with quintet spin $^5(T_1T_1S_0S_0)$ (blue) and the decorrelated triplet state $(T_1 + T_1 + S_0 + S_0)$ (red) of **PT** as obtained by global analysis with a sequential model of the TA data shown left. See figure captions for further details. Insets: Raw data single-wavelength kinetics and fits to the data.

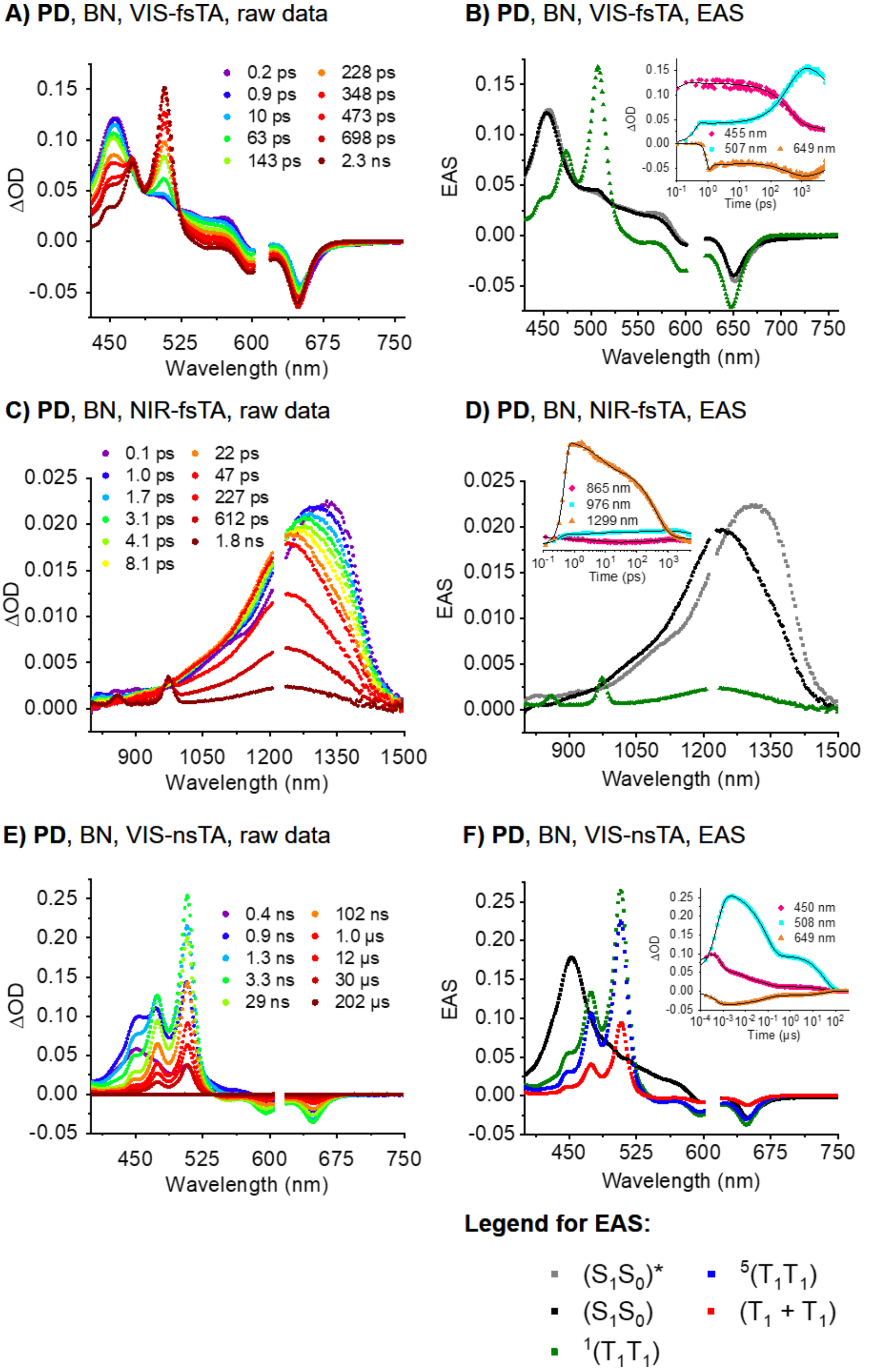

Figure S8. RT TA data of **PD** in argon-saturated benzonitrile (BN) and the corresponding global analysis data. Left) TA data obtained upon femto- and nanosecond pump-probe experiments (610 nm) of **PD** with several time delays (see legends). See figure captions for further details. Right) Deconvoluted EAS of the singlet state $(S_0S_1)^*$ state before solvent relaxation (grey), the (S_0S_1) state after solvent relaxation (black), the correlated triplet pair state with singlet spin $^1(T_1T_1)$ (green), the correlated triplet pair state with quintet spin $^5(T_1T_1)$ (blue) and the decorrelated triplet state $(T_1 + T_1)$ (red) of **PD** as obtained by global analysis with a sequential model of the TA data shown left. See figure captions for further details. Insets: Raw data single-wavelength kinetics and fits to the data.

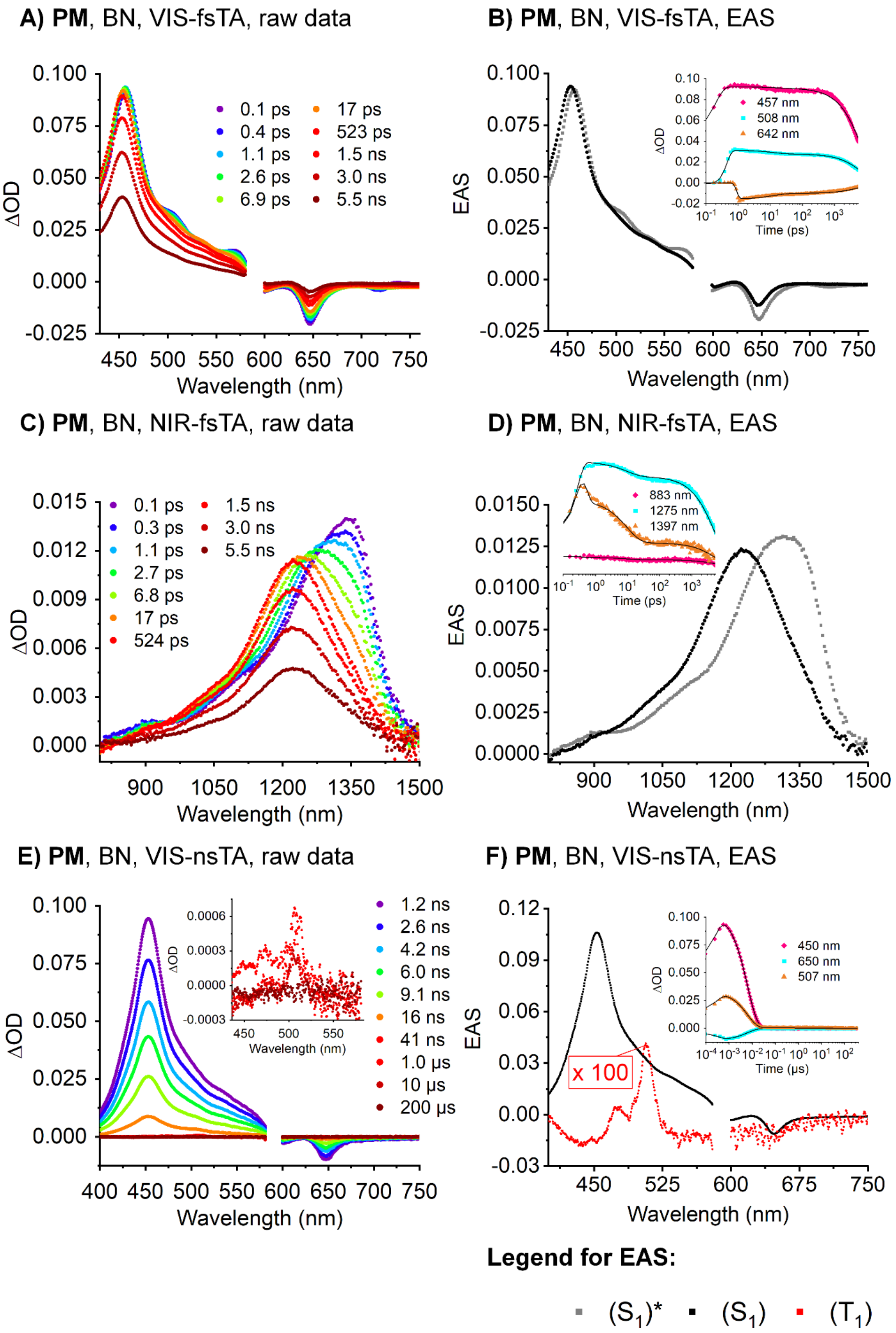

Figure S9. RT TA data of **PM** in argon-saturated benzonitrile (BN) and the corresponding global analysis data. Left) TA data obtained upon femto- and nanosecond pump-probe experiments (590 nm) of **PM** with several time delays (see legends). See figure captions for further details. The inset in **E)** shows a zoom-in on the triplet signal. Right) Deconvoluted EAS of the singlet state $(S_1)^*$ state before solvent relaxation (grey), the (S_1) state after solvent relaxation (black), and the triplet state (T_1) (red) of **PM** as obtained by global analysis with a sequential model of the TA data shown left. See figure captions for further details. Insets: Raw data single-wavelength kinetics and fits to the data.

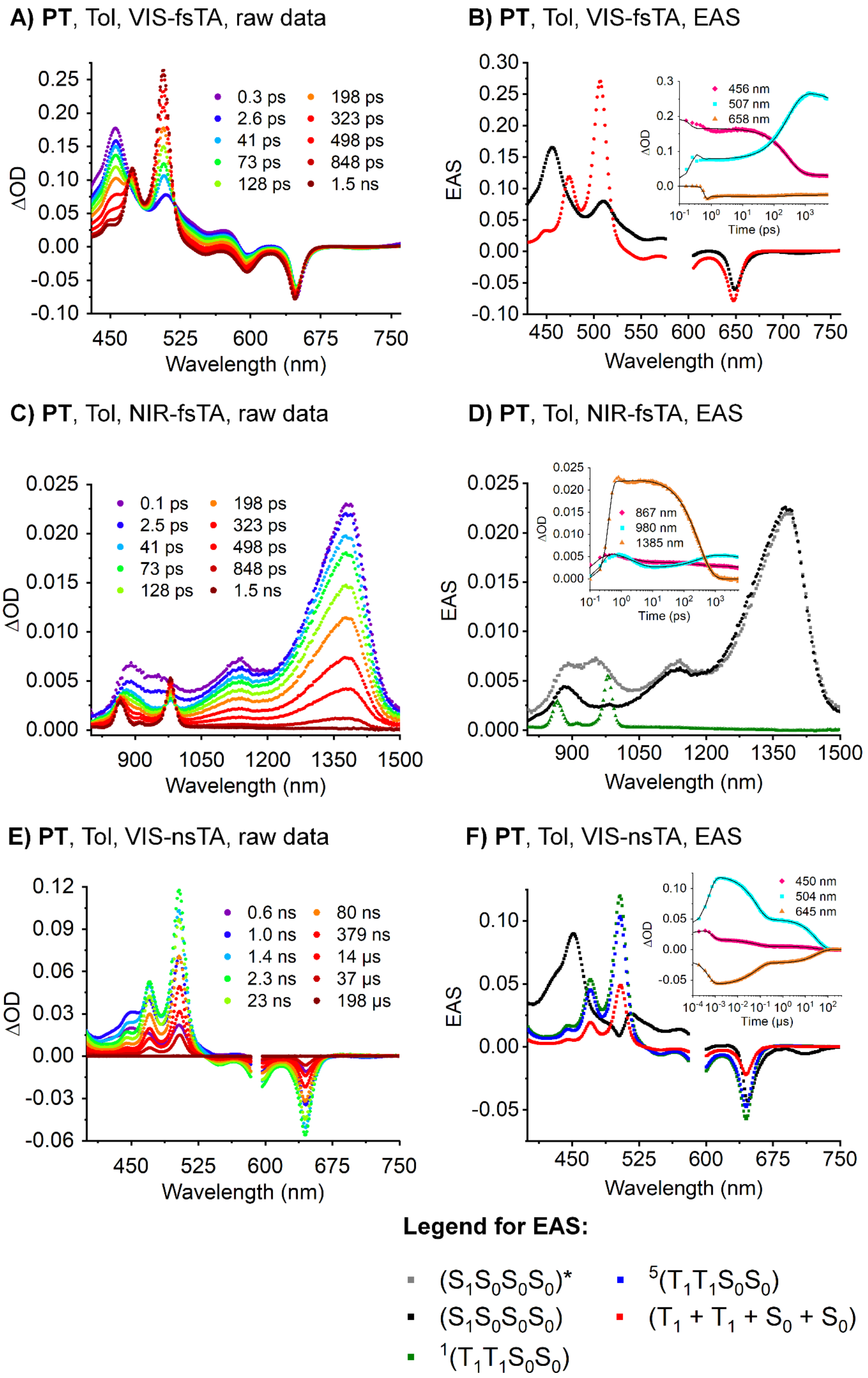

Figure S10. RT TA data of **PT** in argon-saturated toluene (Tol) and the corresponding global analysis data. Left) TA data obtained upon femto- and nanosecond pump-probe experiments (590 nm) of **PT** with several time delays (see legends). See figure captions for further details. Right) Deconvoluted EAS of the singlet state ($S_1S_0S_0S_0$)* state before solvent relaxation (grey), the ($S_1S_0S_0S_0$) state after solvent relaxation (black), the correlated triplet pair state with singlet spin $^1(T_1T_1S_0S_0)$ (green), the correlated triplet pair state with quintet spin $^5(T_1T_1S_0S_0)$ (blue) and the decorrelated triplet state ($T_1 + T_1 + S_0 + S_0$) (red) of **PT** as obtained by global analysis with a sequential model of the TA data shown left. See figure captions for further details. Insets: Raw data single-wavelength kinetics and fits to the data.

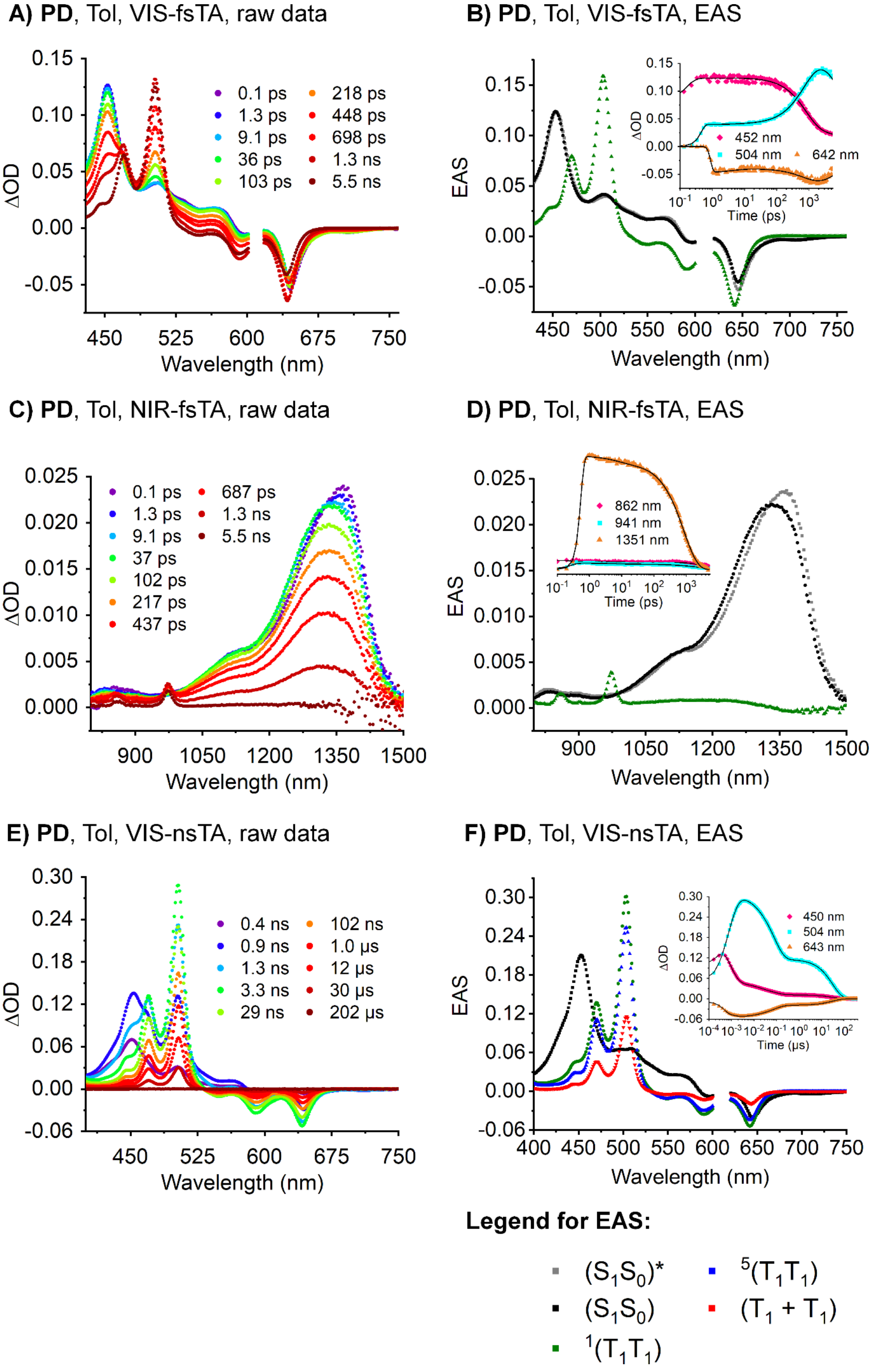

Figure S11. RT TA data of **PD** in argon-saturated toluene (Tol) and the corresponding global analysis data. Left) TA data obtained upon femto- and nanosecond pump-probe experiments (610 nm) of **PD** with several time delays (see legends). See figure captions for further details. Right) Deconvoluted EAS of the singlet state $(S_0S_1)^*$ state before solvent relaxation (grey), the (S_0S_1) state after solvent relaxation (black), the correlated triplet pair state with singlet spin $^1(T_1T_1)$ (green), the correlated triplet pair state with quintet spin $^5(T_1T_1)$ (blue) and the decorrelated triplet state $(T_1 + T_1)$ (red) of **PD** as obtained by global analysis with a sequential model of the TA data shown left. See figure captions for further details. Insets: Raw data single-wavelength kinetics and fits to the data.

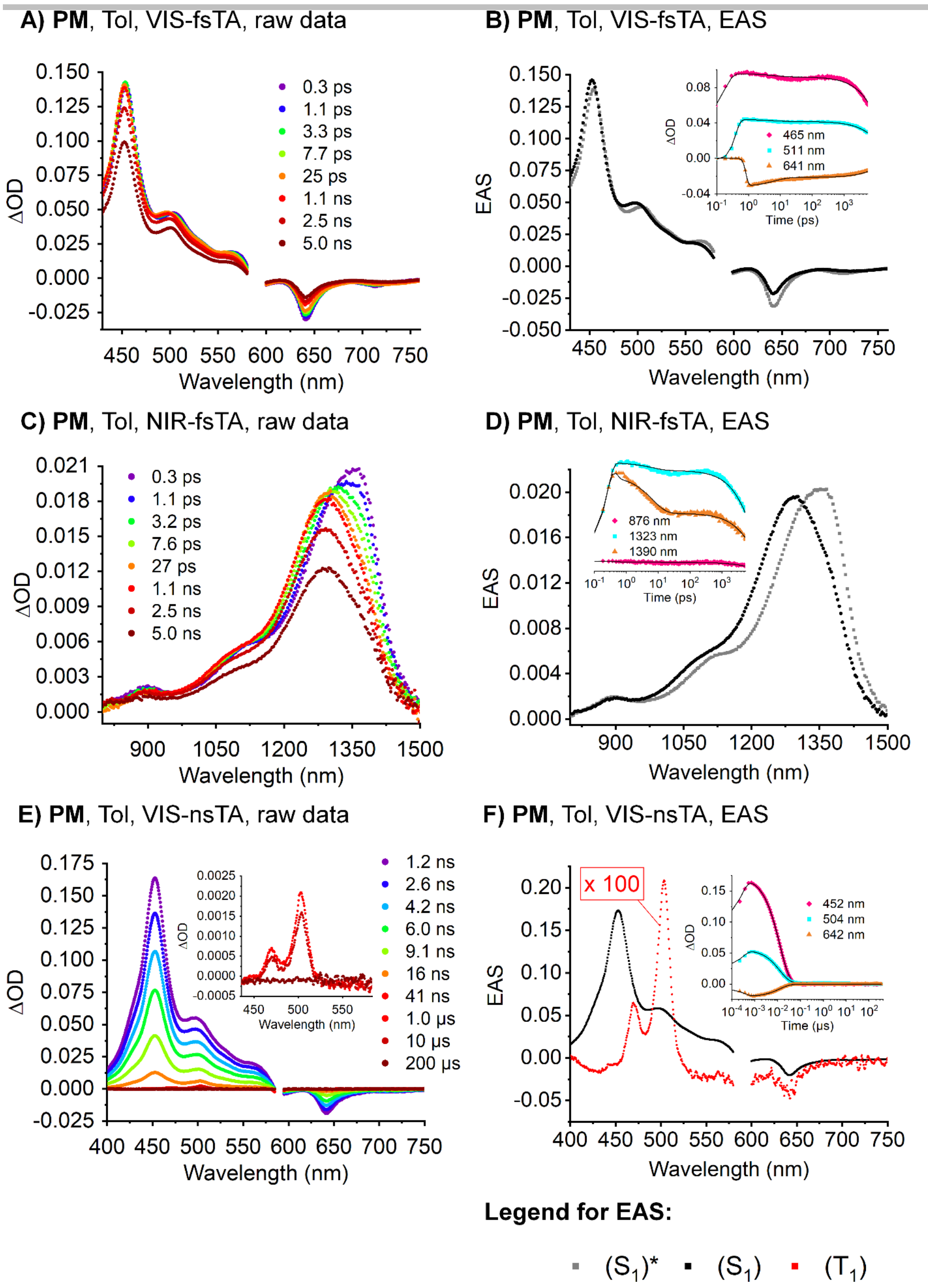

Figure S12. RT TA data of **PM** in argon-saturated toluene (Tol) and the corresponding global analysis data. Left) TA data obtained upon femto- and nanosecond pump-probe experiments (590 nm) of **PM** with several time delays (see legends). See figure captions for further details. The inset in **E)** shows a zoom-in on the triplet signal. Right) Deconvoluted EAS of the singlet state (S_1)* state before solvent relaxation (grey), the (S_1) state after solvent relaxation (black), and the triplet state (T_1) (red) of **PM** as obtained by global analysis with a sequential model of the TA data shown left. See figure captions for further details. Insets: Raw data single-wavelength kinetics and fits to the data.

Temperature dependent TA spectroscopy

Table S4. Lifetimes of the transient states of **PD** and **PT** in benzonitrile at various temperatures.[a]

**Molecule**	**T**	**τ[(S_1S_0)] or τ[($S_1S_0S_0S_0$)]**	**τ[^{1}(T_1T_1)] or τ[^{1}($T_1T_1S_0S_0$)]**	**τ[^{5}(T_1T_1)] or τ[^{5}($T_1T_1S_0S_0$)]**	**τ[(T_1+T_1)] or τ[(T_1+T_1+S_0+S_0)]**
**PD**	25 °C	0.41 ns	8.3 ns	87 ns	25 μs
	35 °C	0.38 ns	8.4 ns	86 ns	27 μs
	45 °C	0.36 ns	8.4 ns	84 ns	26 μs
	55 °C	0.32 ns	7.9 ns	82 ns	24 μs
	65 °C	0.31 ns	8.7 ns	82 ns	27μs
	75 °C	0.28 ns	8.7 ns	81 ns	25 μs
	85 °C	0.27 ns	9.2 ns	80 ns	27 μs
**PT**	25 °C	0.13 ns	12 ns	70 ns	32 μs
	35 °C	0.12 ns	10 ns	70 ns	31 μs
	45 °C	0.11 ns	10 ns	68 ns	32 μs
	55 °C	0.11 ns	12 ns	69 ns	32 μs
	65 °C	0.11 ns	12 ns	69 ns	31 μs
	75 °C	0.10 ns	10 ns	67 ns	31 μs
	85 °C	0.09 ns	10 ns	67 ns	29 μs

[a] All Lifetimes were extracted from nsTA measurements (τ[($S_1S_0S_0S_0$)] is too short for a meaningful extraction from nsTA data).

See **Figures S13-S19** for the raw data and the global analyses.

Table S5. Calculation of the triplet decorrelation rate of **PD** and **PT** in benzonitrile at various temperatures.

**Molecule**	**THF / Paraffin**	**k[^{5}(T$_1$T$_1$)] or k[^{5}(T$_1$T$_1$S$_0$S$_0$)]**[a]	**Decorrelation yield**[b]	**k$_{decorrelation}$**[c]
**PD**	25 °C	1.15×10^7 s^{-1}	40%	4.60×10^6 s^{-1}
	35 °C	1.16×10^7 s^{-1}	40%	4.64×10^6 s^{-1}
	45 °C	1.19×10^7 s^{-1}	40%	4.76×10^6 s^{-1}
	55 °C	1.22×10^7 s^{-1}	39%	4.76×10^6 s^{-1}
	65 °C	1.22×10^7 s^{-1}	40%	4.88×10^6 s^{-1}
	75 °C	1.23×10^7 s^{-1}	40%	4.92×10^6 s^{-1}
	85 °C	1.25×10^7 s^{-1}	40%	5.00×10^6 s^{-1}
**PT**	25 °C	1.43×10^7 s^{-1}	48%	6.86×10^6 s^{-1}
	35 °C	1.43×10^7 s^{-1}	47%	6.72×10^6 s^{-1}
	45 °C	1.47×10^7 s^{-1}	47%	6.91×10^6 s^{-1}
	55 °C	1.45×10^7 s^{-1}	48%	6.96×10^6 s^{-1}
	65 °C	1.45×10^7 s^{-1}	48%	6.96×10^6 s^{-1}
	75 °C	1.49×10^7 s^{-1}	45%	6.71×10^6 s^{-1}
	85 °C	1.49×10^7 s^{-1}	46%	6.86×10^6 s^{-1}

[a] k[^{5}(T$_1$T$_1$)] = 1 / τ[^{5}(T$_1$T$_1$)] / k[^{5}(T$_1$T$_1$S$_0$S$_0$)] = 1 / τ[^{5}(T$_1$T$_1$S$_0$S$_0$)].

[b] The decorrelation yield is defined as the intensity ratio of ^{5}(T$_1$T$_1$) / ^{5}(T$_1$T$_1$S$_0$S$_0$) and (T$_1$+T$_1$) / (T$_1$+T$_1$+S$_0$+S$_0$) in the global analysis of the corresponding TA data.

[c] The decorrelation rate, k[^{5}(T$_1$T$_1$)→(T$_1$+T$_1$)] / k[^{5}(T$_1$T$_1$S$_0$S$_0$)→(T$_1$+T$_1$+S$_0$+S$_0$)] is calculated as the product of the decorrelation yield and total deactivation rate of ^{5}(T$_1$T$_1$) / ^{5}(T$_1$T$_1$S$_0$S$_0$), k[^{5}(T$_1$T$_1$)]/ k[^{5}(T$_1$T$_1$S$_0$S$_0$)].

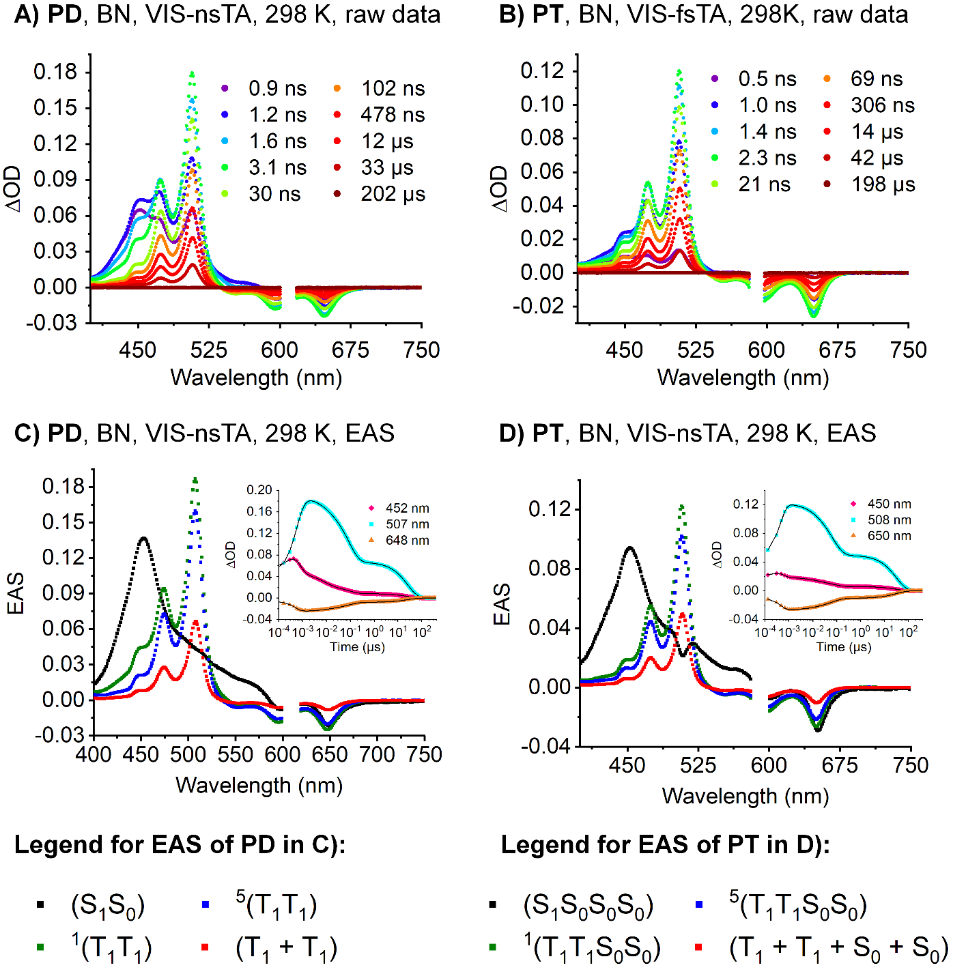

Figure S13. TA data of **PD** and **PT** in argon-saturated benzonitrile (BN) measured at 25 °C (298 K) and the corresponding global analysis data.
A&B) TA data obtained upon nanosecond pump-probe experiments (610 nm / 590 nm) of **PD / PT** (A / B) with several time delays (see legends). C&D) Deconvoluted EAS of the singlet excited state (S_1S_0) / ($S_1S_0S_0S_0$) state after solvent relaxation (black), the correlated triplet pair state with singlet spin $^1(T_1T_1)$ / $^1(T_1T_1S_0S_0)$ (green), the correlated triplet pair state with quintet spin $^5(T_1T_1)$ / $^5(T_1T_1S_0S_0)$ (blue) and the decorrelated triplet state (T_1+T_1) / (T_1+T_1+S_0+S_0) (red) of **PD** / **PT** (C / D) as obtained by global analysis with a sequential model of the TA data shown above. Insets: Raw data single-wavelength kinetics and fits to the data.

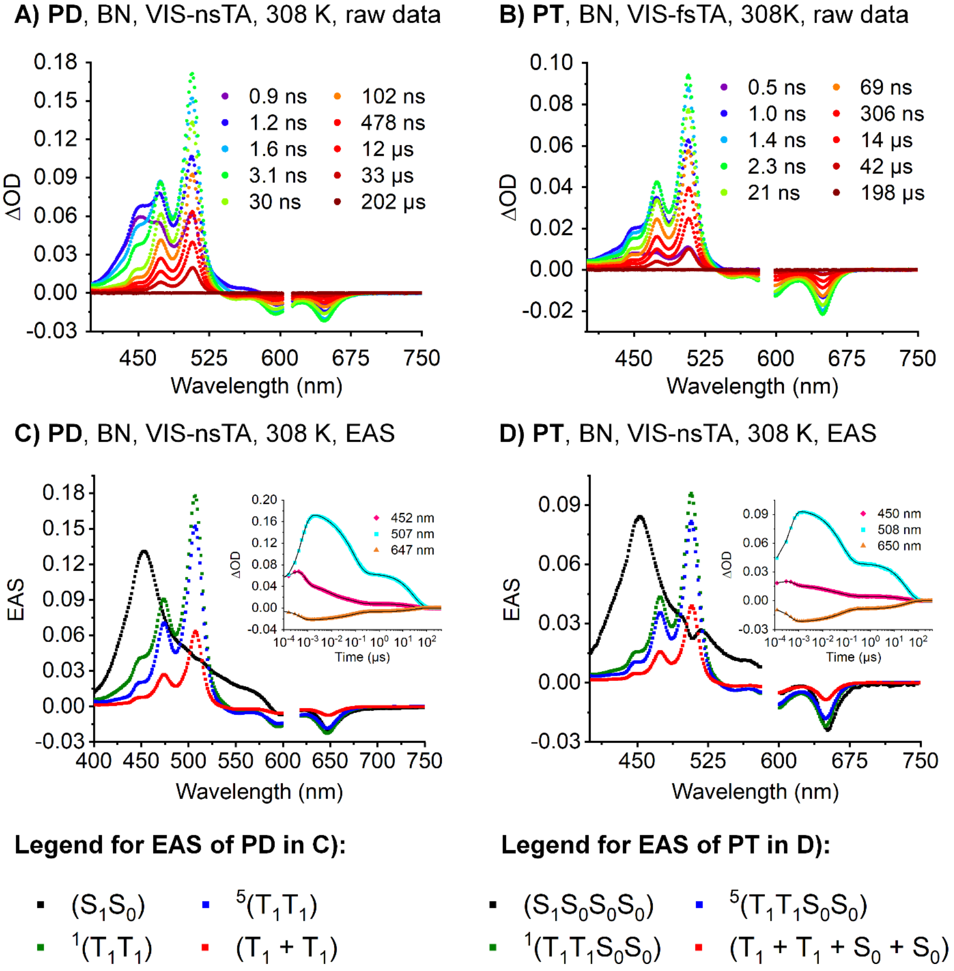

Figure S14. TA data of **PD** and **PT** in argon-saturated benzonitrile (BN) measured at 35 °C (308 K) and the corresponding global analysis data**.**
A&B) TA data obtained upon nanosecond pump-probe experiments (610 nm / 590 nm) of **PD / PT** (A / B) with several time delays (see legends). C&D) Deconvoluted EAS of the singlet excited state (S_1S_0) / ($S_1S_0S_0S_0$) state after solvent relaxation (black), the correlated triplet pair state with singlet spin $^1(T_1T_1)$ / $^1(T_1T_1S_0S_0)$ (green), the correlated triplet pair state with quintet spin $^5(T_1T_1)$ / $^5(T_1T_1S_0S_0)$ (blue) and the decorrelated triplet state (T_1+T_1) / (T_1+T_1+S_0+S_0) (red) of **PD** / **PT** (C / D) as obtained by global analysis with a sequential model of the TA data shown above. Insets: Raw data single-wavelength kinetics and fits to the data.

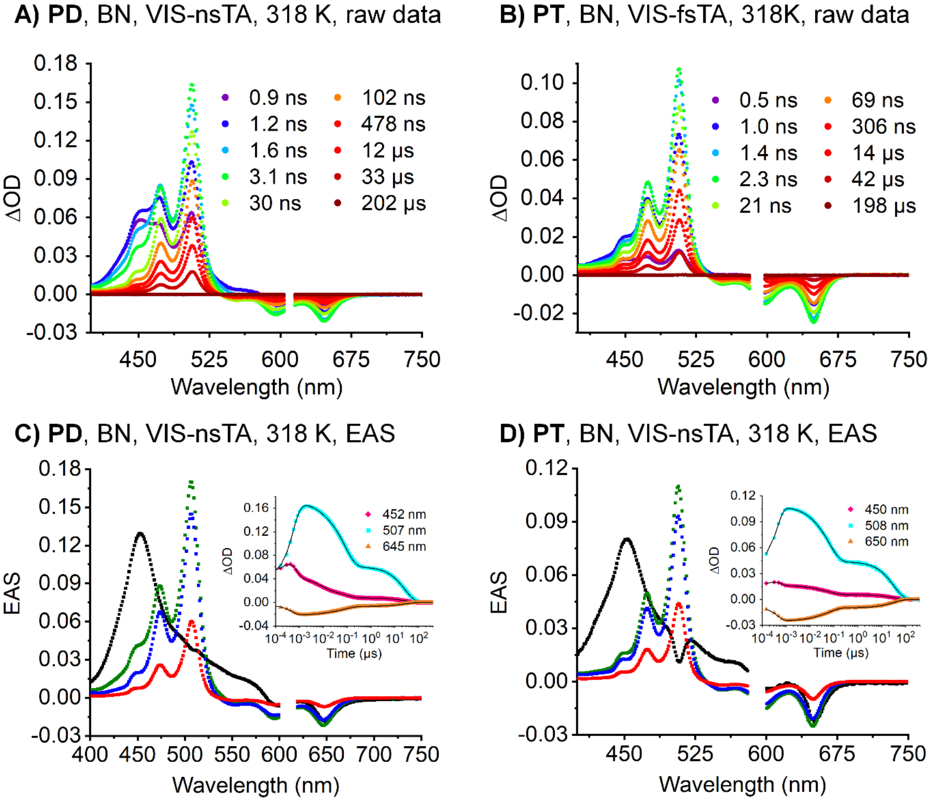

Legend for EAS of PD in C):

- (S_1S_0)
- $^5(T_1T_1)$
- $^1(T_1T_1)$
- $(T_1 + T_1)$

Legend for EAS of PT in D):

- $(S_1S_0S_0S_0)$
- $^5(T_1T_1S_0S_0)$
- $^1(T_1T_1S_0S_0)$
- $(T_1 + T_1 + S_0 + S_0)$

Figure S15. TA data of **PD** and **PT** in argon-saturated benzonitrile (BN) measured at 45 °C (318 K) and the corresponding global analysis data.
A&B) TA data obtained upon nanosecond pump-probe experiments (610 nm / 590 nm) of **PD / PT** (A / B) with several time delays (see legends). C&D) Deconvoluted EAS of the singlet excited state (S_1S_0) / $(S_1S_0S_0S_0)$ state after solvent relaxation (black), the correlated triplet pair state with singlet spin $^1(T_1T_1)$ / $^1(T_1T_1S_0S_0)$ (green), the correlated triplet pair state with quintet spin $^5(T_1T_1)$ / $^5(T_1T_1S_0S_0)$ (blue) and the decorrelated triplet state (T_1+T_1) / $(T_1+T_1+S_0+S_0)$ of **PD** / **PT** (C / D) as obtained by global analysis with a sequential model of the TA data shown above. Insets: Raw data single-wavelength kinetics and fits to the data.

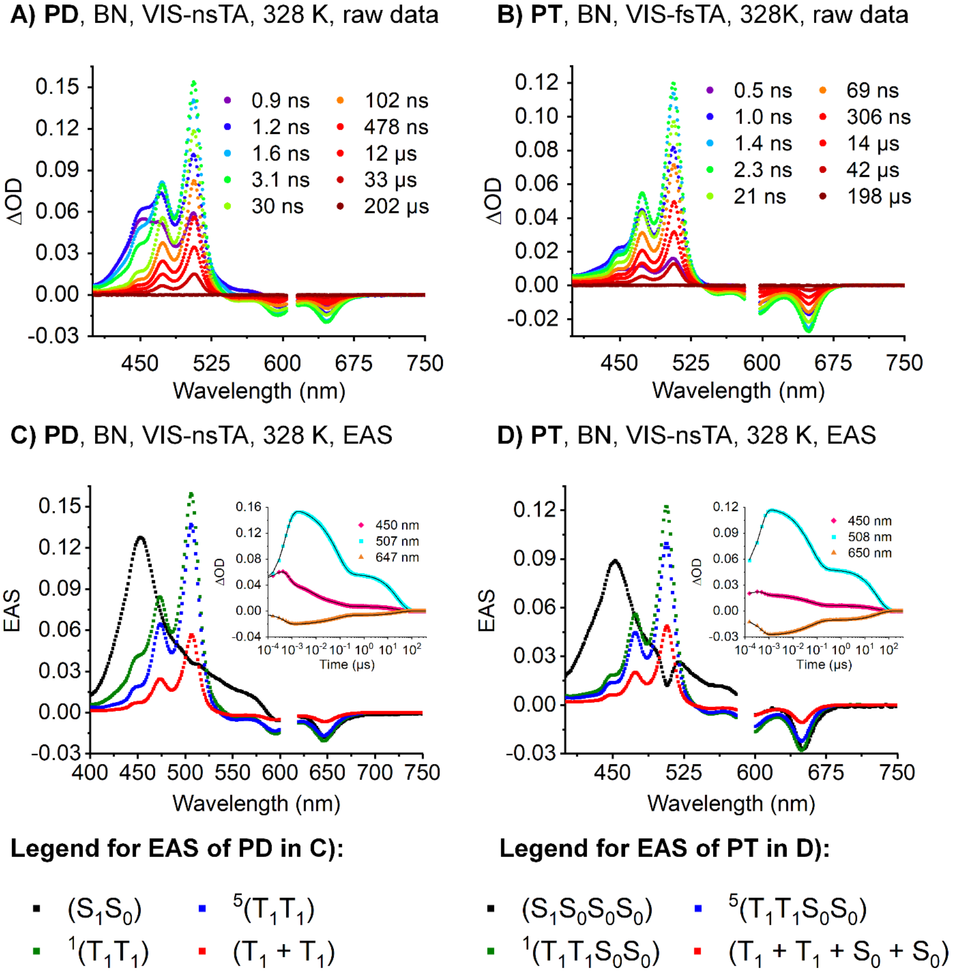

Figure S16. TA data of **PD** and **PT** in argon-saturated benzonitrile (BN) measured at 55 °C (328 K) and the corresponding global analysis data.
A&B) TA data obtained upon nanosecond pump-probe experiments (610 nm / 590 nm) of **PD / PT** (A / B) with several time delays (see legends). C&D) Deconvoluted EAS of the singlet excited state (S_1S_0) / ($S_1S_0S_0S_0$) state after solvent relaxation (black), the correlated triplet pair state with singlet spin $^1(T_1T_1)$ / $^1(T_1T_1S_0S_0)$ (green), the correlated triplet pair state with quintet spin $^5(T_1T_1)$ / $^5(T_1T_1S_0S_0)$ (blue) and the decorrelated triplet state (T_1+T_1) / (T_1+T_1+S_0+S_0) (red) of **PD** / **PT** (C / D) as obtained by global analysis with a sequential model of the TA data shown above. Insets: Raw data single-wavelength kinetics and fits to the data.

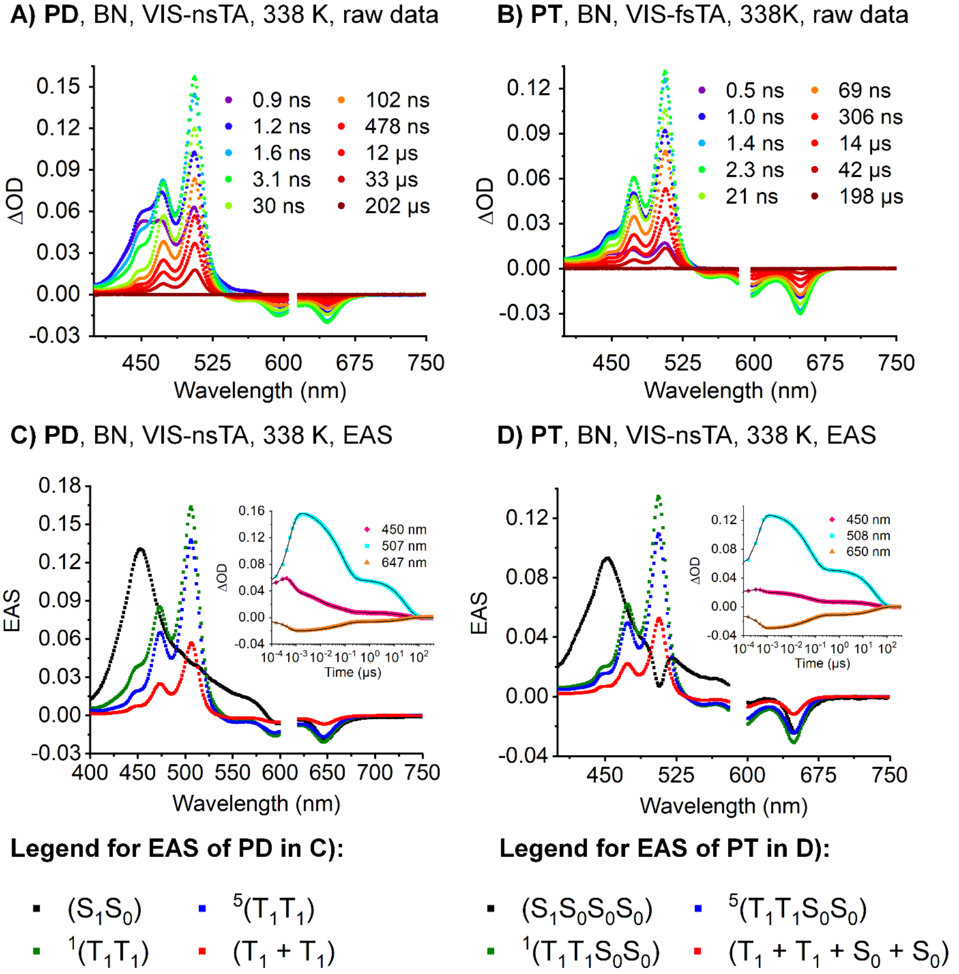

Figure S17. TA data of **PD** and **PT** in argon-saturated benzonitrile (BN) measured at 65 °C (338 K) and the corresponding global analysis data.
A&B) TA data obtained upon nanosecond pump-probe experiments (610 nm / 590 nm) of **PD / PT** (A / B) with several time delays (see legends). C&D) Deconvoluted EAS of the singlet excited state (S_1S_0) / ($S_1S_0S_0S_0$) state after solvent relaxation (black), the correlated triplet pair state with singlet spin $^1(T_1T_1)$ / $^1(T_1T_1S_0S_0)$ (green), the correlated triplet pair state with quintet spin $^5(T_1T_1)$ / $^5(T_1T_1S_0S_0)$ (blue) and the decorrelated triplet state (T_1+T_1) / (T_1+T_1+S_0+S_0) (red) of **PD** / **PT** (C / D) as obtained by global analysis with a sequential model of the TA data shown above. Insets: Raw data single-wavelength kinetics and fits to the data.

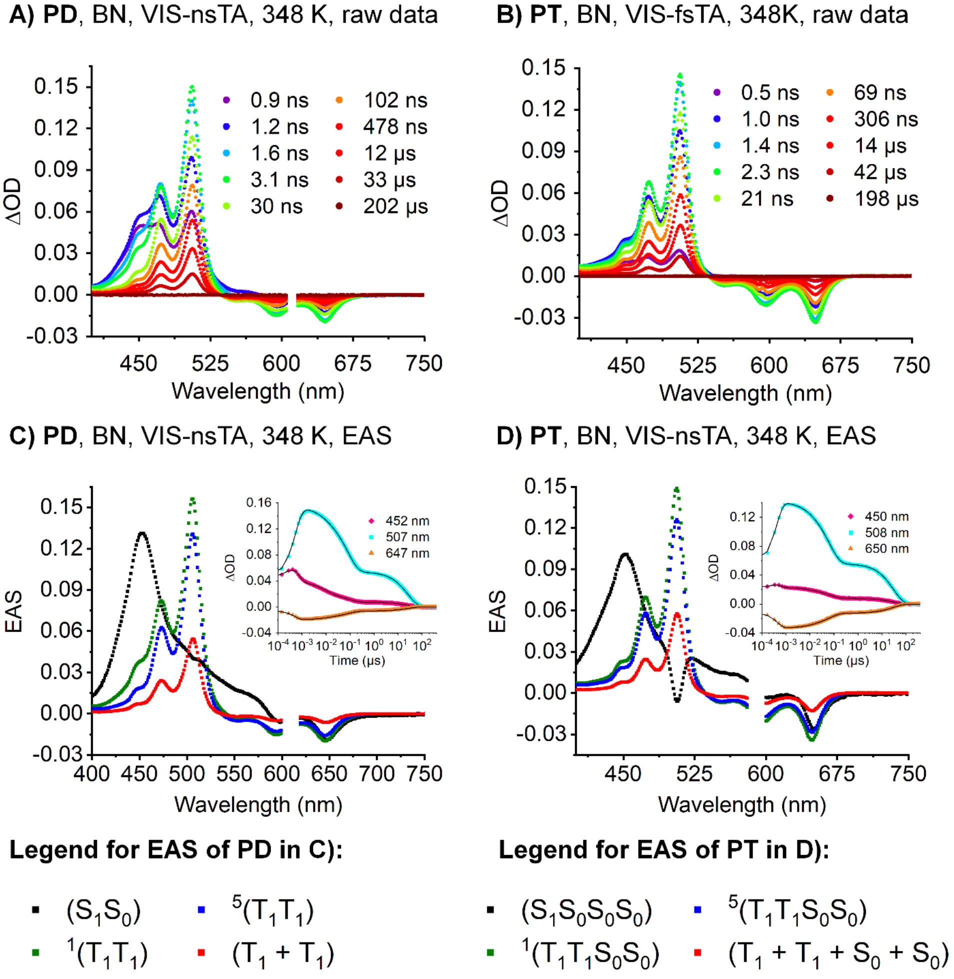

Figure S18. TA data of **PD** and **PT** in argon-saturated benzonitrile (BN) measured at 75 °C (348 K) and the corresponding global analysis data. A&B) TA data obtained upon nanosecond pump-probe experiments (610 nm / 590 nm) of **PD / PT** (A/B) with several time delays (see legends). C&D) Deconvoluted EAS of the singlet excited state (S_1S_0) / ($S_1S_0S_0S_0$) state after solvent relaxation (black), the correlated triplet pair state with singlet spin $^1(T_1T_1)$ / $^1(T_1T_1S_0S_0)$ (green), the correlated triplet pair state with quintet spin $^5(T_1T_1)$ / $^5(T_1T_1S_0S_0)$ (blue) and the decorrelated triplet state (T_1+T_1) / (T_1+T_1+S_0+S_0) (red) of **PD** / **PT** (C / D) as obtained by global analysis with a sequential model of the TA data shown above. Insets: Raw data single-wavelength kinetics and fits to the data.

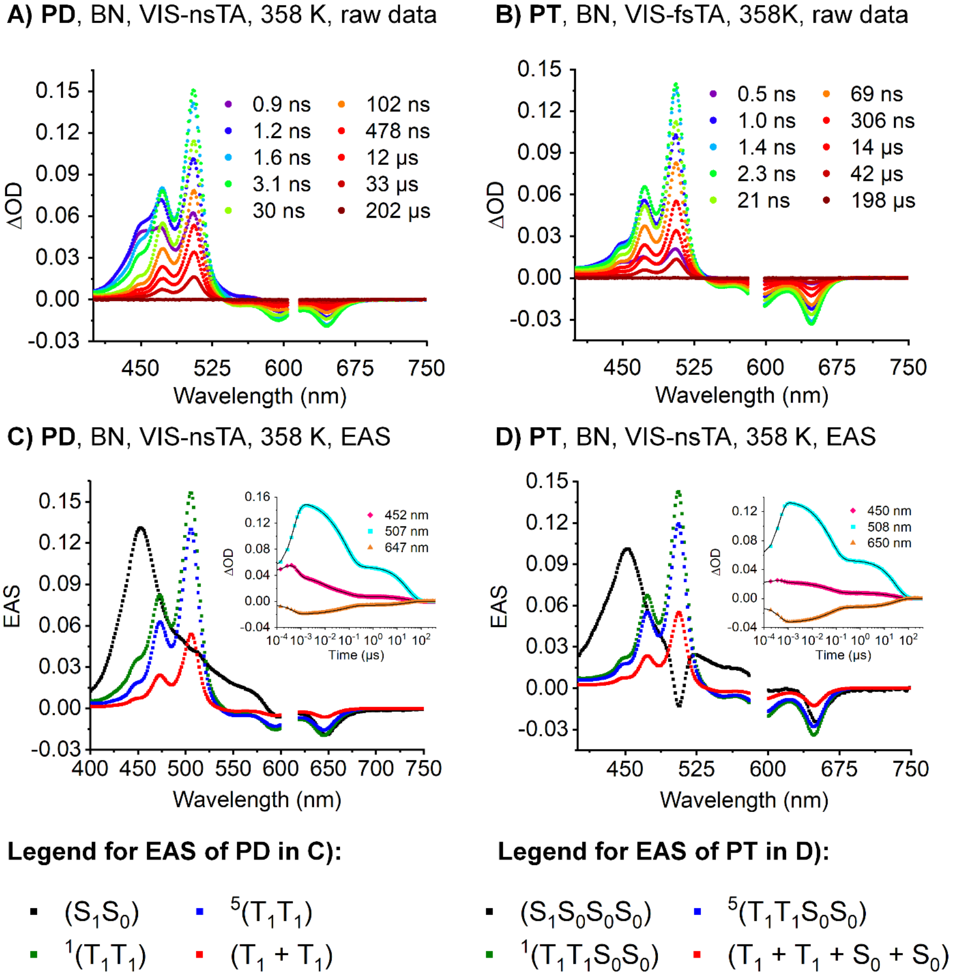

Figure S19. TA data of **PD** and **PT** in argon-saturated benzonitrile (BN) measured at 85 °C (358 K) and the corresponding global analysis data.
A&B) TA data obtained upon nanosecond pump-probe experiments (610 nm / 590 nm) of **PD / PT** (A / B) with several time delays (see legends). C&D) Deconvoluted EAS of the singlet excited state (S_1S_0) / ($S_1S_0S_0S_0$) state after solvent relaxation (black), the correlated triplet pair state with singlet spin $^1(T_1T_1)$ / $^1(T_1T_1S_0S_0)$ (green), the correlated triplet pair state with quintet spin $^5(T_1T_1)$ / $^5(T_1T_1S_0S_0)$ (blue) and the decorrelated triplet state (T_1+T_1) / (T_1+T_1+S_0+S_0) (red) of **PD** / **PT** (C / D) as obtained by global analysis with a sequential model of the TA data shown above. Insets: Raw data single-wavelength kinetics and fits to the data.

Electron transfer studies

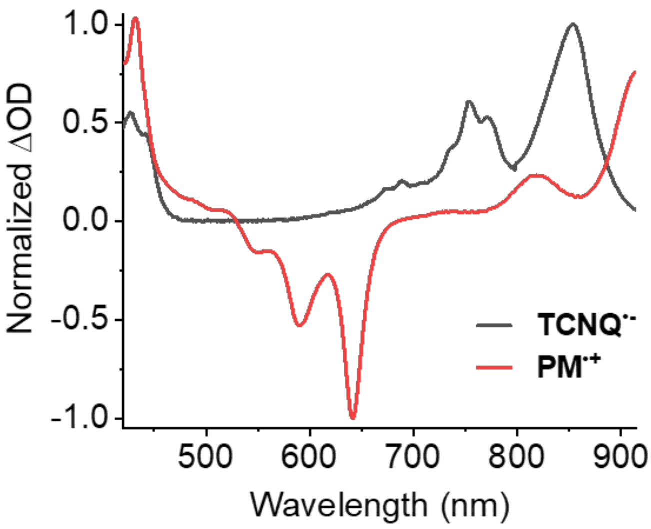

Figure S20. Normalized differential absorption changes obtained upon one electron oxidation of **PM** in argon-saturated DCM (grey) and one electron reduction of **TCNQ** in argon-saturated benzonitrile (red).

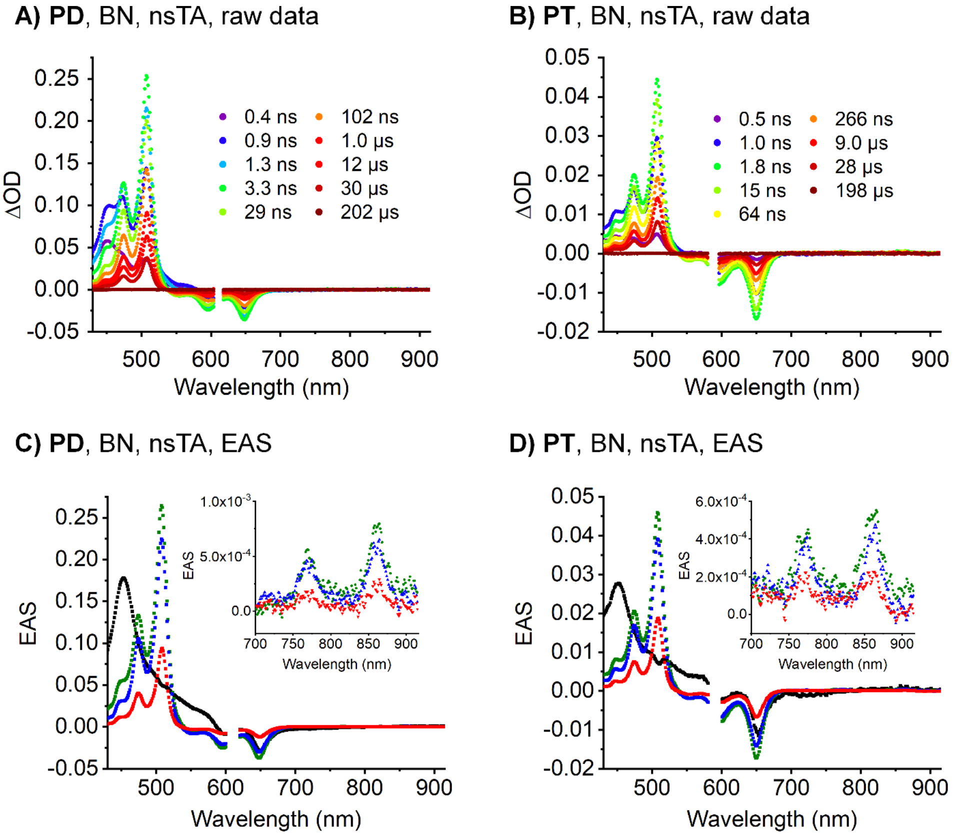

Legend for EAS of PD in C):

- (S_1S_0)
- $^1(T_1T_1)$
- $^5(T_1T_1)$
- (T_1+T_1)

Legend for EAS of PT in D):

- $(S_1S_0S_0S_0)$
- $^1(T_1T_1S_0S_0)$
- $^5(T_1T_1S_0S_0)$
- $(T_1+T_1+S_0+S_0)$

Figure S21. RT nsTA data of **PD** and **PT** in argon-saturated benzonitrile (BN) and the corresponding global analysis data from 430 to 915 nm for comparison with the nsTA data of **PD** and **PT** upon addition of **TCNQ**. A&B) TA data obtained upon nanosecond pump-probe experiments (610 nm / 590 nm) of **PD / PT** (A / B) with several time delays (see legends). C&D) Deconvoluted EAS of the singlet excited state (S_1S_0) / $(S_1S_0S_0S_0)$ state after solvent relaxation (black), the correlated triplet pair state with singlet spin $^1(T_1T_1)$ / $^1(T_1T_1S_0S_0)$ (green), the correlated triplet pair state with quintet spin $^5(T_1T_1)$ / $^5(T_1T_1S_0S_0)$ (blue), the decorrelated triplet state (T_1+T_1) / $(T_1+T_1+S_0+S_0)$ (red) of **PD** / **PT** (C / D) upon addition of TCNQ as obtained by global analysis with a sequential model of the TA data shown above. Insets: Zoom-in on the triplet features between 700 and 915 nm. See **Figures S7F and S8F** for the raw data single-wavelength kinetics and fits to the data.

.

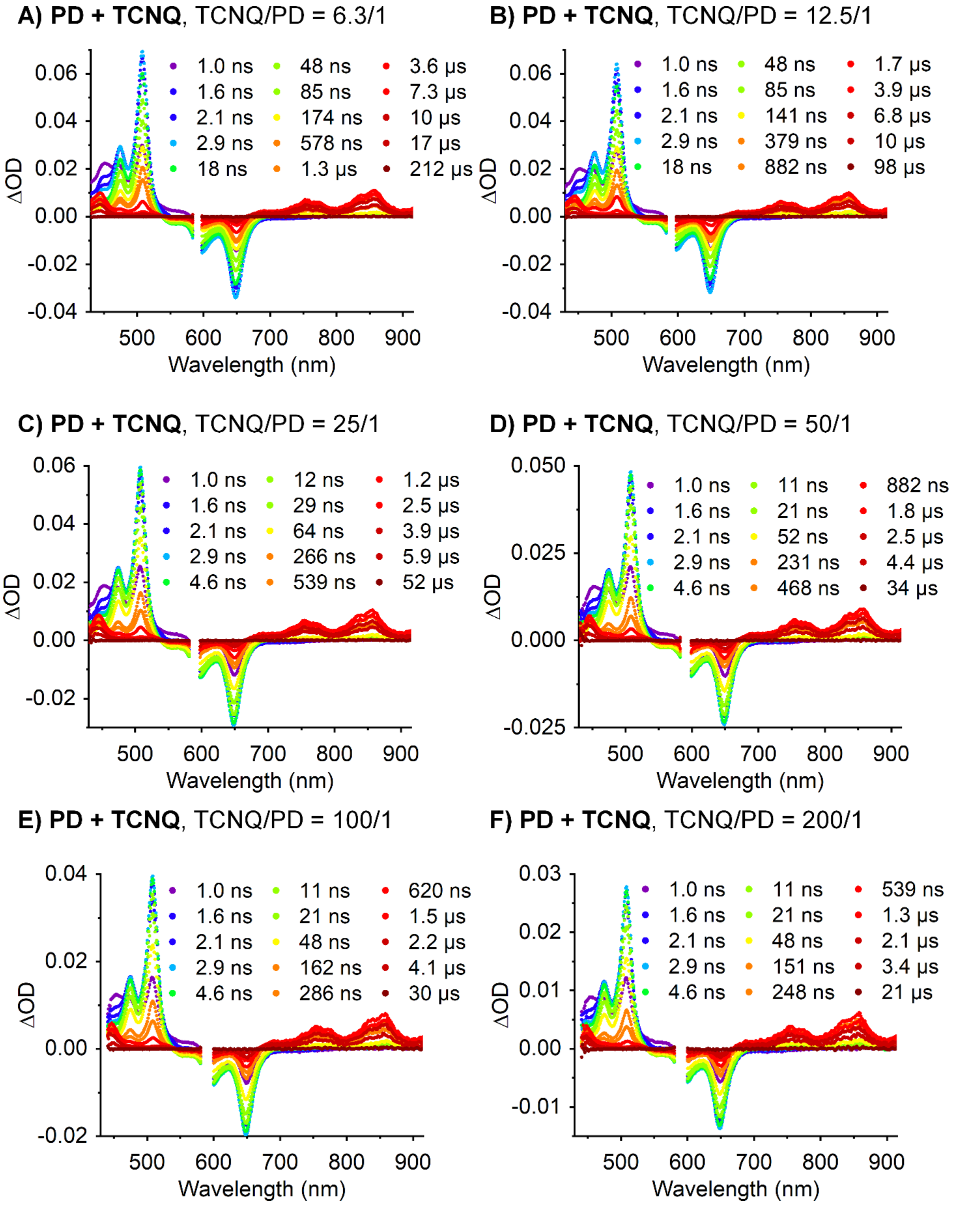

Figure S22. nsTA data of different **TCNQ/PD** mixtures (see captions) in argon-saturated BN measured at room temperature upon nanosecond pump-probe experiments (590 nm) with several time delays (see legends).
A) **PD** [2.42x10^{-5} M], **TCNQ** [1.52x10^{-4} M]. B) **PD** [2.35x10^{-5} M], **TCNQ** [2.94x10^{-4} M]. C) **PD** [2.22x10^{-5} M], **TCNQ** [5.56x10^{-4} M]. D) **PD** [2.00x10^{-5} M], **TCNQ** [1.00x10^{-3} M]. E) **PD** [1.67x10^{-5} M], **TCNQ** [1.67x10^{-3} M]. F) **PD** [1.25x10^{-5} M], **TCNQ** [2.50x10^{-3} M].

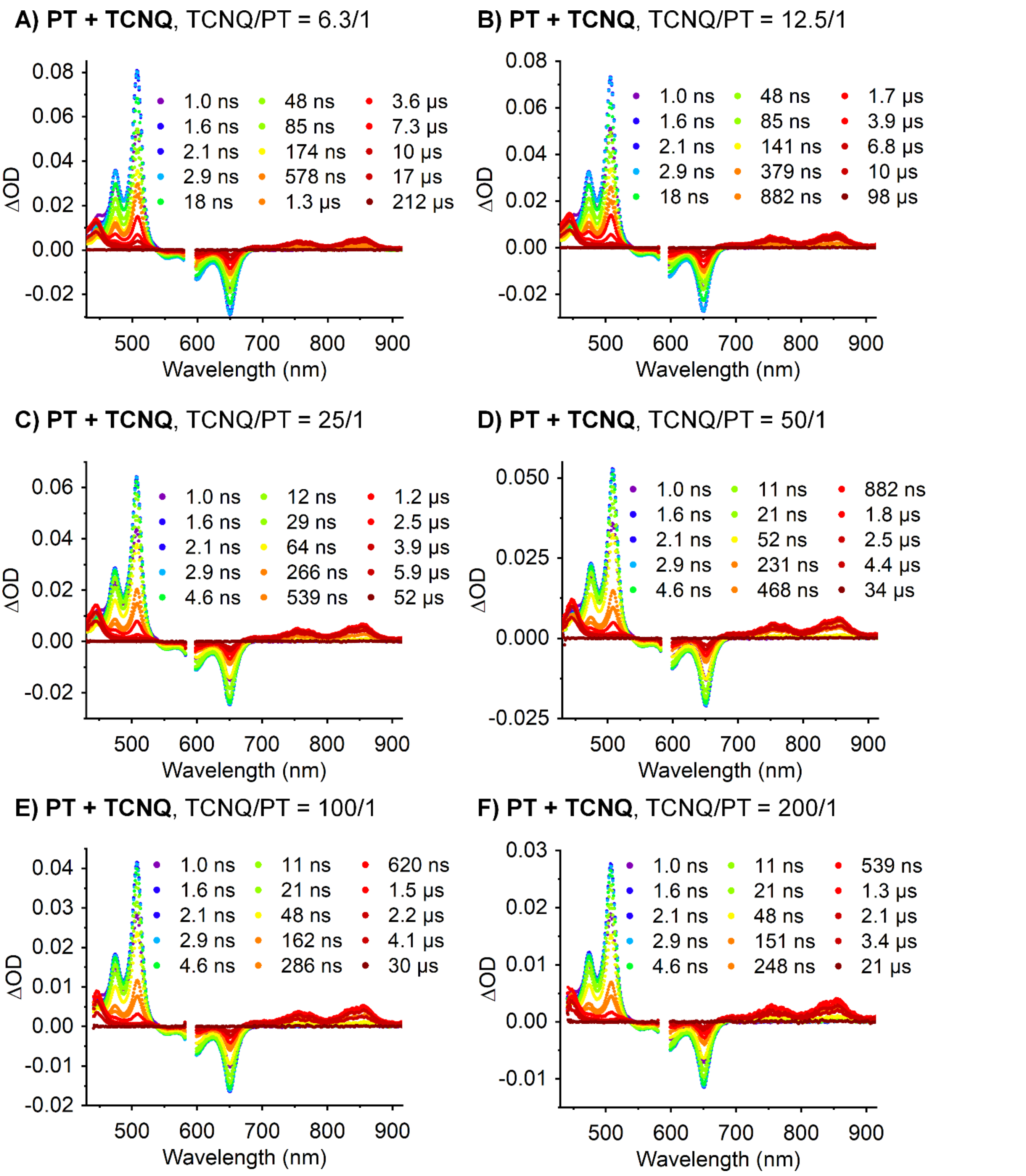

Figure S23. nsTA data of different **TCNQ/PT** mixtures (see captions) in argon-saturated BN measured at room temperature upon nanosecond pump-probe experiments (590 nm) with several time delays (see legends). A) **PT** [2.42×10^{-5} M], **TCNQ** [1.52×10^{-4} M]. B) **PT** [2.35×10^{-5} M], **TCNQ** [2.94×10^{-4} M]. C) **PT** [2.22×10^{-5} M], **TCNQ** [5.56×10^{-4} M]. D) **PT** [2.00×10^{-5} M], **TCNQ** [1.00×10^{-3} M]. E) **PT** [1.67×10^{-5} M], **TCNQ** [1.67×10^{-3} M]. F) **PT** [1.25×10^{-5} M], **TCNQ** [2.50×10^{-3} M].

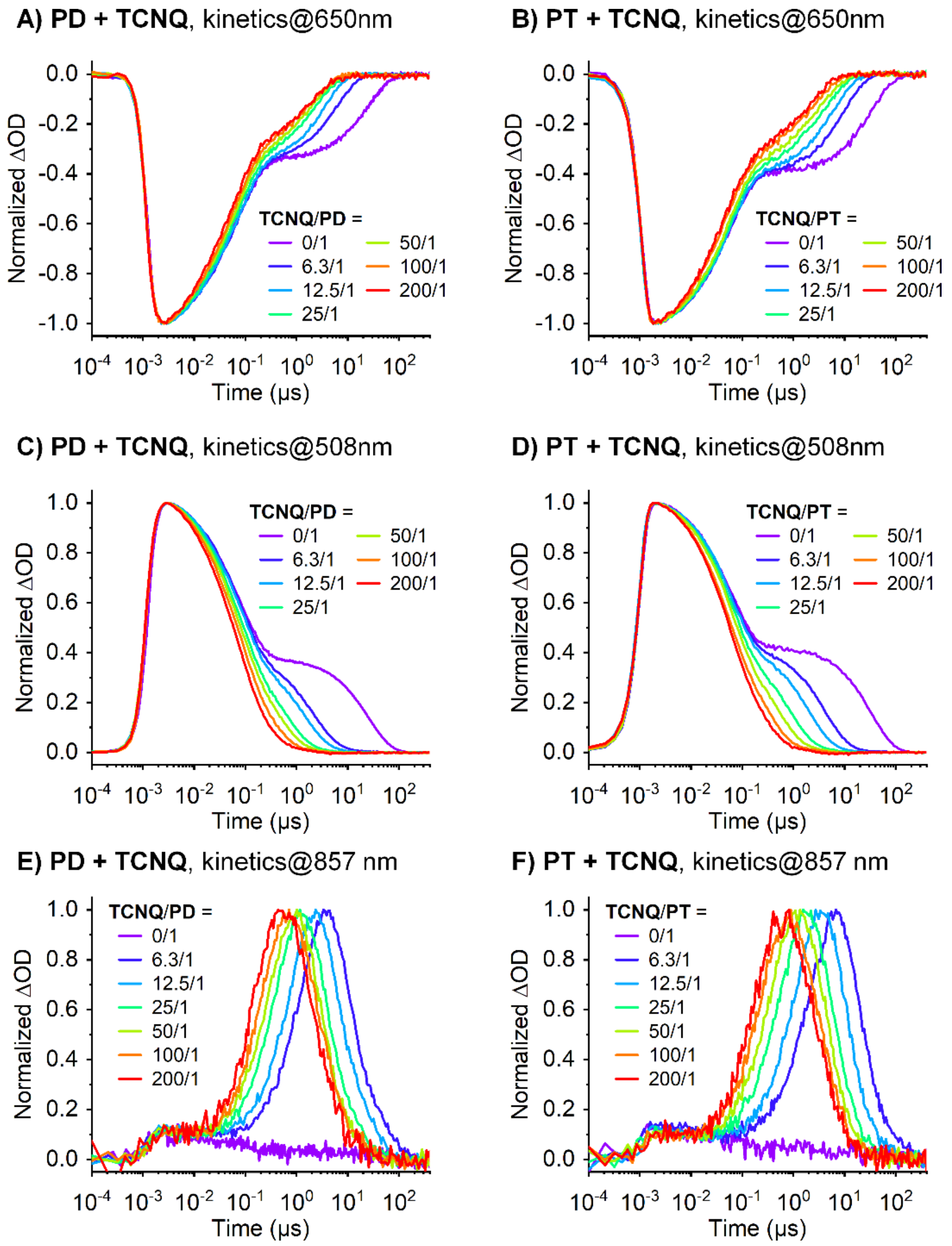

Figure S24. Raw data time absorption profiles for different **TCNQ**/pentacene ratios, corroborating the dependence of the pentacene triplet excited states` lifetimes on the **TCNQ** excess.
See **Figures S21-S23** for the nsTA spectra (BN solvent).

Table S6. Lifetimes of the transient states of **PD** under the addition of different amounts of **TCNQ** in benzonitrile.[a]

$\frac{c(TCNQ)}{c(PD)}$	**τ[(S$_1$S$_0$)]**	**τ[^{1}(T$_1$T$_1$)]**	**τ[^{5}(T$_1$T$_1$)]**	**τ[(T$_1$+T$_1$)]^b**	**τ[(P$^{•+}$+TCNQ$^{•-}$)]**
0 / 1	0.40 ns	8.3 ns	91 ns	32 µs	---
6.3 / 1	0.40 ns	9.7 ns	78 ns	1.8 µs	12 µs
12.5 / 1	0.40 ns	11 ns	76 ns	1.2 µs	7.3 µs
25 / 1	0.40 ns	9.7 ns	67 ns	543 ns	4.6 µs
50 / 1	0.40 ns	8.6 ns	61 ns	385 ns	3.4 µs
100 / 1	0.40 ns	9.8 ns	55 ns	255 ns	3.5 µs
200 / 1	0.40 ns	8.0 ns	48 ns	180 ns	2.9 µs

[a] All Lifetimes were extracted from nsTA measurements.

[b] The EAS of the (T_1+T_1) state shows minor contributions of a parallel formed charge separated state ($P^{•+}$ + $TCNQ^{•-}$).

See **Figures S21 and S26-S31** for the global analyses.

Table S7. Lifetimes of the transient states of PT under the addition of different amounts of TCNQ in benzonitrile.[a]

$\frac{c(TCNQ)}{c(PT)}$	**τ[(S$_1$S$_0$S$_0$S$_0$)]**	**τ[^{1}(T$_1$T$_1$S$_0$S$_0$)]**	**τ[^{5}(T$_1$T$_1$S$_0$S$_0$)]**	**τ[(T$_1$+T$_1$+S$_0$+S$_0$)]^b**	**τ[(P$^{•+}$+TCNQ$^{•-}$)]**
0 / 1	0.14 ns	12 ns	70 ns	32 µs	---
6.3 / 1	0.14 ns	10 ns	64 ns	3.8 µs	15 µs
12.5 / 1	0.12 ns	7.4 ns	62 ns	1.8 µs	8.7 µs
25 / 1	0.12 ns	7.7 ns	59 ns	830 ns	6.0 µs
50 / 1	0.11 ns	7.2 ns	56 ns	507 ns	4.6 µs
100 / 1	0.11 ns	9.3 ns	47 ns	316 ns	3.6 µs
200 / 1	0.13 ns	8.3 ns	45 ns	240 ns	3.0 µs

[a] All Lifetimes were extracted from nsTA measurements (τ[($S_1S_0S_0S_0$)] is too short for a meaningful extraction from nsTA data).

[b] The EAS of the (T_1+T_1+S_0+S_0) state shows minor contributions of a parallel formed charge separated state ($P^{•+}$ + $TCNQ^{•-}$).

See **Figures S21 and S26-S31** for global analyses.

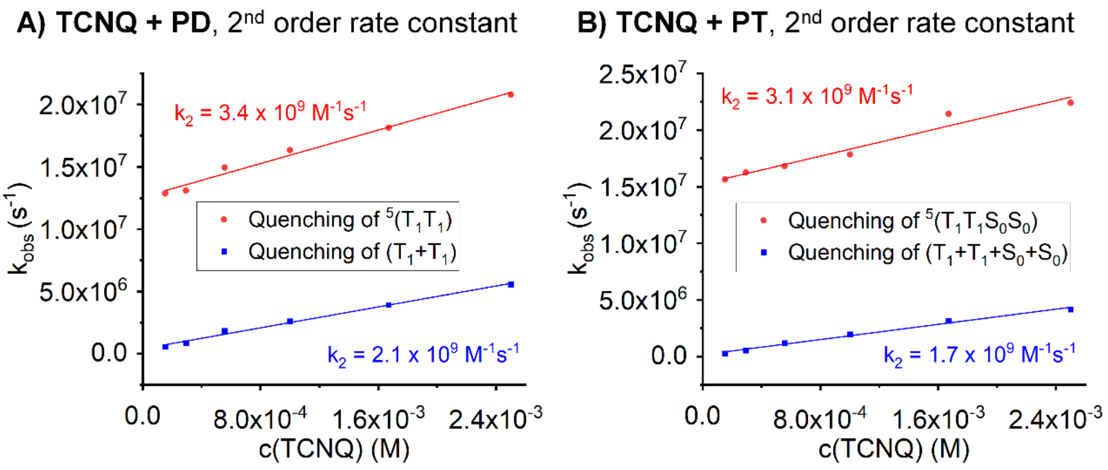

Figure S25. Plot of the pseudo-first-order rate constant versus the **TCNQ** concentration in argon-saturated benzonitrile at room temperature to calculate the second order rate constant for charge transfer.
See chapter "Determination of the second order charge-transfer rate constant from global analysis" for details.
See **Figure S26-S31** for the global analysis.

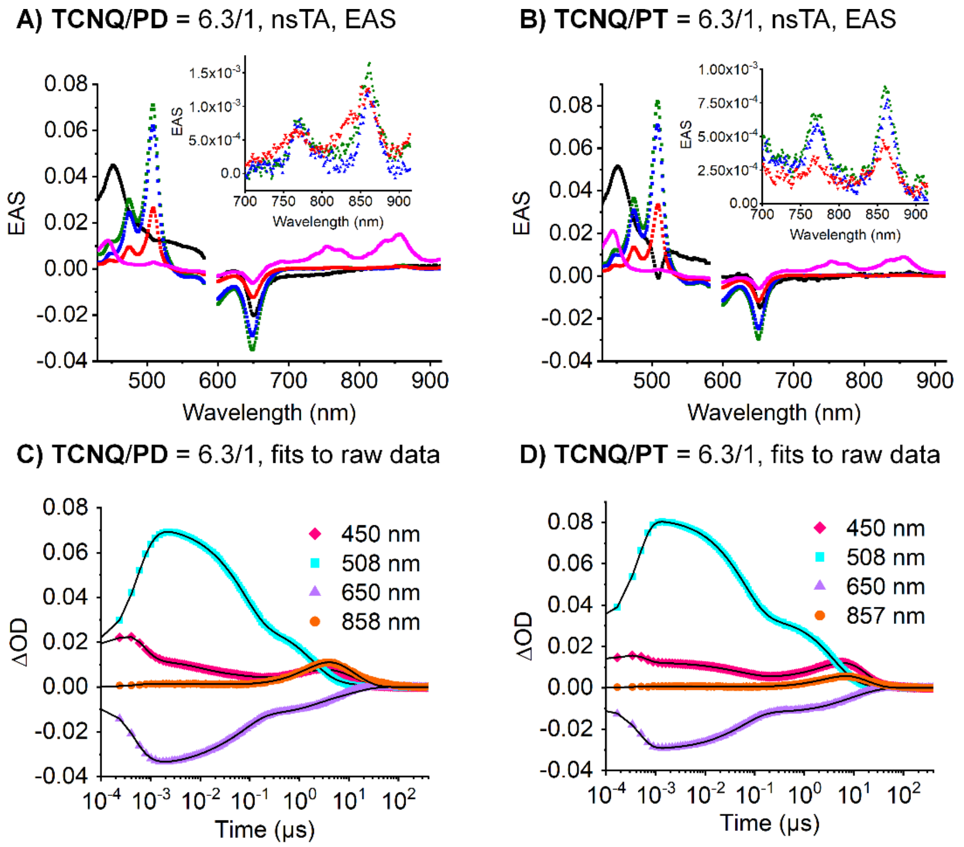

Legend for EAS of TCNQ/PD in A):

- (S_1S_0)
- $^1(T_1T_1)$
- $^5(T_1T_1)$
- (T_1+T_1) + $(P^{\bullet+}$ + $TCNQ^{\bullet-})$
- $(P^{\bullet+}$ + $TCNQ^{\bullet-})$

Legend for EAS of TCNQ/PT in B):

- $(S_1S_0S_0S_0)$
- $^1(T_1T_1S_0S_0)$
- $^5(T_1T_1S_0S_0)$
- $(T_1+T_1+S_0+S_0)$
- $(P^{\bullet+}$ + $TCNQ^{\bullet-})$

Figure S26. nsTA data of **PD** [2.42x10^{-5} M] and **PT** [2.42x10^{-5} M] upon addition of **TCNQ** [1.52x10^{-4} M] in argon-saturated benzonitrile (BN) measured at room.
A&B) Deconvoluted EAS of the singlet excited state (S_1S_0) / $(S_1S_0S_0S_0)$ state after solvent relaxation (black), the correlated triplet pair state with singlet spin $^1(T_1T_1)$ / $^1(T_1T_1S_0S_0)$ (green), the correlated triplet pair state with quintet spin $^5(T_1T_1)$ / $^5(T_1T_1S_0S_0)$ (blue), the decorrelated triplet state (T_1+T_1) / $(T_1+T_1+S_0+S_0)$ with traces of the charge separated state $(P^{\bullet+}$ + $TCNQ^{\bullet-})$ (red) and the charge separated state $(P^{\bullet+}$ + $TCNQ^{\bullet-})$ (pink) of **PD** / **PT** (C / D) upon addition of TCNQ as obtained by global analysis with a sequential model of the TA data shown above. Insets: Zoom-in on the triplet / $TCNQ^{\bullet-}$ features between 700 and 915 nm. C&D) Raw data single-wavelength kinetics and fits to the data. See **Figures S22-S23** for the raw data transient absorption spectra.

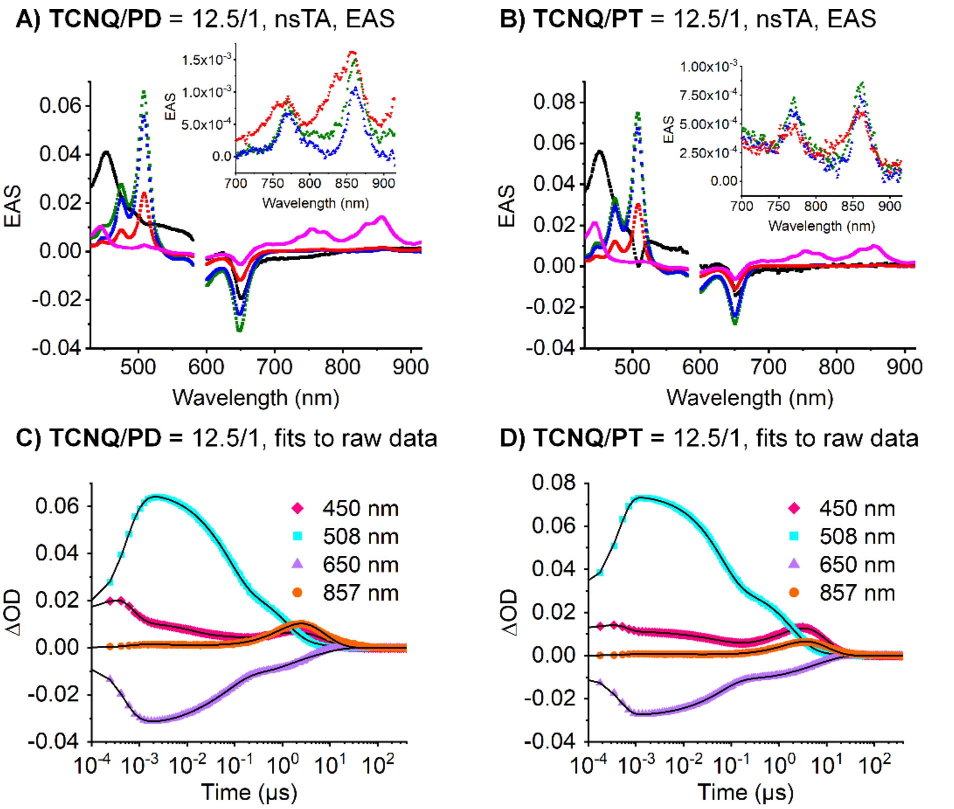

Legend for EAS of TCNQ/PD in A):

- (S_1S_0)
- $^1(T_1T_1)$
- $^5(T_1T_1)$
- (T_1+T_1) + $(P^{\bullet+}$ + $TCNQ^{\bullet-})$
- $(P^{\bullet+}$ + $TCNQ^{\bullet-})$

Legend for EAS of TCNQ/PT in B):

- $(S_1S_0S_0S_0)$
- $^1(T_1T_1S_0S_0)$
- $^5(T_1T_1S_0S_0)$
- $(T_1+T_1+S_0+S_0)$ + $(P^{\bullet+}$ + $TCNQ^{\bullet-})$
- $(P^{\bullet+}$ + $TCNQ^{\bullet-})$

Figure S27. nsTA data of **PD** [2.35x10^{-5} M] and **PT** [2.35x10^{-5} M] upon addition of **TCNQ** [2.94x10^{-4} M] in argon-saturated benzonitrile (BN) measured at room.
A&B) Deconvoluted EAS of the singlet excited state (S_1S_0) / $(S_1S_0S_0S_0)$ state after solvent relaxation (black), the correlated triplet pair state with singlet spin $^1(T_1T_1)$ / $^1(T_1T_1S_0S_0)$ (green), the correlated triplet pair state with quintet spin $^5(T_1T_1)$ / $^5(T_1T_1S_0S_0)$ (blue), the decorrelated triplet state (T_1+T_1) / $(T_1+T_1+S_0+S_0)$ with traces of the charge separated state $(P^{\bullet+}$ + $TCNQ^{\bullet-})$ (red) and the charge separated state $(P^{\bullet+}$ + $TCNQ^{\bullet-})$ (pink) of **PD** / **PT** (C / D) upon addition of TCNQ as obtained by global analysis with a sequential model of the TA data shown above. Insets: Zoom-in on the triplet / $TCNQ^{\bullet-}$ features between 700 and 915 nm. C&D) Raw data single-wavelength kinetics and fits to the data. See **Figures S22-S23** for the raw data transient absorption spectra.

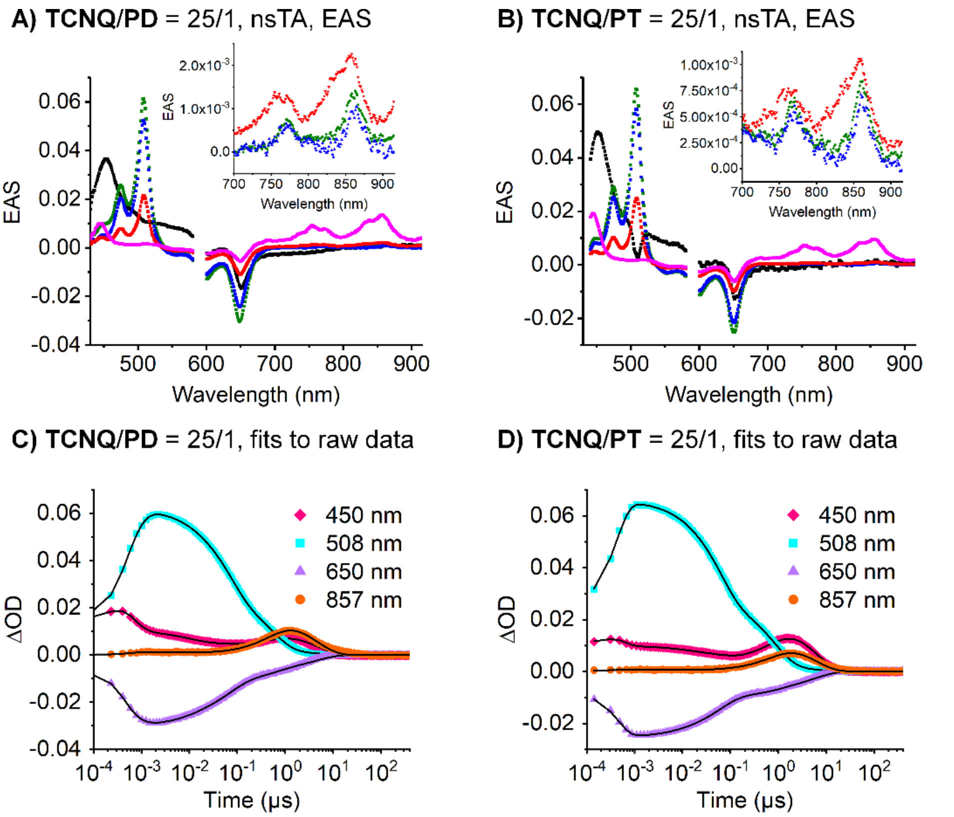

Legend for EAS of TCNQ/PD in A):

- (S_1S_0)
- $^1(T_1T_1)$
- $^5(T_1T_1)$
- (T_1+T_1) + $(P^{\bullet+}$ + $TCNQ^{\bullet-})$
- $(P^{\bullet+}$ + $TCNQ^{\bullet-})$

Legend for EAS of TCNQ/PT in B):

- $(S_1S_0S_0S_0)$
- $^1(T_1T_1S_0S_0)$
- $^5(T_1T_1S_0S_0)$
- $(T_1+T_1+S_0+S_0)$ + $(P^{\bullet+}$ + $TCNQ^{\bullet-})$
- $(P^{\bullet+}$ + $TCNQ^{\bullet-})$

Figure S28. nsTA data of **PD** [2.22x10^{-5} M] and **PT** [2.22x10^{-5} M] upon addition of **TCNQ** [5.56x10^{-4} M] in argon-saturated benzonitrile (BN) measured at room.
A&B) Deconvoluted EAS of the singlet excited state (S_1S_0) / $(S_1S_0S_0S_0)$ state after solvent relaxation (black), the correlated triplet pair state with singlet spin $^1(T_1T_1)$ / $^1(T_1T_1S_0S_0)$ (green), the correlated triplet pair state with quintet spin $^5(T_1T_1)$ / $^5(T_1T_1S_0S_0)$ (blue), the decorrelated triplet state (T_1+T_1) / $(T_1+T_1+S_0+S_0)$ with traces of the charge separated state $(P^{\bullet+}$ + $TCNQ^{\bullet-})$ (red) and the charge separated state $(P^{\bullet+}$ + $TCNQ^{\bullet-})$ (pink) of **PD** / **PT** (C / D) upon addition of TCNQ as obtained by global analysis with a sequential model of the TA data shown above. Insets: Zoom-in on the triplet / $TCNQ^{\bullet-}$ features between 700 and 915 nm. C&D) Raw data single-wavelength kinetics and fits to the data. See **Figures S22-S23** for the raw data transient absorption spectra.

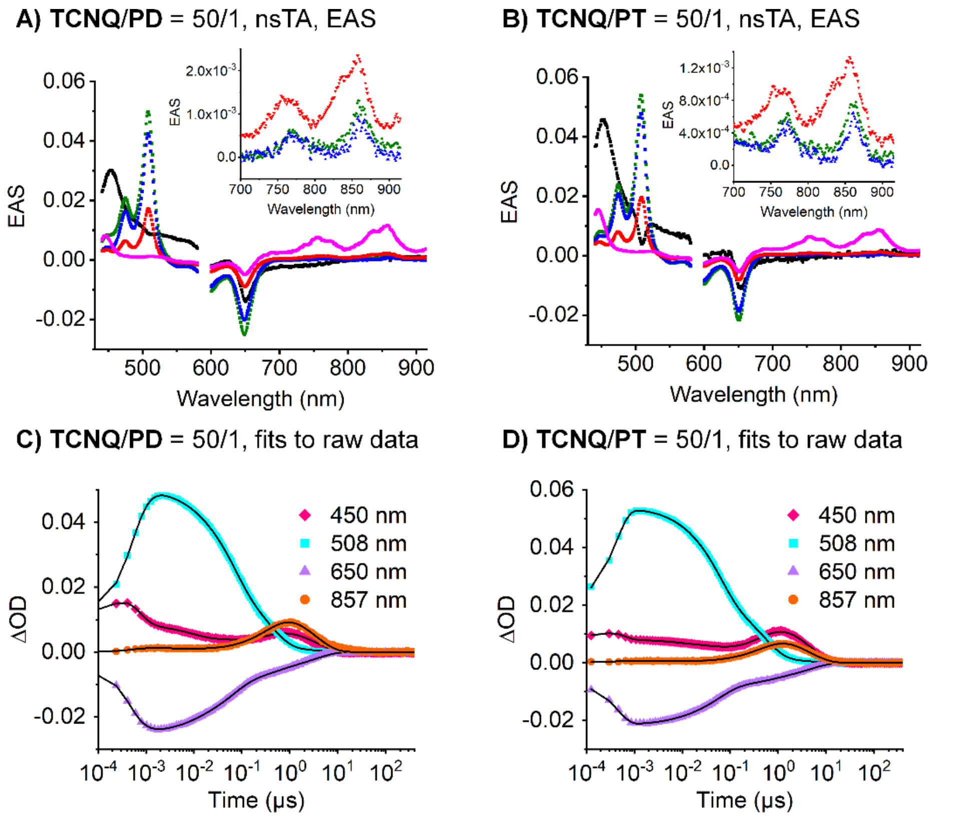

Legend for EAS of TCNQ/PD in A):

- (S_1S_0)
- $^1(T_1T_1)$
- $^5(T_1T_1)$
- (T_1+T_1) + $(P^{\bullet+}$ + $TCNQ^{\bullet-})$
- $(P^{\bullet+}$ + $TCNQ^{\bullet-})$

Legend for EAS of TCNQ/PT in B):

- $(S_1S_0S_0S_0)$
- $^1(T_1T_1S_0S_0)$
- $^5(T_1T_1S_0S_0)$
- $(T_1+T_1+S_0+S_0)$ + $(P^{\bullet+}$ + $TCNQ^{\bullet-})$
- $(P^{\bullet+}$ + $TCNQ^{\bullet-})$

Figure S29. nsTA data of **PD** [2.00x10^{-5} M] and **PT** [2.00x10^{-5} M] upon addition of **TCNQ** [1.00x10^{-3} M] in argon-saturated benzonitrile (BN) measured at room.
A&B) Deconvoluted EAS of the singlet excited state (S_1S_0) / $(S_1S_0S_0S_0)$ state after solvent relaxation (black), the correlated triplet pair state with singlet spin $^1(T_1T_1)$ / $^1(T_1T_1S_0S_0)$ (green), the correlated triplet pair state with quintet spin $^5(T_1T_1)$ / $^5(T_1T_1S_0S_0)$ (blue), the decorrelated triplet state (T_1+T_1) / $(T_1+T_1+S_0+S_0)$ with traces of the charge separated state $(P^{\bullet+}$ + $TCNQ^{\bullet-})$ (red) and the charge separated state $(P^{\bullet+}$ + $TCNQ^{\bullet-})$ (pink) of **PD** / **PT** (C / D) upon addition of TCNQ as obtained by global analysis with a sequential model of the TA data shown above. Insets: Zoom-in on the triplet / $TCNQ^{\bullet-}$ features between 700 and 915 nm. C&D) Raw data single-wavelength kinetics and fits to the data. See **Figures S22-S23** for the raw data transient absorption spectra.

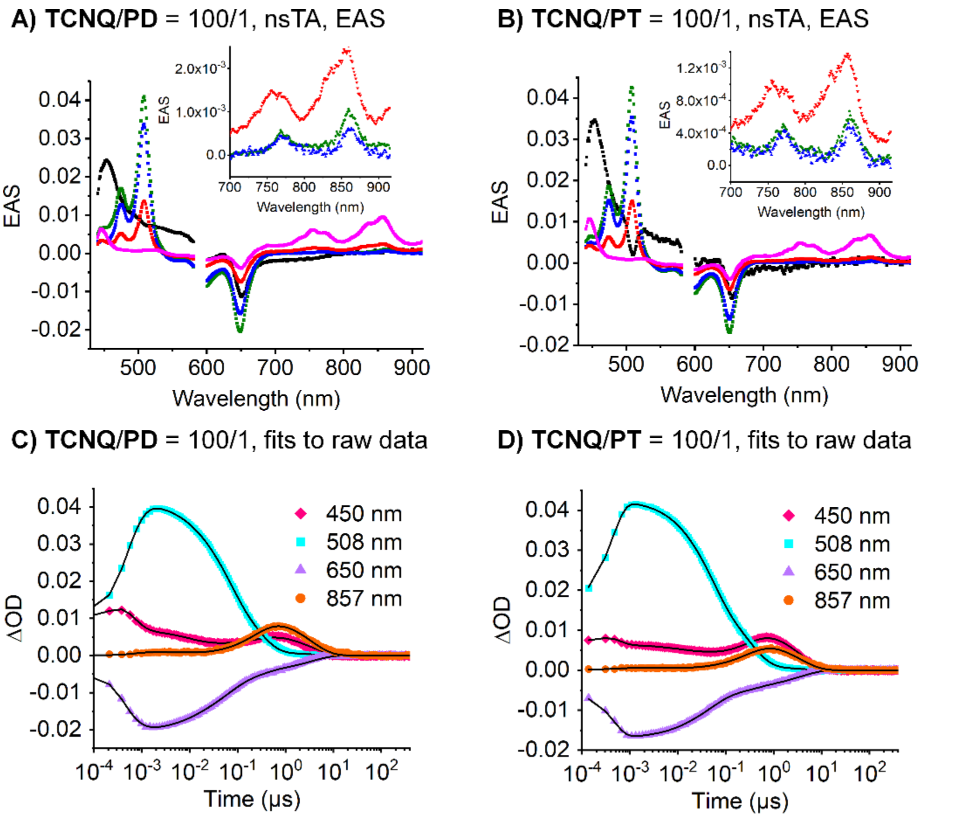

Legend for EAS of TCNQ/PD in A):

- (S_1S_0)
- $^1(T_1T_1)$
- $^5(T_1T_1)$
- (T_1+T_1) + $(P^{\bullet+}$ + $TCNQ^{\bullet-})$
- $(P^{\bullet+}$ + $TCNQ^{\bullet-})$

Legend for EAS of TCNQ/PT in B):

- $(S_1S_0S_0S_0)$
- $^1(T_1T_1S_0S_0)$
- $^5(T_1T_1S_0S_0)$
- $(T_1+T_1+S_0+S_0)$ + $(P^{\bullet+}$ + $TCNQ^{\bullet-})$
- $(P^{\bullet+}$ + $TCNQ^{\bullet-})$

Figure S30. nsTA data of **PD** [1.67x10^{-5} M] and **PT** [1.67x10^{-5} M] upon addition of **TCNQ** [1.67x10^{-3} M] in argon-saturated benzonitrile (BN) measured at room.
A&B) Deconvoluted EAS of the singlet excited state (S_1S_0) / $(S_1S_0S_0S_0)$ state after solvent relaxation (black), the correlated triplet pair state with singlet spin $^1(T_1T_1)$ / $^1(T_1T_1S_0S_0)$ (green), the correlated triplet pair state with quintet spin $^5(T_1T_1)$ / $^5(T_1T_1S_0S_0)$ (blue), the decorrelated triplet state (T_1+T_1) / $(T_1+T_1+S_0+S_0)$ with traces of the charge separated state $(P^{\bullet+}$ + $TCNQ^{\bullet-})$ (red) and the charge separated state $(P^{\bullet+}$ + $TCNQ^{\bullet-})$ (pink) of **PD** / **PT** (C / D) upon addition of TCNQ as obtained by global analysis with a sequential model of the TA data shown above. Insets: Zoom-in on the triplet / $TCNQ^{\bullet-}$ features between 700 and 915 nm. C&D) Raw data single-wavelength kinetics and fits to the data. See **Figures S22-S23** for the raw data transient absorption spectra.

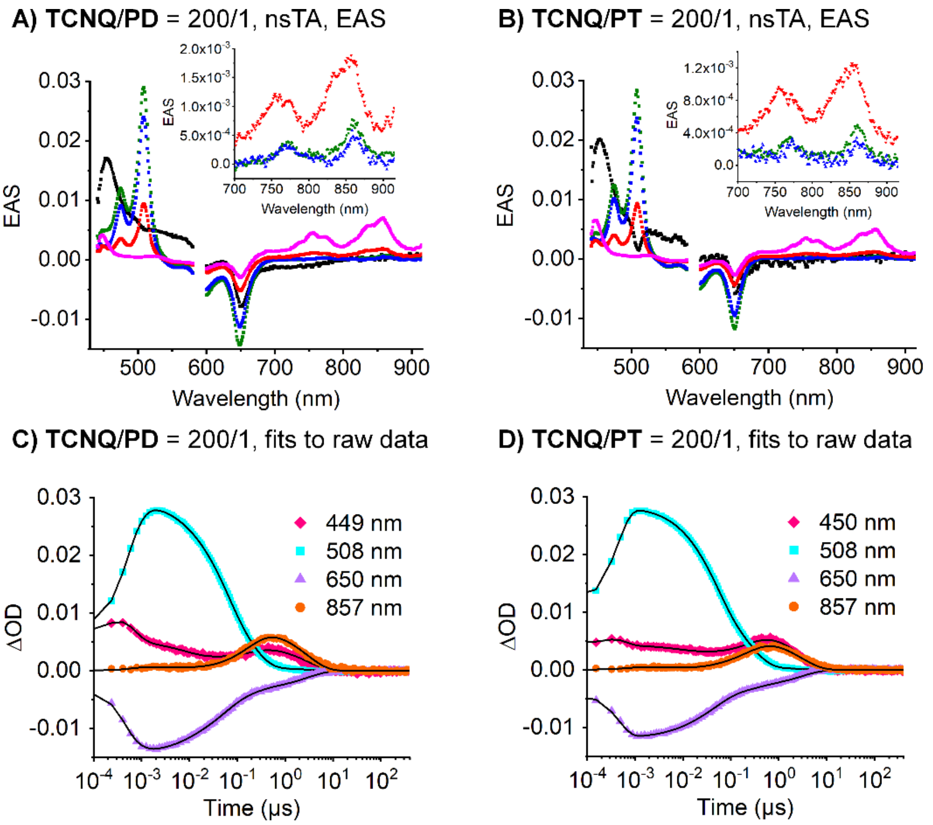

Legend for EAS of TCNQ/PD in A):

- (S_1S_0)
- $^1(T_1T_1)$
- $^5(T_1T_1)$
- (T_1+T_1) + $(P^{\bullet+}$ + $TCNQ^{\bullet-})$
- $(P^{\bullet+}$ + $TCNQ^{\bullet-})$

Legend for EAS of TCNQ/PT in B):

- $(S_1S_0S_0S_0)$
- $^1(T_1T_1S_0S_0)$
- $^5(T_1T_1S_0S_0)$
- $(T_1+T_1+S_0+S_0)$ + $(P^{\bullet+}$ + $TCNQ^{\bullet-})$
- $(P^{\bullet+}$ + $TCNQ^{\bullet-})$

Figure S31. nsTA data of **PD** [1.25x10^{-5} M] and **PT** [1.25x10^{-5} M] upon addition of **TCNQ** [2.50x10^{-3} M] in argon-saturated benzonitrile (BN) measured at room.
A&B) Deconvoluted EAS of the singlet excited state (S_1S_0) / $(S_1S_0S_0S_0)$ state after solvent relaxation (black), the correlated triplet pair state with singlet spin $^1(T_1T_1)$ / $^1(T_1T_1S_0S_0)$ (green), the correlated triplet pair state with quintet spin $^5(T_1T_1)$ / $^5(T_1T_1S_0S_0)$ (blue), the decorrelated triplet state (T_1+T_1) / $(T_1+T_1+S_0+S_0)$ with traces of the charge separated state $(P^{\bullet+}$ + $TCNQ^{\bullet-})$ (red) and the charge separated state $(P^{\bullet+}$ + $TCNQ^{\bullet-})$ (pink) of **PD** / **PT** (C / D) upon addition of TCNQ as obtained by global analysis with a sequential model of the TA data shown above. Insets: Zoom-in on the triplet / $TCNQ^{\bullet-}$ features between 700 and 915 nm. C&D) Raw data single-wavelength kinetics and fits to the data. See **Figures S22-S23** for the raw data transient absorption spectra.

*Target analysis of the charge-transfer reaction of **PD** and **PT** with **TCNQ** under the assumption that **PD** / **PT** can be oxidized twice by reacting with two **TCNQ** molecules*

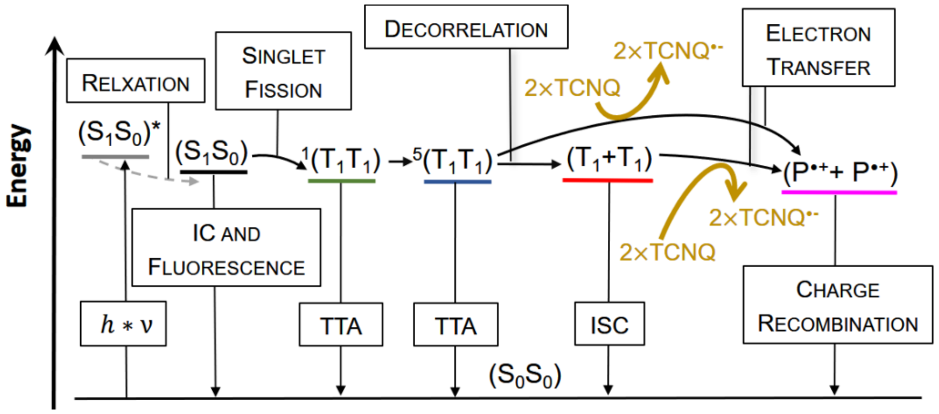

Figure S32. Energy diagram and kinetic model used to fit the TA data of **PD** and **PT** upon addition of **TCNQ** in benzonitrile via target analysis under the assumption that a dimer / tetramer can be oxidized twice by reacting with two TCNQ molecules.

Please note that the first relaxation step is only resolvable in fsTA experiments and is thus not fit in the target analysis of the nsTA data. For simplification, delayed fluorescence is not implemented in this kinetic model. The energy diagram of **PT** can be generated, if the excited states are renamed accordingly: $(S_1S_0)^*$ changes to $(S_1S_0S_0S_0)^*$, (S_1S_0) changes to $(S_1S_0S_0S_0)$, $^1(T_1T_1)$ changes to $^1(T_1T_1S_0S_0)$, $^5(T_1T_1)$ changes to $^5(T_1T_1S_0S_0)$, $(T_1 + T_1)$ changes to $(T_1 + T_1 + S_0 + S_0)$, and $(P^{\bullet+} + P^{\bullet+})$ changes to $(P^{\bullet+} + P^{\bullet+} + S_0 + S_0)$. Abbreviation list: *h*v (initial photoexcitation), IC (internal conversion), TTA (triplet-triplet-annihilation), ISC (intersystem crossing).*

A) PD [1.25x10^{-5}M] + **TCNQ** [2.50x10^{-3}M] (**TCNQ/PD** = 200/1), nsTA, target analysis

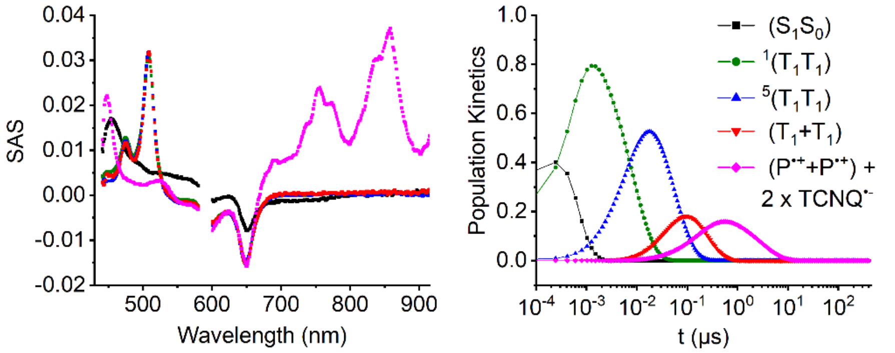

B) PT [1.25x10^{-5}M] + **TCNQ** [2.50x10^{-3}M] (**TCNQ/PT** = 200/1), nsTA, target analysis

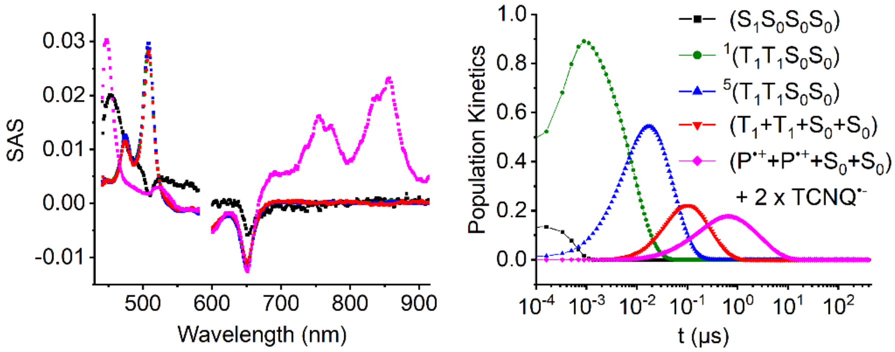

Figure S33. Species associated spectra (SAS) and the corresponding population kinetics of the nsTA data of **PD** [1.25x10^{-5} M] and **PT** [1.25x10^{-5} M] upon addition of **TCNQ** [2.50x10^{-3} M] in argon-saturated benzonitrile as obtained by target analysis under the assumption that **PD / PT** can be oxidized twice by reacting with two TCNQ molecules.
See **Figure S32** for the kinetic model and **Table S8** for the used rate constants. Multiplying the SAS on the left by the corresponding population kinetics on the right yields the complete 3-D TA data set (ΔOD versus time and wavelength).

Table S8. Rate constants and quantum yields for **PD** [$1.25x10^{-5}$ M] and **PT** [$1.25x10^{-5}$ M] upon addition of **TCNQ** [$2.50x10^{-3}$ M] using the kinetic model shown in **Figure S32** under the assumption that a dimer / tetramer can be oxidized twice by reacting with two TCNQ molecules.[a]

Initial State	Resulting State	Rate Constant / Quantum Yield [b]	
		**PD**	**PT**
(S_1S_0) / $(S_1S_0S_0S_0)$	$^1(T_1T_1)$ / $^1(T_1T_1S_0S_0)$	2.39×10^9 s^{-1} / 94%	8.00×10^9 s^{-1} / 100%
	(S_0S_0) / $(S_0S_0S_0S_0)$	1.53×10^8 s^{-1} / 6%	0.00 s^{-1} / 0%
$^1(T_1T_1)$ / $^1(T_1T_1S_0S_0)$	$^5(T_1T_1)$ / $^5(T_1T_1S_0S_0)$	1.00×10^8 s^{-1} / 80%	9.68×10^7 s^{-1} / 80%
	(S_0S_0) / $(S_0S_0S_0S_0)$	2.50×10^7 s^{-1} / 20%	2.42×10^7 s^{-1} / 20%
$^5(T_1T_1)$ / $^5(T_1T_1S_0S_0)$	(T_1+T_1) / $(T_1+T_1+S_0+S_0)$	8.11×10^6 s^{-1} / 39%	9.18×10^6 s^{-1} / 41%
	$(P^{\bullet+}+P^{\bullet+})$ / $(P^{\bullet+}+P^{\bullet+}+S_0+S_0)$	1.25×10^6 s^{-1} / 6%	1.57×10^6 s^{-1} / 7%
	(S_0S_0) / $(S_0S_0S_0S_0)$	1.14×10^7 s^{-1} / 55%	1.16×10^7 s^{-1} / 52%
(T_1+T_1) / $(T_1+T_1+S_0+S_0)$	$(P^{\bullet+}+P^{\bullet+})$ / $(P^{\bullet+}+P^{\bullet+}+S_0+S_0)$	2.78×10^6 s^{-1} / 50%	2.08×10^6 s^{-1} / 50%
	(S_0S_0) / $(S_0S_0S_0S_0)$	2.78×10^6 s^{-1} / 50%	2.08×10^6 s^{-1} / 50%
$(P^{\bullet+}+P^{\bullet+})$ / $(P^{\bullet+}+P^{\bullet+}+S_0+S_0)$	(S_0S_0) / $(S_0S_0S_0S_0)$	3.49×10^5 s^{-1} / 100%	3.30×10^5 s^{-1} / 100%

[a] See **Figure S33** for the corresponding target analyses.

[b] The quantum yield is always given with respect to the initial state on the left side of the corresponding row. It is calculated from the target analysis as the ratio of the corresponding rate and the sum over all rates that lead to a deactivation of the corresponding initial state.

*Target analysis of the charge-transfer reaction of **PD** and **PT** with **TCNQ** under the assumption that **PD** / **PT** can be oxidized only once by reacting with **TCNQ**.*

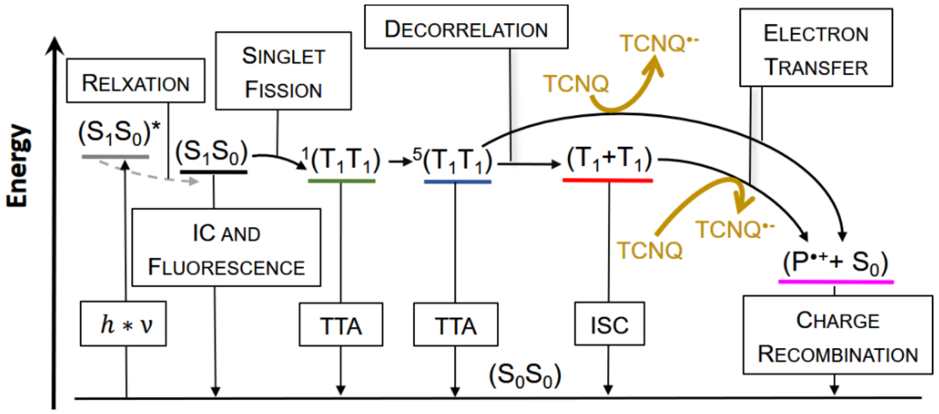

Figure S34. Energy diagram and kinetic model used to fit the TA data of **PD** and **PT** upon addition of **TCNQ** in benzonitrile via target analysis under the assumption that a dimer / tetramer can be oxidized only once by reacting with **TCNQ**.
Please note that the first relaxation step is only resolvable in fsTA experiments and is thus not fit in the target analysis of the nsTA data. For simplification, delayed fluorescence is not implemented in this kinetic model. The energy diagram of **PT** can be generated, if the excited states are renamed accordingly: $(S_1S_0)^*$ changes to $(S_1S_0S_0S_0)^*$, (S_1S_0) changes to $(S_1S_0S_0S_0)$, $^1(T_1T_1)$ changes to $^1(T_1T_1S_0S_0)$, $^5(T_1T_1)$ changes to $^5(T_1T_1S_0S_0)$, $(T_1 + T_1)$ changes to $(T_1 + T_1 + S_0 + S_0)$, and $(P^{•+} + S_0)$ changes to $(P^{•+} + S_0 + S_0 + S_0)$. Abbreviation list: *h*v (initial photoexcitation), IC (internal conversion), TTA (triplet-triplet-annihilation), ISC (intersystem crossing).*

A) PD [2.42x10^{-5}M] + **TCNQ** [1.52x10^{-4}M] (**TCNQ/PD** = 6.3/1), nsTA, target analysis

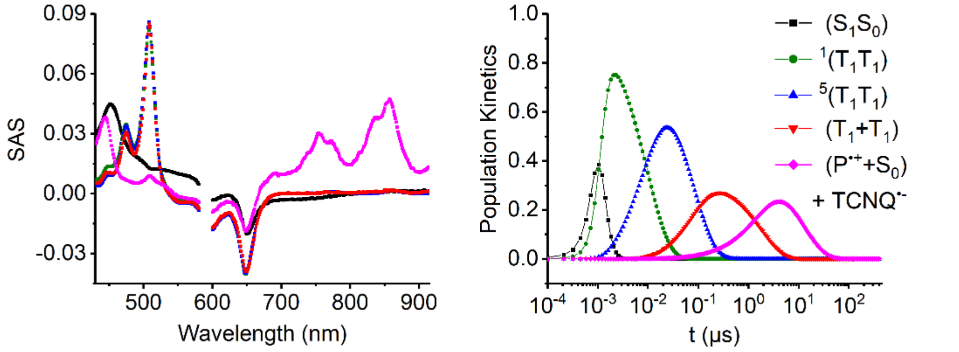

B) PT [2.42x10^{-5}M] + **TCNQ** [1.52x10^{-4}M] (**TCNQ/PT** = 6.3/1), nsTA, target analysis

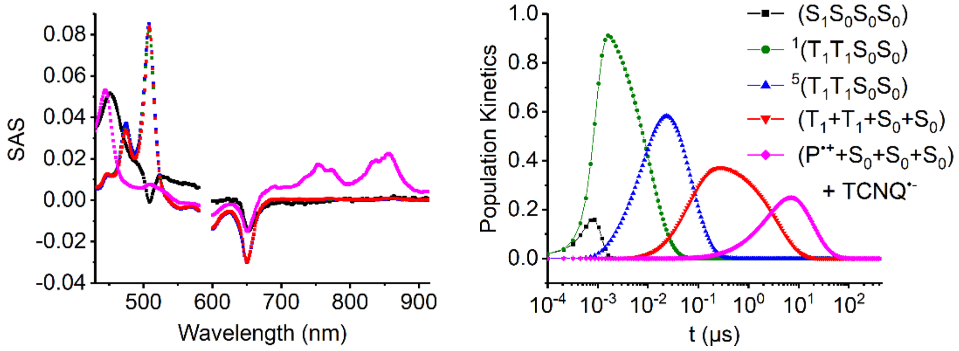

Figure S35. Species associated spectra (SAS) and the corresponding population kinetics of the nsTA data of **PD** [2.42x10^{-5} M] and **PT** [2.42x10^{-5} M] upon addition of **TCNQ** [1.52x10^{-4} M] in argon-saturated benzonitrile as obtained by target analysis under the assumption that **PD / PT** can be oxidized only once by reacting with **TCNQ**. See **Figure S34** for the kinetic model and **Table S9** for the used rate constants. Multiplying the SAS on the left by the corresponding population kinetics on the right yields the complete 3-D TA data set (ΔOD versus time and wavelength).

Table S9. Rate constants and quantum yields for **PD** [2.42x10^{-5} M] and **PT** [2.42x10^{-5} M] upon addition of **TCNQ** [1.52x10^{-4} M] using the kinetic model shown in **Figure S34** under the assumption that a dimer / tetramer can be oxidized only once by reacting with **TCNQ** molecules.[a]

Initial State	Resulting State	Rate Constant / Quantum Yield[b]	
		PD	PT
(S_1S_0) / ($S_1S_0S_0S_0$)	^{1}(T_1T_1) / ^{1}($T_1T_1S_0S_0$)	2.23 x 10^9 s^{-1} / 87%	7.10 x 10^9 s^{-1} / 100%
	(S_0S_0) / ($S_0S_0S_0S_0$)	3.34 x 10^8 s^{-1} / 13%	0 s^{-1} / 0%
^{1}(T_1T_1) / ^{1}($T_1T_1S_0S_0$)	^{5}(T_1T_1) / ^{5}($T_1T_1S_0S_0$)	8.28 x 10^7 s^{-1} / 83%	7.95 x 10^7 s^{-1} / 83%
	(S_0S_0) / ($S_0S_0S_0S_0$)	1.76 x 10^7 s^{-1} / 17%	1.63 x 10^7 s^{-1} / 17%
^{5}(T_1T_1) / ^{5}($T_1T_1S_0S_0$)	(T_1+T_1) / (T_1+T_1+S_0+S_0)	5.53 x 10^6 s^{-1} / 43%	7.51 x 10^6 s^{-1} / 48%
	($P^{\bullet+}$+S_0) / ($P^{\bullet+}$+S_0+S_0+S_0)	2.57 x 10^5 s^{-1} / 2%	0 s^{-1} / 0%
	(S_0S_0) / ($S_0S_0S_0S_0$)	7.08 x 10^6 s^{-1} / 55%	8.14 x 10^6 s^{-1} / 52%
(T_1+T_1) / (T_1+T_1+S_0+S_0)	($P^{\bullet+}$+S_0) / ($P^{\bullet+}$+S_0+S_0+S_0)	5.53 x 10^5 s^{-1} / 100%	2.65 x 10^5 s^{-1} / 100%
($P^{\bullet+}$+S_0) / ($P^{\bullet+}$+S_0+S_0+S_0)	(S_0S_0) / ($S_0S_0S_0S_0$)	8.17 x 10^4 s^{-1} / 100%	6.84 x 10^4 s^{-1} / 100%

[a] See **Figure 35** for the corresponding target analyses.

[b] The quantum yield is always given with respect to the initial state on the left side of the corresponding row. It is calculated from the target analysis as the ratio of the corresponding rate and the sum over all rates that lead to a deactivation of the corresponding initial state.

A) PD [2.35x10^{-5}M] + **TCNQ** [2.94x10^{-4}M] (**TCNQ/PD** = 12.5/1), nsTA, target analysis

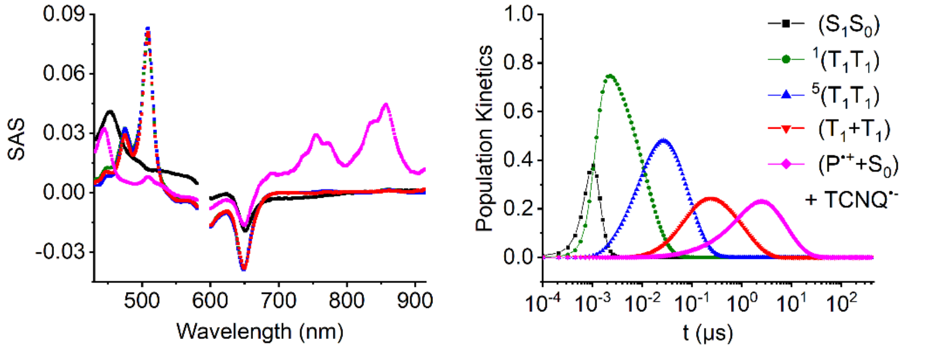

B) PT [2.35x10^{-5}M] + **TCNQ** [2.94x10^{-4}M] (**TCNQ/PT** = 12.5/1), nsTA, target analysis

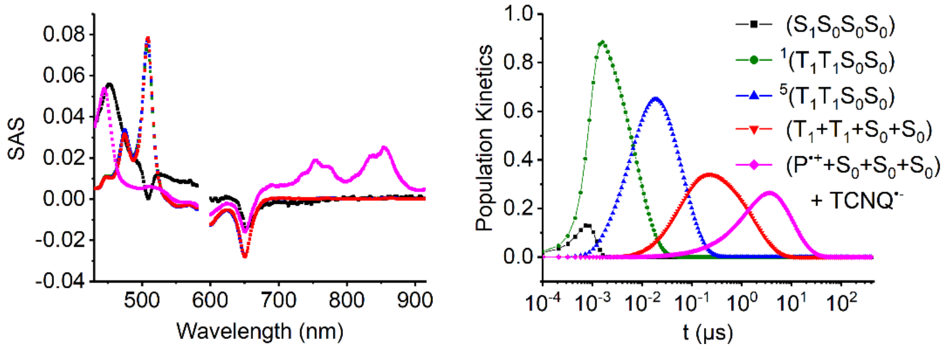

Figure S36. Species associated spectra (SAS) and the corresponding population kinetics of the nsTA data of **PD** [2.35x10^{-5} M] and **PT** [2.35x10^{-5} M] upon addition of **TCNQ** [2.94x10^{-4} M] in argon-saturated benzonitrile as obtained by target analysis under the assumption that **PD / PT** can be oxidized only once by reacting with **TCNQ**. See **Figure S34** for the kinetic model and **Table S10** for the used rate constants. Multiplying the SAS on the left by the corresponding population kinetics on the right yields the complete 3-D TA data set (ΔOD versus time and wavelength).

Table S10. Rate constants and quantum yields for **PD** [2.35x10^{-5} M] and **PT** [2.35x10^{-5} M] upon addition of **TCNQ** [2.94x10^{-4} M] using the kinetic model shown in **Figure S34** under the assumption that a dimer / tetramer can be oxidized only once by reacting with **TCNQ** molecules.[a]

Initial State	Resulting State	Rate Constant / Quantum Yield[b]	
		**PD**	**PT**
(S_1S_0) / $(S_1S_0S_0S_0)$	$^1(T_1T_1)$ / $^1(T_1T_1S_0S_0)$	2.20 x 10^9 s^{-1} / 85%	8.50 x 10^9 s^{-1} / 100%
	(S_0S_0) / $(S_0S_0S_0S_0)$	3.88 x 10^8 s^{-1} / 15%	0 s^{-1} / 0%
$^1(T_1T_1)$ / $^1(T_1T_1S_0S_0)$	$^5(T_1T_1)$ / $^5(T_1T_1S_0S_0)$	6.90 x 10^7 s^{-1} / 79%	1.17 x 10^8 s^{-1} / 87%
	(S_0S_0) / $(S_0S_0S_0S_0)$	1.83 x 10^7 s^{-1} / 21%	1.75 x 10^7 s^{-1} / 13%
$^5(T_1T_1)$ / $^5(T_1T_1S_0S_0)$	(T_1+T_1) / $(T_1+T_1+S_0+S_0)$	5.76 x 10^6 s^{-1} / 44%	7.15 x 10^6 s^{-1} / 44%
	$(P^{•+}+S_0)$ / $(P^{•+}+S_0+S_0+S_0)$	5.89 x 10^5 s^{-1} / 4.5%	3.25 x 10^5 s^{-1} / 2%
	(S_0S_0) / $(S_0S_0S_0S_0)$	6.74 x 10^6 s^{-1} / 51.5%	8.78 x 10^6 s^{-1} / 54%
(T_1+T_1) / $(T_1+T_1+S_0+S_0)$	$(P^{•+}+S_0)$ / $(P^{•+}+S_0+S_0+S_0)$	8.68 x 10^5 s^{-1} / 100%	5.43 x 10^5 s^{-1} / 100%
$(P^{•+}+S_0)$ / $(P^{•+}+S_0+S_0+S_0)$	(S_0S_0) / $(S_0S_0S_0S_0)$	1.38 x 10^5 s^{-1} / 100%	1.15 x 10^5 s^{-1} / 100%

[a] See **Figure 36** for the corresponding target analyses.

[b] The quantum yield is always given with respect to the initial state on the left side of the corresponding row. It is calculated from the target analysis as the ratio of the corresponding rate and the sum over all rates that lead to a deactivation of the corresponding initial state.

A) PD [2.22x10^{-5}M] + **TCNQ** [5.56x10^{-4}M] (**TCNQ/PD** = 25/1), nsTA, target analysis

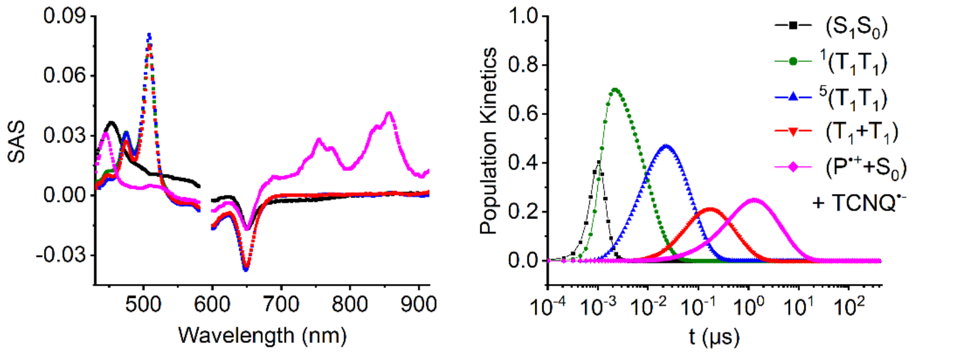

B) PT [2.22x10^{-5}M] + **TCNQ** [5.56x10^{-4}M] (**TCNQ/PT** = 25/1), nsTA, target analysis

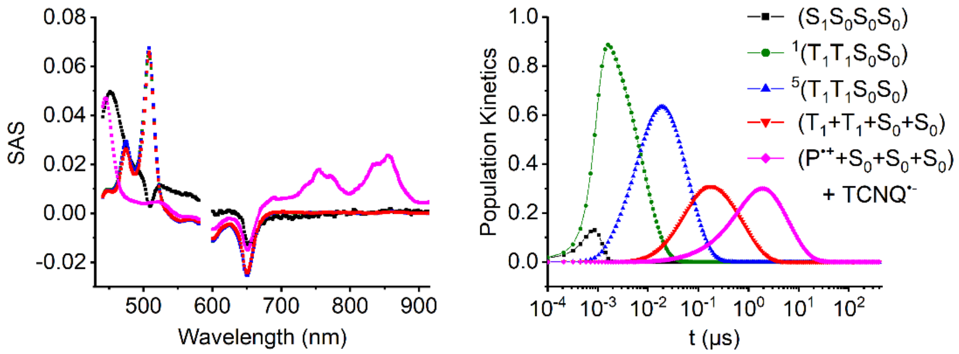

Figure S37. Species associated spectra (SAS) and the corresponding population kinetics of the nsTA data of **PD** [2.22x10^{-5} M] and **PT** [2.22x10^{-5} M] upon addition of **TCNQ** [5.56x10^{-4} M] in argon-saturated benzonitrile as obtained by target analysis under the assumption that **PD / PT** can be oxidized only once by reacting with **TCNQ**. See **Figure S34** for the kinetic model and **Table S11** for the used rate constants. Multiplying the SAS on the left by the corresponding population kinetics on the right yields the complete 3-D TA data set (ΔOD versus time and wavelength).

Table S11. Rate constants and quantum yields for **PD** [2.22x10^{-5} M] and **PT** [2.22x10^{-5} M] upon addition of **TCNQ** [5.56x10^{-4} M] using the kinetic model shown in **Figure S34** under the assumption that a dimer / tetramer can be oxidized only once by reacting with **TCNQ** molecules.[a]

Initial State	Resulting State	Rate Constant / Quantum Yield[b]	
		PD	PT
(S_1S_0) / $(S_1S_0S_0S_0)$	$^1(T_1T_1)$ / $^1(T_1T_1S_0S_0)$	2.04 x 10^9 s^{-1} / 81%	8.50 x 10^9 s^{-1} / 100%
	(S_0S_0) / $(S_0S_0S_0S_0)$	4.79 x 10^8 s^{-1} / 19%	0 s^{-1} / 0%
$^1(T_1T_1)$ / $^1(T_1T_1S_0S_0)$	$^5(T_1T_1)$ / $^5(T_1T_1S_0S_0)$	8.26 x 10^7 s^{-1} / 80%	1.12 x 10^8 s^{-1} / 86%
	(S_0S_0) / $(S_0S_0S_0S_0)$	2.07 x 10^7 s^{-1} / 20%	1.82 x 10^7 s^{-1} / 14%
$^5(T_1T_1)$ / $^5(T_1T_1S_0S_0)$	(T_1+T_1) / $(T_1+T_1+S_0+S_0)$	6.58 x 10^6 s^{-1} / 44%	7.40 x 10^6 s^{-1} / 44%
	$(P^{•+}+S_0)$ / $(P^{•+}+S_0+S_0+S_0)$	1.05 x 10^6 s^{-1} / 7%	6.73 x 10^5 s^{-1} / 4%
	(S_0S_0) / $(S_0S_0S_0S_0)$	7.33 x 10^6 s^{-1} / 49%	8.75 x 10^6 s^{-1} / 52%
(T_1+T_1) / $(T_1+T_1+S_0+S_0)$	$(P^{•+}+S_0)$ / $(P^{•+}+S_0+S_0+S_0)$	1.84 x 10^6 s^{-1}/ 100%	1.21 x 10^6 s^{-1}/ 100%
$(P^{•+}+S_0)$ / $(P^{•+}+S_0+S_0+S_0)$	(S_0S_0) / $(S_0S_0S_0S_0)$	2.19 x 10^5 s^{-1}/ 100%	1.68 x 10^5 s^{-1}/ 100%

[a] See **Figure 37** for the corresponding target analyses.

[b] The quantum yield is always given with respect to the initial state on the left side of the corresponding row. It is calculated from the target analysis as the ratio of the corresponding rate and the sum over all rates that lead to a deactivation of the corresponding initial state.

A) PD [2.00x10^{-5}M] + **TCNQ** [1.00x10^{-3}M] (**TCNQ/PD** = 50/1), nsTA, target analysis

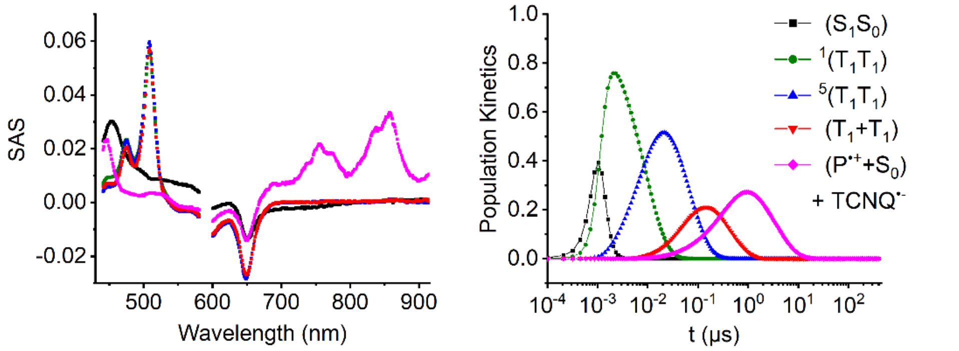

B) PT [2.00x10^{-5}M] + **TCNQ** [1.00x10^{-3}M] (**TCNQ/PT** = 50/1), nsTA, target analysis

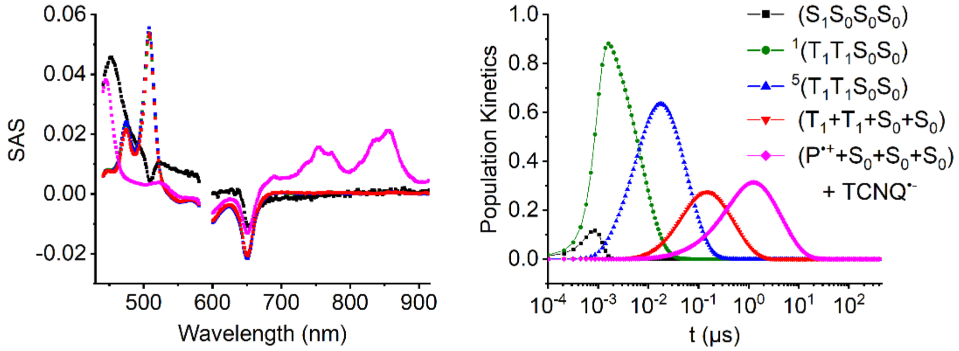

Figure S38. Species associated spectra (SAS) and the corresponding population kinetics of the nsTA data of **PD** [2.00x10^{-5} M] and **PT** [2.00x10^{-5} M] upon addition of **TCNQ** [1.00x10^{-3} M] in argon-saturated benzonitrile as obtained by target analysis under the assumption that **PD / PT** can be oxidized only once by reacting with TCNQ. See **Figure S34** for the kinetic model and **Table S12** for the used rate constants. Multiplying the SAS on the left by the corresponding population kinetics on the right yields the complete 3-D TA data set (ΔOD versus time and wavelength).

Table S12. Rate constants and quantum yields for **PD** [2.00x10^{-5} M] and **PT** [2.00x10^{-5} M] upon addition of **TCNQ** [1.00x10^{-3} M] using the kinetic model shown in **Figure S34** under the assumption that a dimer / tetramer can be oxidized only once by reacting with **TCNQ** molecules.[a]

**Initial State**	**Resulting State**	**Rate Constant / Quantum Yield**[b]	
		**PD**	**PT**
(S_1S_0) / ($S_1S_0S_0S_0$)	^{1}(T_1T_1) / ^{1}($T_1T_1S_0S_0$)	2.28 x 10^9 s^{-1} / 89%	9.50 x 10^9 s^{-1} / 100%
	(S_0S_0) / ($S_0S_0S_0S_0$)	2.82 x 10^8 s^{-1} / 11%	0 s^{-1} / 0%
^{1}(T_1T_1) / ^{1}($T_1T_1S_0S_0$)	^{5}(T_1T_1) / ^{5}($T_1T_1S_0S_0$)	9.26 x 10^7 s^{-1} / 80%	1.20 x 10^8 s^{-1} / 86%
	(S_0S_0) / ($S_0S_0S_0S_0$)	2.32 x 10^7 s^{-1} / 20%	1.96 x 10^7 s^{-1} / 14%
^{5}(T_1T_1) / ^{5}($T_1T_1S_0S_0$)	(T_1+T_1) / (T_1+T_1+S_0+S_0)	6.87 x 10^6 s^{-1} / 42%	7.50 x 10^6 s^{-1} / 42%
	($P^{•+}$+S_0) / ($P^{•+}$+S_0+S_0+S_0)	1.39 x 10^6 s^{-1} / 8.5%	1.07 x 10^6 s^{-1} / 6%
	(S_0S_0) / ($S_0S_0S_0S_0$)	8.09 x 10^6 s^{-1} / 49.5%	9.28 x 10^6 s^{-1} / 52%
(T_1+T_1) / (T_1+T_1+S_0+S_0)	($P^{•+}$+S_0) / ($P^{•+}$+S_0+S_0+S_0)	2.60 x 10^6 s^{-1} / 100%	1.97 x 10^6 s^{-1} / 100%
($P^{•+}$+S_0) / ($P^{•+}$+S_0+S_0+S_0)	(S_0S_0) / ($S_0S_0S_0S_0$)	2.93 x 10^5 s^{-1} / 100%	2.19 x 10^5 s^{-1} / 100%

[a] See **Figure 38** for the corresponding target analyses.

[b] The quantum yield is always given with respect to the initial state on the left side of the corresponding row. It is calculated from the target analysis as the ratio of the corresponding rate and the sum over all rates that lead to a deactivation of the corresponding initial state.

A) PD [1.67x10^{-5}M] + **TCNQ** [1.67x10^{-3}M] (**TCNQ/PD** = 100/1), nsTA, target analysis

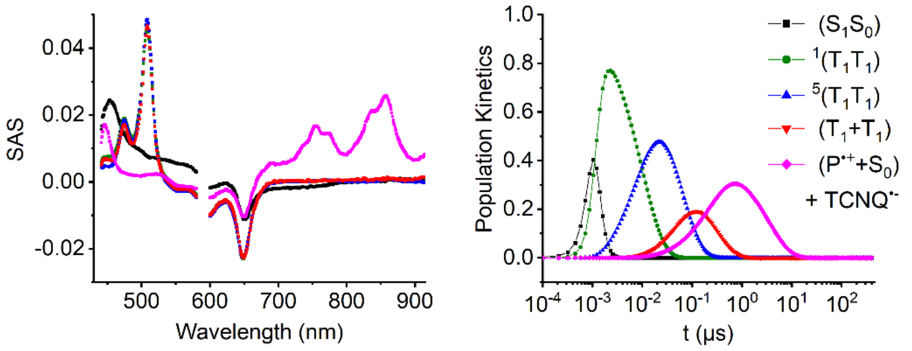

B) PT [1.67x10^{-5}M] + **TCNQ** [1.67x10^{-3}M] (**TCNQ/PT** = 100/1), nsTA, target analysis

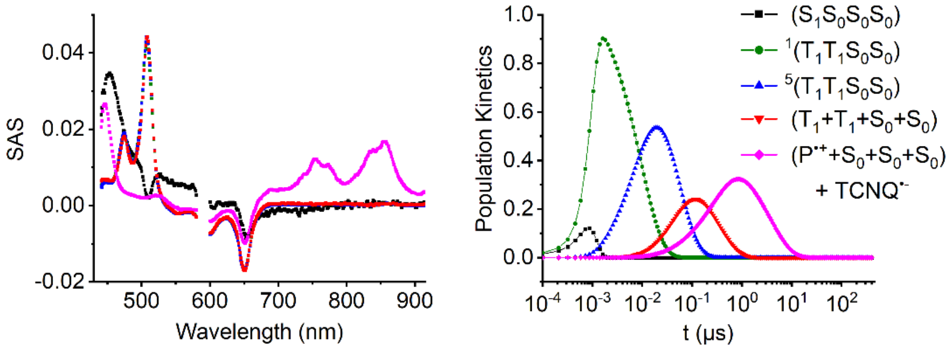

Figure S39. Species associated spectra (SAS) and the corresponding population kinetics of the nsTA data of **PD** [1.67x10^{-5} M] and **PT** [1.67x10^{-5} M] upon addition of **TCNQ** [1.67x10^{-3} M] in argon-saturated benzonitrile as obtained by target analysis under the assumption that **PD / PT** can be oxidized only once by reacting with **TCNQ**. See **Figure S34** for the kinetic model and **Table S13** for the used rate constants. Multiplying the SAS on the left by the corresponding population kinetics on the right yields the complete 3-D TA data set (ΔOD versus time and wavelength).

Table S13. Rate constants and quantum yields for **PD** [1.67x10^{-5} M] and **PT** [1.67x10^{-5} M] upon addition of **TCNQ** [1.67x10^{-3} M] using the kinetic model shown in **Figure S34** under the assumption that a dimer / tetramer can be oxidized only once by reacting with **TCNQ** molecules.[a]

Initial State	Resulting State	Rate Constant / Quantum Yield[b]	
		PD	PT
(S_1S_0) / $(S_1S_0S_0S_0)$	$^1(T_1T_1)$ / $^1(T_1T_1S_0S_0)$	2.26 x 10^9 s^{-1} / 89%	9.00 x 10^9 s^{-1} / 100%
	(S_0S_0) / $(S_0S_0S_0S_0)$	2.79 x 10^8 s^{-1} / 11%	0 s^{-1} / 0%
$^1(T_1T_1)$ / $^1(T_1T_1S_0S_0)$	$^5(T_1T_1)$ / $^5(T_1T_1S_0S_0)$	7.94 x 10^7 s^{-1} / 78%	8.64 x 10^7 s^{-1} / 80%
	(S_0S_0) / $(S_0S_0S_0S_0)$	2.24 x 10^7 s^{-1} / 22%	2.16 x 10^7 s^{-1} / 20%
$^5(T_1T_1)$ / $^5(T_1T_1S_0S_0)$	(T_1+T_1) / $(T_1+T_1+S_0+S_0)$	7.62 x 10^6 s^{-1} / 42%	8.82 x 10^6 s^{-1} / 42%
	$(P^{\bullet+}+S_0)$ / $(P^{\bullet+}+S_0+S_0+S_0)$	2.18 x 10^6 s^{-1} / 12%	1.89 x 10^6 s^{-1} / 9%
	(S_0S_0) / $(S_0S_0S_0S_0)$	8.34 x 10^6 s^{-1} / 46%	1.03 x 10^7 s^{-1} / 49%
(T_1+T_1) / $(T_1+T_1+S_0+S_0)$	$(P^{\bullet+}+S_0)$ / $(P^{\bullet+}+S_0+S_0+S_0)$	3.92 x 10^6 s^{-1} / 100%	3.16 x 10^6 s^{-1}/ 100%
$(P^{\bullet+}+S_0)$ / $(P^{\bullet+}+S_0+S_0+S_0)$	(S_0S_0) / $(S_0S_0S_0S_0)$	2.88 x 10^5 s^{-1} / 100%	2.75 x 10^5 s^{-1} / 100%

[a] See **Figure 39** for the corresponding target analyses.

[b] The quantum yield is always given with respect to the initial state on the left side of the corresponding row. It is calculated from the target analysis as the ratio of the corresponding rate and the sum over all rates that lead to a deactivation of the corresponding initial state.

PD [1.25x10^{-5}M] + **TCNQ** [2.50x10^{-3}M] (**TCNQ/PD** = 200/1), nsTA, target analysis

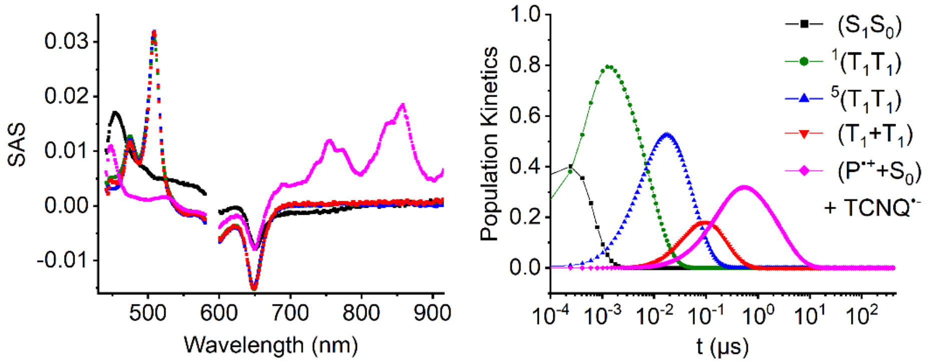

Figure S40. Species associated spectra (SAS) and the corresponding population kinetics of the nsTA data of **PD** [1.25x10^{-5} M] upon addition of **TCNQ** [2.50x10^{-3} M] in argon-saturated benzonitrile as obtained by target analysis under the assumption that **PD** can be oxidized only once by reacting with **TCNQ**.
See **Figure S34** for the kinetic model and **Table S14** for the used rate constants. Multiplying the SAS on the left by the corresponding population kinetics on the right yields the complete 3-D TA data set (ΔOD versus time and wavelength).

Table S14. Rate constants and quantum yields for **PD** [1.25x10^{-5} M] and **PT** [1.25x10^{-5} M] upon addition of **TCNQ** [2.50x10^{-3} M] using the kinetic model shown in **Figure S34** under the assumption that a dimer / tetramer can be oxidized only once by reacting with **TCNQ** molecules.[a]

Initial State	Resulting State	Rate Constant / Quantum Yield[b]	
		PD	PT
(S_1S_0) / ($S_1S_0S_0S_0$)	$^1(T_1T_1)$ / $^1(T_1T_1S_0S_0)$	2.39 x 10^9 s^{-1} / 94%	8.00 x 10^9 s^{-1} / 100%
	(S_0S_0) / ($S_0S_0S_0S_0$)	1.53 x 10^8 s^{-1} / 6%	0.00 s^{-1} / 0%
$^1(T_1T_1)$ / $^1(T_1T_1S_0S_0)$	$^5(T_1T_1)$ / $^5(T_1T_1S_0S_0)$	1.00 x 10^8 s^{-1} / 80%	9.68 x 10^7 s^{-1} / 80%
	(S_0S_0) / ($S_0S_0S_0S_0$)	2.50 x 10^7 s^{-1} / 20%	2.42 x 10^7 s^{-1} / 20%
$^5(T_1T_1)$ / $^5(T_1T_1S_0S_0)$	(T_1+T_1) / (T_1+T_1+S_0+S_0)	8.11 x 10^6 s^{-1} / 39%	9.18 x 10^6 s^{-1} / 41%
	($P^{\bullet+}$+S_0) / ($P^{\bullet+}$+S_0+S_0+S_0)	2.50 x 10^6 s^{-1} / 12%	3.14 x 10^6 s^{-1} / 14%
	(S_0S_0) / ($S_0S_0S_0S_0$)	1.02 x 10^7 s^{-1} / 49%	1.01 x 10^7 s^{-1} / 45%
(T_1+T_1) / (T_1+T_1+S_0+S_0)	($P^{\bullet+}$+S_0) / ($P^{\bullet+}$+S_0+S_0+S_0)	5.56 x 10^6 s^{-1} / 100%	4.16 x 10^6 s^{-1} / 100%
($P^{\bullet+}$+S_0) / ($P^{\bullet+}$+S_0+S_0+S_0)	(S_0S_0) / ($S_0S_0S_0S_0$)	3.49 x 10^5 s^{-1} / 100%	3.30 x 10^5 s^{-1} / 100%

[a] See **Figure 6** and **Figure S40** for the corresponding target analyses.

[b] The quantum yield is always given with respect to the initial state on the left side of the corresponding row. It is calculated from the target analysis as the ratio of the corresponding rate and the sum over all rates that lead to a deactivation of the corresponding initial state.

Electrochemistry

Table S15. Basic electrochemical properties for **PM**, **PD**, **PT** and **TCNQ** (vs. Fc/Fc^+).

Molecule	E_{Ox1}	E_{Red1}
**PM**	0.30 V	–1.55 V
**PD**	0.29 V	–1.58 V
**PT**	0.30 V	–1.59 V
**TCNQ**	---	–0.55 V

See **Figures S41** for the raw data.

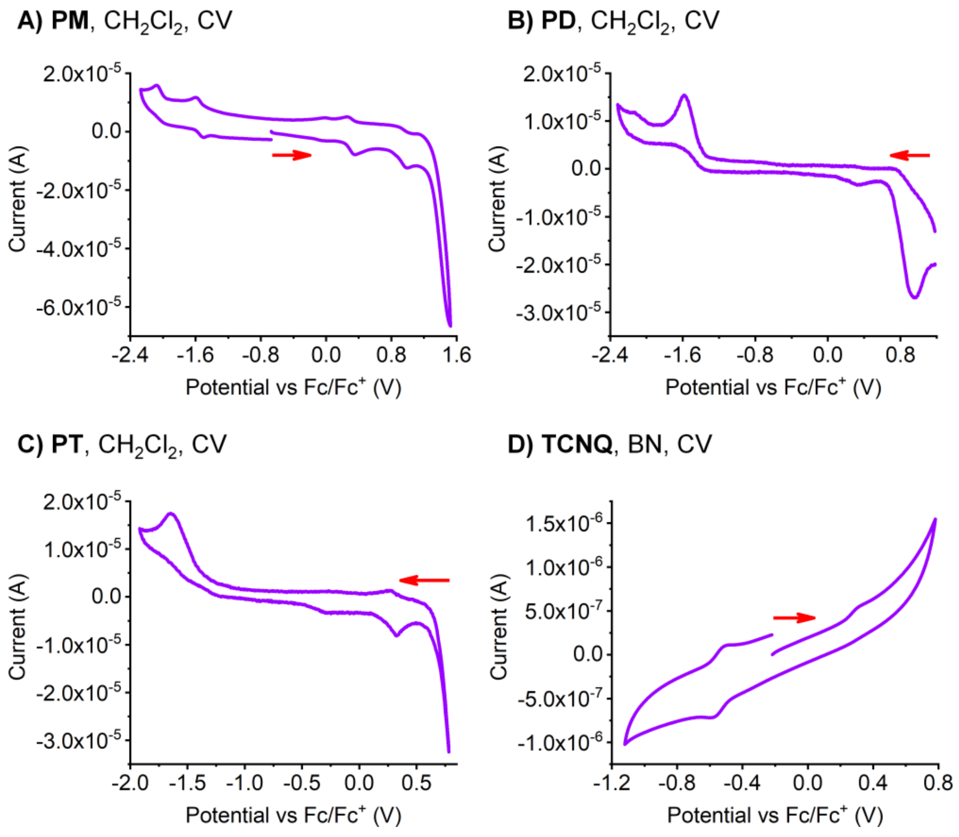

Figure S41. Cyclic voltammograms of **PM**, **PD**, **PT** and **TCNQ**.
A) CV of **PM** recorded in argon saturated dichloromethane (CH_2Cl_2) with a scan rate of 0.10 V/s. B / C) CV of **PD** / **PT** recorded in argon saturated dichloromethane (CH_2Cl_2) with a scan rate of 0.15 V/s. D) CV of **TCNQ** recorded in argon saturated benzonitrile (BN) with a scan rate of 0.10 V/s. Tetrabutylammonium hexafluorophosphate (0.1 M) was used as supporting electrolyte. The scan direction is indicated by red arrows. The voltammograms are corrected against the ferrocene/ferrocenium (Fc/Fc^+) redox couple.

Supplemental computational results

Table S16: B3LYP-D3/6-31G(d) optimized geometry of **PT**.

Center	Atomic Nr.	Coordinates (Å)		
		x	y	z
1	14	9.0157	7.4542	4.8443
2	6	3.6646	2.9406	2.6449
3	1	2.4225	0.2111	-0.6451
4	6	0.0240	-0.4083	1.7983
5	1	0.6570	-1.0312	2.4404
6	1	-0.5956	0.2137	2.4534
7	6	4.5190	5.2323	3.0798
8	6	0.9115	0.4913	0.8975
9	6	4.8978	2.3896	3.0909
10	6	3.4603	4.3484	2.6300
11	6	5.9645	3.2731	3.5214
12	6	1.8101	1.3462	1.6701
13	6	5.7574	4.6819	3.5178
14	6	2.2515	4.9139	2.1713
15	1	1.4624	4.2493	1.8328
16	6	2.6532	2.0798	2.1463
17	6	5.1201	0.9957	3.1116
18	1	4.3140	0.3366	2.8032
19	6	7.1938	2.7053	3.9184
20	1	7.9958	3.3688	4.2278
21	6	7.4084	1.3312	3.9166
22	6	3.0989	7.1717	2.5985
23	6	4.2941	6.6252	3.0529
24	1	5.0860	7.2859	3.3928
25	6	2.0445	6.2879	2.1390
26	6	6.7973	5.5472	3.9429
27	6	6.3321	0.4462	3.5137
28	6	8.6654	0.7521	4.2939
29	1	9.4732	1.4175	4.5889
30	6	6.5641	-0.9685	3.5269
31	1	5.7576	-1.6277	3.2165
32	6	0.8223	6.8666	1.6618
33	1	0.0343	6.2053	1.3102
34	6	8.8439	-0.6033	4.2831
35	1	9.8016	-1.0298	4.5684
36	6	2.8704	8.5871	2.5627
37	1	3.6620	9.2471	2.9092
38	6	1.6873	9.0951	2.1036
39	1	1.5258	10.1694	2.0825
40	6	7.7765	-1.4765	3.8983
41	1	7.9439	-2.5497	3.8920
42	6	7.6905	6.2988	4.3079

43	6	0.6483	8.2223	1.6449
44	1	-0.2813	8.6446	1.2753
45	6	10.5861	7.0759	3.8656
46	6	8.4362	9.2181	4.4977
47	6	9.3251	7.2179	6.6923
48	14	-9.7805	-6.5087	5.7520
49	6	-3.8167	-3.3366	2.8886
50	1	-2.4154	0.2189	0.6522
51	6	-0.0001	-2.2007	0.0045
52	1	-0.6368	-2.8489	-0.6077
53	1	0.6346	-2.8504	0.6172
54	6	-6.2865	-3.5896	3.0097
55	6	-0.8846	-1.3102	0.9183
56	6	-3.6594	-4.3283	3.8932
57	6	-5.1138	-2.9527	2.4403
58	6	-4.8314	-4.9608	4.4685
59	6	-1.7891	-2.1204	1.7326
60	6	-6.1281	-4.5840	4.0171
61	6	-5.2955	-1.9645	1.4487
62	1	-4.4206	-1.4819	1.0263
63	6	-2.6850	-2.7107	2.3027
64	6	-2.3822	-4.7176	4.3497
65	1	-1.5087	-4.2406	3.9144
66	6	-4.6486	-5.9416	5.4661
67	1	-5.5263	-6.4143	5.8966
68	6	-3.3856	-6.3171	5.9097
69	6	-7.7267	-2.2336	1.5593
70	6	-7.5613	-3.2036	2.5422
71	1	-8.4372	-3.6847	2.9671
72	6	-6.5554	-1.5891	0.9971
73	6	-7.2749	-5.2042	4.5750
74	6	-2.2141	-5.6853	5.3329
75	6	-3.1973	-7.3137	6.9236
76	1	-4.0774	-7.7854	7.3540
77	6	-0.9186	-6.0844	5.8012
78	1	-0.0428	-5.6087	5.3665
79	6	-6.7383	-0.5889	-0.0141
80	1	-5.8612	-0.1079	-0.4404
81	6	-1.9433	-7.6631	7.3397
82	1	-1.8137	-8.4197	8.1088
83	6	-9.0195	-1.8457	1.0747
84	1	-9.8965	-2.3316	1.4957
85	6	-9.1459	-0.8906	0.1046
86	1	-10.1299	-0.6081	-0.2596
87	6	-0.7866	-7.0392	6.7699
88	1	0.2003	-7.3333	7.1166
89	6	-8.2684	-5.7347	5.0524

90	6	-7.9895	-0.2522	-0.4478
91	1	-8.1138	0.4941	-1.2263
92	6	-9.7378	-8.3648	5.4044
93	6	-11.2845	-5.7229	4.9219
94	6	-9.8238	-6.1957	7.6141
95	14	9.7608	-6.5526	-5.7413
96	6	3.8114	-3.3526	-2.8791
97	1	2.4322	-1.0404	0.6010
98	6	-0.0188	-0.4096	-1.7907
99	1	0.6027	0.2101	-2.4463
100	1	-0.6536	-1.0310	-2.4324
101	6	6.2801	-3.6167	-2.9998
102	6	0.8871	-1.3136	-0.9100
103	6	3.6497	-4.3438	-3.8834
104	6	5.1102	-2.9743	-2.4307
105	6	4.8190	-4.9818	-4.4584
106	6	1.7889	-2.1273	-1.7237
107	6	6.1173	-4.6107	-4.0069
108	6	5.2962	-1.9867	-1.4394
109	1	4.4233	-1.5000	-1.0173
110	6	2.6824	-2.7216	-2.2934
111	6	2.3708	-4.7276	-4.3400
112	1	1.4994	-4.2465	-3.9049
113	6	4.6319	-5.9621	-5.4558
114	1	5.5075	-6.4388	-5.8860
115	6	3.3672	-6.3320	-5.8994
116	6	7.7261	-2.2666	-1.5499
117	6	7.5565	-3.2361	-2.5325
118	1	8.4303	-3.7213	-2.9571
119	6	6.5577	-1.6168	-0.9879
120	6	7.2614	-5.2362	-4.5645
121	6	2.1985	-5.6948	-5.3229
122	6	3.1746	-7.3280	-6.9130
123	1	4.0526	-7.8038	-7.3432
124	6	0.9013	-6.0882	-5.7912
125	1	0.0276	-5.6084	-5.3568
126	6	6.7450	-0.6170	0.0230
127	1	5.8699	-0.1320	0.4491
128	6	1.9191	-7.6719	-7.3292
129	1	1.7861	-8.4281	-8.0981
130	6	9.0206	-1.8842	-1.0653
131	1	9.8955	-2.3740	-1.4862
132	6	9.1511	-0.9293	-0.0956
133	1	10.1364	-0.6509	0.2684
134	6	0.7652	-7.0426	-6.7597
135	1	-0.2230	-7.3324	-7.1064
136	6	8.2524	-5.7715	-5.0417

137	6	7.9976	-0.2857	0.4566
138	1	8.1251	0.4605	1.2347
139	6	9.8036	-6.2430	-7.6041
140	6	11.2687	-5.7714	-4.9140
141	6	9.7112	-8.4079	-5.3905
142	14	-9.0424	7.3884	-4.8864
143	6	-3.6463	2.9508	-2.6432
144	1	-2.4288	-1.0335	-0.5929
145	6	0.0053	1.3770	0.0032
146	1	-0.6159	2.0260	0.6306
147	1	0.6285	2.0237	-0.6248
148	6	-5.9439	3.2915	-3.5225
149	6	-0.9035	0.4934	-0.8905
150	6	-3.4355	4.3576	-2.6320
151	6	-4.8816	2.4043	-3.0888
152	6	-4.4896	5.2453	-3.0854
153	6	-1.7992	1.3504	-1.6640
154	6	-5.7299	4.6996	-3.5229
155	6	-5.1103	1.0113	-3.1058
156	1	-4.3075	0.3494	-2.7950
157	6	-2.6391	2.0867	-2.1421
158	6	-2.2246	4.9189	-2.1736
159	1	-1.4390	4.2516	-1.8323
160	6	-4.2583	6.6371	-3.0623
161	1	-5.0471	7.3000	-3.4051
162	6	-3.0610	7.1795	-2.6084
163	6	-7.3964	1.3552	-3.9133
164	6	-7.1756	2.7283	-3.9188
165	1	-7.9747	3.3941	-4.2307
166	6	-6.3245	0.4663	-3.5074
167	6	-6.7679	5.5647	-3.9531
168	6	-2.0113	6.2920	-2.1451
169	6	-2.8256	8.5938	-2.5765
170	1	-3.6135	9.2567	-2.9258
171	6	-0.7869	6.8662	-1.6682
172	1	-0.0024	6.2021	-1.3137
173	6	-6.5632	-0.9473	-3.5170
174	1	-5.7601	-1.6095	-3.2043
175	6	-1.6406	9.0974	-2.1176
176	1	-1.4740	10.1711	-2.0994
177	6	-8.6560	0.7812	-4.2899
178	1	-9.4604	1.4497	-4.5870
179	6	-8.8409	-0.5733	-4.2756
180	1	-9.8004	-0.9962	-4.5604
181	6	-0.6063	8.2211	-1.6551
182	1	0.3249	8.6400	-1.2856
183	6	-7.6683	6.3041	-4.3256

184	6	-7.7778	-1.4505	-3.8879
185	1	-7.9502	-2.5229	-3.8789
186	6	-9.3640	8.6985	-3.5647
187	6	-10.5769	6.3147	-5.1323
188	6	-8.5509	8.2096	-6.5144
189	6	1.7786	-0.4121	-0.0150
190	6	-1.7734	-0.4067	0.0226
191	1	10.4201	7.1968	2.7893
192	1	10.9248	6.0481	4.0395
193	1	11.3981	7.7521	4.1605
194	1	8.2382	9.3663	3.4300
195	1	9.1985	9.9454	4.8031
196	1	7.5151	9.4465	5.0462
197	1	10.1126	7.8969	7.0422
198	1	9.6416	6.1925	6.9144
199	1	8.4196	7.4228	7.2744
200	1	10.6965	-6.6969	-8.0517
201	1	9.8229	-5.1701	-7.8259
202	1	8.9244	-6.6716	-8.0984
203	1	11.3039	-4.6913	-5.0965
204	1	12.1966	-6.2090	-5.3024
205	1	11.2505	-5.9292	-3.8296
206	1	9.6773	-8.6060	-4.3133
207	1	10.6021	-8.9025	-5.7974
208	1	8.8300	-8.8748	-5.8450
209	1	-10.6310	-8.8552	5.8111
210	1	-8.8589	-8.8344	5.8606
211	1	-9.7036	-8.5648	4.3276
212	1	-11.3156	-4.6424	5.1025
213	1	-12.2145	-6.1562	5.3102
214	1	-11.2659	-5.8828	3.8378
215	1	-8.9468	-6.6270	8.1101
216	1	-10.7190	-6.6454	8.0616
217	1	-9.8391	-5.1223	7.8342
218	1	-8.4758	9.3175	-3.3942
219	1	-9.6399	8.2376	-2.6096
220	1	-10.1832	9.3620	-3.8689
221	1	-10.3993	5.5382	-5.8851
222	1	-11.4266	6.9214	-5.4689
223	1	-10.8663	5.8191	-4.1986
224	1	-8.3506	7.4619	-7.2900
225	1	-7.6481	8.8193	-6.3954
226	1	-9.3544	8.8638	-6.8752

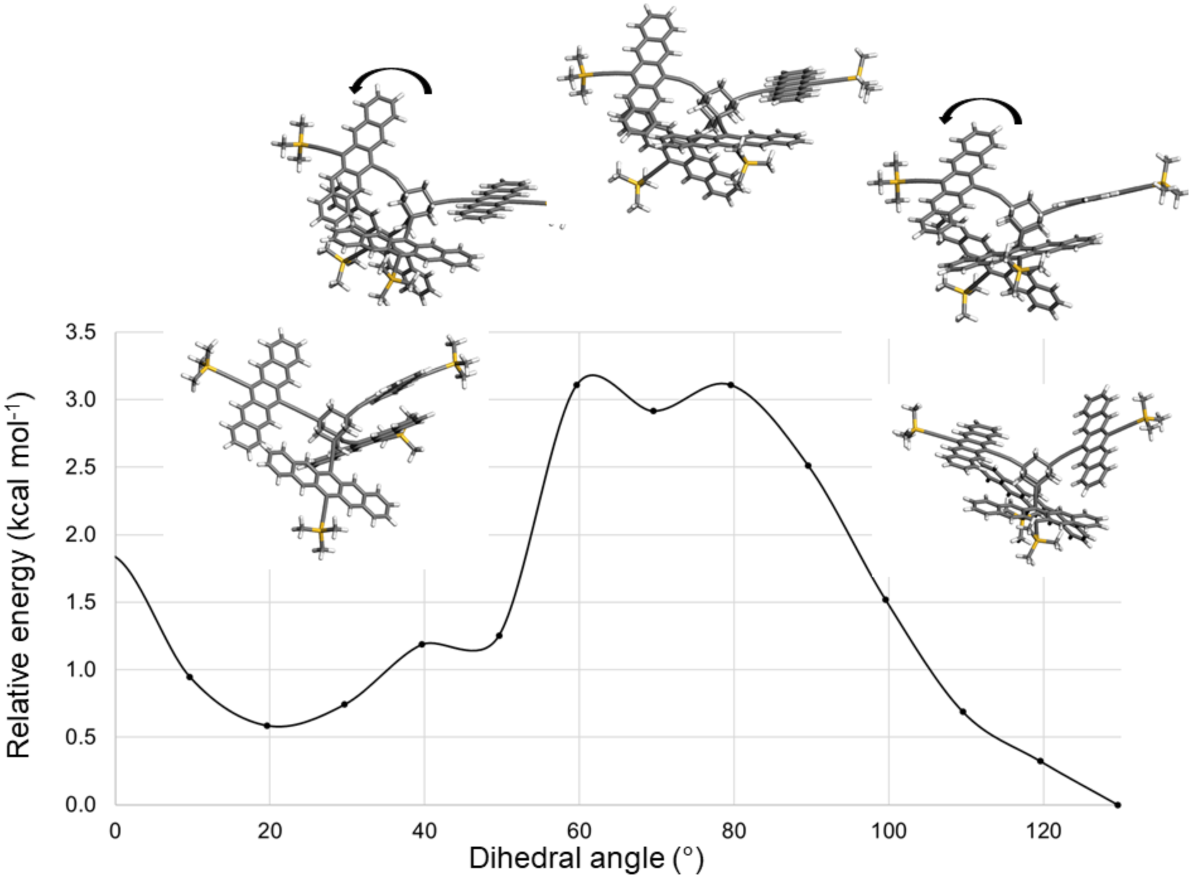

Figure S42: Energy profile and optimized geometries for the rotation of one pentacene moiety in **PT**.
The weak stabilizing interactions between the pentacene moieties lead to a low total barrier (3.1 kcal mol^{-1}) and some shallow minima in addition to the most stable. Thus, because inter-pentacene interactions are stabilizing in almost all configurations along the rearrangement path, the rearrangement of the pentacenes among themselves is facile.

Excited states

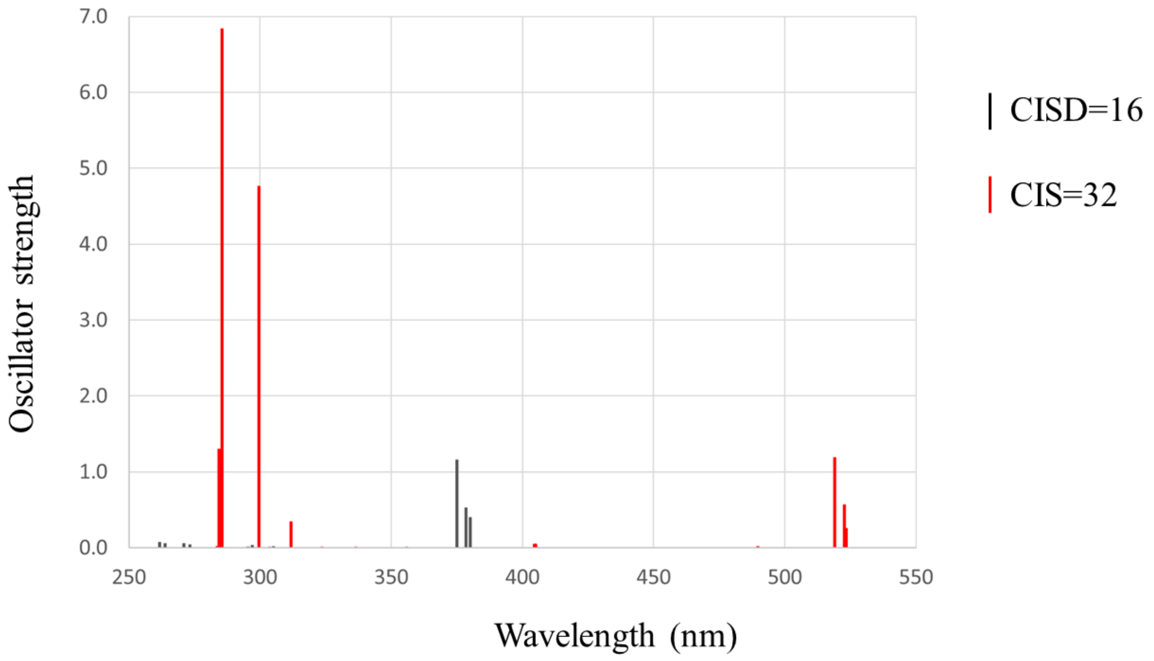

Figure S43: AM1/CIS and AM1/CISD calculated vertical excitation energies (eV) and oscillator strengths for **PT** using eight occupied and eight virtual, orbitals as the active space and with a polarizable continuum-model solvation treatment for benzonitrile.[18]
Excited states were calculated at the B3LYP-D3/6-31G(d) optimized geometry of the most stable minimum. The calculated excitation energies are shifted to higher wavelengths by about 100 nm compared to experiment, so that the weak $S_0 \rightarrow S_1$ transition (calculated at 920 nm) is unlikely to be seen.

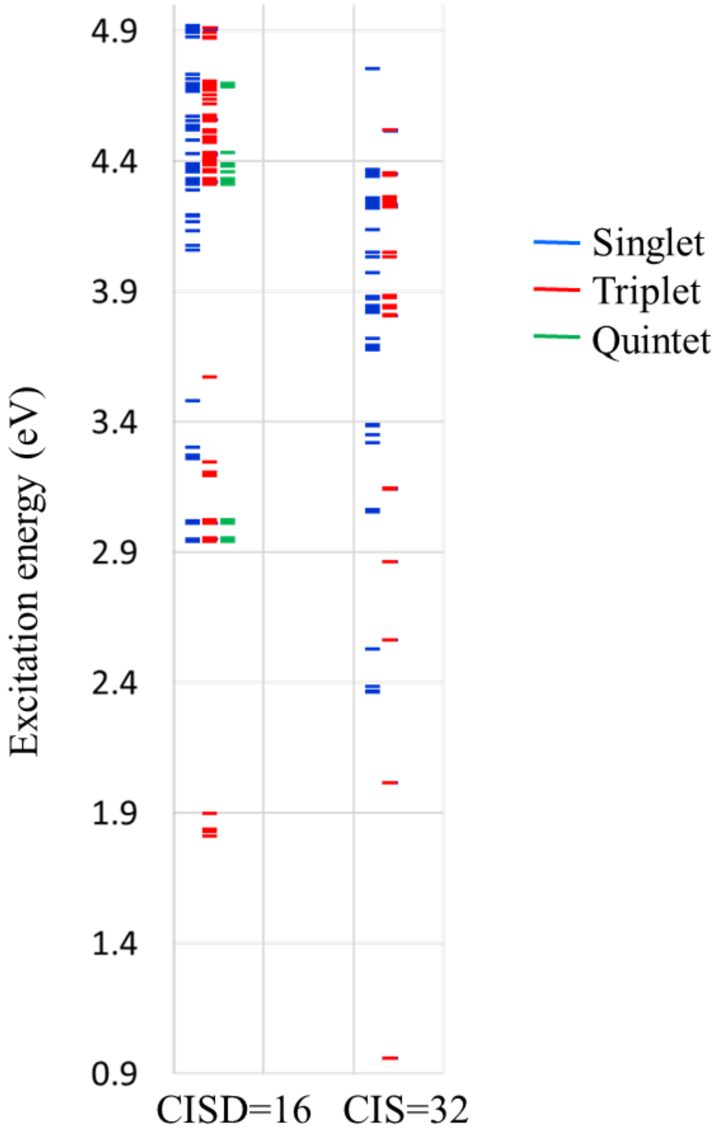

Figure S44: AM1/CIS and AM1/CISD calculated vertical excitation energies (eV) up to an excitation energy of 5 eV for **PT** using eight occupied and eight virtual, orbitals as the active space and with a polarizable continuum-model solvation treatment for benzonitrile. See **Table S17** for the detailed state energies.

Table S17: AM1/CISD calculated vertical excitation energies (eV) for **PT** using eight occupied and eight virtual, orbitals as the active space and with a polarizable continuum model solvation treatment for benzonitrile.[a, b]

**CISD=16**						**CIS=32**					
**State**	**eV**	**nm**	**Del Mu**	**Osc.**	**Multip.**	**State**	**eV**	**nm**	**Del Mu**	**Osc.**	**Multip.**
S_0	0.000	0.0			Singlet	S_0	0.000	0.0			Singlet
T_1	1.813	683.7	0.21		Triplet	T_1	0.961	1290.4	0.08		Triplet
T_2	1.829	678.0	0.28		Triplet	T_2	0.961	1289.9	0.10		Triplet
T_3	1.839	674.1	0.30		Triplet	T_3	0.962	1289.0	0.08		Triplet
T_4	1.900	652.6	0.32		Triplet	T_4	0.962	1288.5	0.14		Triplet
Q_1	2.943	421.2	0.15		Quintet						
T_5	2.944	421.2	0.15		Triplet	T_5	2.019	614.0	0.10		Triplet
S_1	2.944	421.2	0.15	0.000	Singlet	T_6	2.019	614.0	0.08		Triplet
Q_2	2.953	419.8	0.28		Quintet						
S_2	2.954	419.8	0.06	0.000	Singlet	T_7	2.019	613.9	0.07		Triplet
T_6	2.954	419.8	0.06		Triplet	T_8	2.020	613.9	0.16		Triplet
Q_3	2.954	419.8	0.07		Quintet						
T_7	2.954	419.7	0.30		Triplet	S_1	2.368	523.5	0.04	0.260	Singlet
S_3	2.955	419.6	0.31	0.000	Singlet	S_2	2.372	522.7	0.05	0.571	Singlet
Q_4	3.012	411.6	0.43		Quintet						
T_8	3.014	411.3	0.42		Triplet	S_3	2.389	518.9	0.04	1.193	Singlet
Q_5	3.015	411.2	0.29		Quintet						
T_9	3.015	411.2	0.29		Triplet	S_4	2.532	489.6	0.07	0.019	Singlet
S_4	3.015	411.2	0.29	0.000	Singlet	T_9	2.565	483.4	0.05		Triplet
S_5	3.016	411.1	0.41	0.000	Singlet	T_{10}	2.565	483.4	0.09		Triplet
Q_6	3.025	409.9	0.23		Quintet						
T_{10}	3.025	409.9	0.23		Triplet	T_{11}	2.565	483.3	0.02		Triplet
S_6	3.025	409.9	0.23	0.000	Singlet	T_{12}	2.565	483.3	0.02		Triplet
T_{11}	3.193	388.3	0.16		Triplet	T_{13}	2.865	432.7	0.05		Triplet
T_{12}	3.208	386.5	0.18		Triplet	T_{14}	2.865	432.7	0.08		Triplet
T_{13}	3.248	381.8	0.54		Triplet	T_{15}	2.866	432.6	0.10		Triplet
S_7	3.262	380.0	0.85	0.405	Singlet	T_{16}	2.866	432.6	0.02		Triplet
S_8	3.276	378.4	0.83	0.532	Singlet	S_5	3.058	405.5	0.03	0.007	Singlet
S_9	3.306	375.0	0.27	1.163	Singlet	S_6	3.061	405.0	0.06	0.047	Singlet
S_{10}	3.483	356.0	0.27	0.010	Singlet	S_7	3.064	404.6	0.04	0.053	Singlet
T_{14}	3.573	347.0	3.49		Triplet	S_8	3.065	404.5	0.11	0.019	Singlet
S_{11}	4.062	305.2	0.97	0.015	Singlet	T_{17}	3.145	394.2	0.12		Triplet
S^{12}	4.081	303.8	1.19	0.012	Singlet	T_{18}	3.146	394.1	0.10		Triplet
S_{13}	4.137	299.7	0.10	0.006	Singlet	T_{19}	3.148	393.8	0.10		Triplet
S_{14}	4.173	297.1	0.21	0.034	Singlet	T_{20}	3.149	393.7	0.08		Triplet
S_{15}	4.194	295.6	0.31	0.013	Singlet	S^9	3.325	372.8	0.06	0.001	Singlet
S_{16}	4.199	295.2	0.75	0.009	Singlet	S_{10}	3.354	369.7	0.14	0.000	Singlet
S_{17}	4.293	288.8	1.28	0.002	Singlet	S_{11}	3.388	366.0	0.07	0.005	Singlet
Q_7	4.312	287.5	0.16		Quintet						
T_{15}	4.313	287.5	0.17		Triplet	S_{12}	3.394	365.3	0.08	0.001	Singlet
S_{18}	4.313	287.5	0.17	0.000	Singlet	S_{13}	3.678	337.0	0.29	0.002	Singlet
Q_8	4.322	286.9	0.35		Quintet						
Q_9	4.322	286.9	0.23		Quintet						

T_{16}	4.322	286.9	0.23		Triplet	S_{14}	3.686	336.4	0.06	0.009	Singlet
S_{19}	4.322	286.9	0.23	0.000	Singlet	S_{15}	3.697	335.4	0.08	0.002	Singlet
T_{17}	4.323	286.8	0.35		Triplet	S_{16}	3.725	332.8	0.18	0.001	Singlet
S_{20}	4.323	286.8	0.35	0.000	Singlet	T_{21}	3.808	325.6	4.78		Triplet
Q_{10}	4.332	286.2	0.14		Quintet						
T_{18}	4.334	286.1	0.17		Triplet	T_{22}	3.809	325.5	2.98		Triplet
S_{21}	4.335	286.0	0.19	0.000	Singlet	T_{23}	3.810	325.4	0.27		Triplet
Q_{11}	4.361	284.3	0.72		Quintet						
T_{19}	4.361	284.3	0.72		Triplet	T_{24}	3.811	325.4	0.17		Triplet
S_{22}	4.361	284.3	0.72	0.000	Singlet	S_{17}	3.824	324.2	6.08	0.004	Singlet
Q_{12}	4.362	284.2	0.75		Quintet						
T_{20}	4.367	283.9	0.86		Triplet	S_{18}	3.827	324.0	7.34	0.002	Singlet
S_{23}	4.370	283.7	0.91	0.000	Singlet	S_{19}	3.831	323.6	1.82	0.008	Singlet
Q_{13}	4.382	282.9	0.25		Quintet						
T_{21}	4.385	282.8	0.22		Triplet	S_{20}	3.832	323.5	2.29	0.003	Singlet
S_{24}	4.386	282.7	0.20	0.000	Singlet	T_{25}	3.838	323.0	37.53		Triplet
Q_{14}	4.392	282.3	0.60		Quintet						
T_{22}	4.393	282.2	0.60		Triplet	S_{21}	3.841	322.8	26.94	0.002	Singlet
S_{25}	4.393	282.2	0.60	0.000	Singlet	T_{26}	3.846	322.3	39.37		Triplet
T_{23}	4.404	281.5	0.10		Triplet	S_{22}	3.848	322.2	35.09	0.002	Singlet
T_{24}	4.409	281.2	0.16		Triplet	T_{27}	3.876	319.8	42.86		Triplet
T_{25}	4.417	280.7	0.15		Triplet	S_{23}	3.876	319.8	42.64	0.002	Singlet
T_{26}	4.427	280.1	0.18		Triplet	T_{28}	3.886	319.0	42.98		Triplet
T_{27}	4.428	280.0	0.28		Triplet	S_{24}	3.886	319.0	42.81	0.002	Singlet
Q_{15}	4.432	279.8	0.85		Quintet						
T_{28}	4.432	279.7	0.85		Triplet	S_{25}	3.976	311.8	0.07	0.350	Singlet
T_{29}	4.433	279.7	0.20		Triplet	T_{29}	4.035	307.3	50.14		Triplet
S_{26}	4.433	279.7	0.85	0.000	Singlet	S_{26}	4.035	307.3	49.98	0.001	Singlet
T_{30}	4.475	277.0	2.78		Triplet	T_{30}	4.052	306.0	50.13		Triplet
T_{31}	4.480	276.7	27.51		Triplet	S_{27}	4.052	306.0	50.04	0.001	Singlet
S_{27}	4.485	276.4	40.47	0.002	Singlet	S_{28}	4.140	299.5	0.02	4.767	Singlet
T_{32}	4.494	275.9	7.81		Triplet	T_{31}	4.225	293.5	24.48		Triplet
T_{33}	4.512	274.8	22.79		Triplet	S_{29}	4.225	293.4	23.81	0.000	Singlet
T_{34}	4.522	274.2	40.49		Triplet	T_{32}	4.227	293.3	24.46		Triplet
S_{28}	4.524	274.1	41.07	0.001	Singlet	S_{30}	4.228	293.3	23.90	0.000	Singlet
S_{29}	4.534	273.4	37.18	0.041	Singlet	T_{33}	4.237	292.6	2.21		Triplet
S_{30}	4.542	272.9	0.79	0.002	Singlet	T_{34}	4.237	292.6	25.33		Triplet
T_{35}	4.554	272.2	17.11		Triplet	T_{35}	4.237	292.6	0.41		Triplet
T_{36}	4.558	272.0	36.24		Triplet	T_{36}	4.238	292.6	0.68		Triplet
S_{31}	4.560	271.9	42.01	0.003	Singlet	S_{31}	4.238	292.6	58.64	0.000	Singlet
T_{37}	4.563	271.7	0.19		Triplet	T_{37}	4.238	292.5	30.61		Triplet
T_{38}	4.565	271.6	3.93		Triplet	T_{38}	4.247	292.0	56.03		Triplet
T_{39}	4.576	270.9	0.84		Triplet	S_{32}	4.247	291.9	56.76	0.000	Singlet
S_{32}	4.577	270.9	4.02	0.059	Singlet	T_{39}	4.255	291.4	55.98		Triplet
T_{40}	4.623	268.2	3.79		Triplet	S_{33}	4.256	291.3	56.30	0.001	Singlet
T_{41}	4.637	267.3	0.27		Triplet	T_{40}	4.264	290.8	58.12		Triplet
T_{42}	4.654	266.4	3.94		Triplet	S_{34}	4.264	290.8	58.37	0.001	Singlet

S_{33}	4.673	265.3	45.57	0.001	Singlet	S_{35}	4.343	285.5	0.04	6.843	Singlet
T_{43}	4.674	265.3	48.55		Triplet	T_{41}	4.348	285.1	0.16		Triplet
S_{34}	4.678	265.0	45.66	0.001	Singlet	T_{42}	4.352	284.9	0.22		Triplet
T_{44}	4.678	265.0	48.58		Triplet	T_{43}	4.354	284.8	0.30		Triplet
Q_{16}	4.686	264.6	3.46		Quintet						
Q_{17}	4.690	264.3	3.57		Quintet						
T_{45}	4.691	264.3	3.57		Triplet	T_{44}	4.356	284.6	0.41		Triplet
S_{35}	4.692	264.3	3.56	0.000	Singlet	S_{36}	4.360	284.4	0.17	1.306	Singlet
S_{36}	4.699	263.9	0.42	0.059	Singlet	S_{37}	4.365	284.0	0.11	0.014	Singlet
Q_{18}	4.700	263.8	3.60		Quintet						
T_{46}	4.701	263.8	3.60		Triplet	S_{38}	4.366	284.0	0.13	0.017	Singlet
S_{36}	4.701	263.7	3.60	0.000	Singlet	S_{39}	4.370	283.7	0.12	0.020	Singlet
T_{47}	4.707	263.4	3.31		Triplet	S_{40}	4.373	283.5	0.13	0.002	Singlet
S_{38}	4.718	262.8	3.21	0.001	Singlet	T_{45}	4.519	274.4	0.19		Triplet
S_{39}	4.735	261.8	1.05	0.077	Singlet	T_{46}	4.519	274.3	0.11		Triplet
T_{48}	4.874	254.4	1.62		Triplet	T_{47}	4.520	274.3	0.14		Triplet
S_{40}	4.878	254.2	57.27	0.000	Singlet	T_{48}	4.522	274.2	0.12		Triplet
T_{49}	4.878	254.1	57.22		Triplet	S_{41}	4.759	260.5	0.26	0.001	Singlet
S_{41}	4.895	253.3	56.84	0.000	Singlet	S_{42}	4.759	260.5	0.46	0.001	Singlet
T_{50}	4.896	253.2	56.74		Triplet	S_{43}	4.760	260.5	0.40	0.000	Singlet
S_{42}	4.909	252.5	54.76	0.000	Singlet	S_{44}	4.760	260.4	0.05	0.000	Singlet
T_{51}	4.910	252.5	53.46		Triplet						
T_{52}	4.911	252.4	9.59		Triplet						
S_{43}	4.912	252.4	53.34	0.000	Singlet						
T_{53}	4.912	252.4	44.33		Triplet						
S_{44}	4.913	252.3	54.56	0.000	Singlet						
T_{54}	4.913	252.3	54.21		Triplet						
S_{45}	4.925	251.7	56.16	0.000	Singlet						

[a] **PT** exhibits four weakly split lowest triplet states between 1.893 and 1.900 eV. This pattern recurs for the singlet excited states, for instance for S_8-S_{11} and S_{14}-S_{17}. Groups of three equivalent singlet, triplet and quintet states occur frequently (for instance $Q_1/T_5/S_2$, Q_2/S_3, T_6 and $Q_3/T_7/S_4$). Note that, because of the strong stabilization of the ground state in the CISD calculation, all excitation energies are too high. This ground-state stabilization is not possible for CIS calculations, which give results closer to experiment but cannot reproduce quintet states.

[b] The occurrence of weakly split quadruplets of states coupled with the facile conformational rearrangement of **PT** leads to a complex situation in which the states can interchange easily and for which adiabatic states are most likely a poor approximation.

References

[1] B. S. Basel, J. Zirzlmeier, C. Hetzer, B. T. Phelan, M. D. Krzyaniak, S. R. Reddy, P. B. Coto, N. E. Horwitz, R. M. Young, F. J. White, F. Hampel, T. Clark, M. Thoss, R. R. Tykwinski, M. R. Wasielewski, D. M. Guldi, *Nat. Commun.* **2017**, *8*, 15171.

[2] D. Lehnherr, A. H. Murray, R. McDonald, R. R. Tykwinski, *Angew. Chem. Int. Ed.* **2010**, *49*, 6190-6194.

[3] S. L. Murov, I. Carmichael, G. L. Hug, *Handbook of Photochemistry*, 2 ed., Marcel Dekker, **1993**.

[4] a) J. J. Snellenburg, S. Laptenok, R. Seger, K. M. Mullen, I. H. M. van Stokkum, *J. Stat. Softw.* **2012**, *49*, 22; b) K. M. Mullen, I. H. M. van Stokkum, *J. Stat. Softw.* **2007**, *18*, 46; c) I. H. M. van Stokkum, D. S. Larsen, R. van Grondelle, *Biochim. Biophys. Acta - Bioenergetics* **2004**, *1657*, 82-104.

[5] B. S. Basel, J. Zirzlmeier, C. Hetzer, S. R. Reddy, B. T. Phelan, M. D. Krzyaniak, P. B. Coto, M. K. Volland, R. M. Young, T. Clark, M. Thoss, R. R. Tykwinski, M. R. Wasielewski, D. M. Guldi, *Chem* **2018**, *4*, 1092–1111.

[6] V. Balzani, P. Ceroni, A. Juris, *Photochemistry and Photophysics: Concepts, Research, Applications*, Wiley-VCH, **2014**.

[7] A. D. Becke, *J. Chem. Phys.* **1993**, *98*, 5648-5652.

[8] R. Ditchfield, W. J. Hehre, J. A. Pople, *J. Chem. Phys.* **1971**, *54*, 724-728.

[9] S. Grimme, J. Antony, S. Ehrlich, H. Krieg, *J. Chem. Phys.* **2010**, *132*, 154104.

[10] M. J. S. Dewar, E. G. Zoebisch, E. F. Healy, J. J. P. Stewart, *J. Am. Chem. Soc.* **1985**, *107*, 3902-3909.

[11] Gaussian 16, Revision B.01, Frisch, M. J.; Trucks, G. W.; Schlegel, H. B.; Scuseria, G. E.; Robb, M. A.; Cheeseman, J. R.; Scalmani, G.; Barone, V.; Petersson, G. A.; Nakatsuji, H.; Li, X.; Caricato, M.; Marenich, A. V.; Bloino, J.; Janesko, B. G.; Gomperts, R.; Mennucci, B.; Hratchian, H. P.; Ortiz, J. V.; Izmaylov, A. F.; Sonnenberg, J. L.; Williams-Young, D.; Ding, F.; Lipparini, F.; Egidi, F.; Goings, J.; Peng, B.; Petrone, A.; Henderson, T.; Ranasinghe, D.; Zakrzewski, V. G.; Gao, J.; Rega, N.; Zheng, G.; Liang, W.; Hada, M.; Ehara, M.; Toyota, K.; Fukuda, R.; Hasegawa, J.; Ishida, M.; Nakajima, T.; Honda, Y.; Kitao, O.; Nakai, H.; Vreven, T.; Throssell, K.; Montgomery, J. A., Jr.; Peralta, J. E.; Ogliaro, F.; Bearpark, M. J.; Heyd, J. J.; Brothers, E. N.; Kudin, K. N.; Staroverov, V. N.; Keith, T. A.; Kobayashi, R.; Normand, J.; Raghavachari, K.; Rendell, A. P.; Burant, J. C.; Iyengar, S. S.; Tomasi, J.; Cossi, M.; Millam, J. M.; Klene, M.; Adamo, C.; Cammi, R.; Ochterski, J. W.; Martin, R. L.; Morokuma, K.; Farkas, O.; Foresman, J. B.; Fox, D. J. Gaussian, Inc., Wallingford CT, 2016.

[12] a) M. Hennemann, T. Clark, *J. Mol. Model.* **2014**, *20*, 2331; b) J. T. Margraf, M. Hennemann, B. Meyer, T. Clark, *J. Mol. Model.* **2015**, *21*, 144.

[13] H. Newman, *Synthesis* **1972**, *1972*, 692-693.

[14] K. Nasr, N. Pannier, J. V. Frangioni, W. Maison, *J. Org. Chem.* **2008**, *73*, 1056-1060.

[15] S. I. Kozhushkov, D. S. Yufit, R. Boese, D. Bläser, P. R. Schreiner, A. de Meijere, *Eur. J. Org. Chem.* **2005**, *2005*, 1409-1415.

[16] a) M. Nakazaki, K. Naemura, Y. Hokura, *J. Chem. Soc., Chem. Commun.* **1982**, *0*, 1245-1246; b) K. Naemura, Y. Hokura, M. Nakazaki, *Tetrahedron* **1986**, *42*, 1763-1768.

[17] W. A. Chalifoux, M. J. Ferguson, R. McDonald, F. Melin, L. Echegoyen, R. R. Tykwinski, *J. Phys. Org. Chem.* **2012**, *25*, 69-76.

[18] G. Rauhut, T. Clark, T. Steinke, *J. Am. Chem. Soc.* **1993**, *115*, 9174-9181.

Author Contributions

T. C., D. M. G., and R. R. T. designed the research. B. S. B. performed the photophysical studies. C. H. performed the synthesis of **PD** and **PT**, while S. M. K. synthesized **PM**. B. S. B. and D. M. G. analyzed the photophysical data. T. C. performed the computer chemical calculations. F. H. and F. J. W. measured and solved the crystal structure of **PT**. All authors contributed to the writing and editing of the paper. Overall, C. H. and B. S. B. contributed equally to this publication as lead authors.
